# A Phase IIA Randomized Clinical Trial of a Multiclade HIV-1 DNA Prime Followed by a Multiclade rAd5 HIV-1 Vaccine Boost in Healthy Adults (HVTN204)

**DOI:** 10.1371/journal.pone.0021225

**Published:** 2011-08-03

**Authors:** Gavin J. Churchyard, Cecilia Morgan, Elizabeth Adams, John Hural, Barney S. Graham, Zoe Moodie, Doug Grove, Glenda Gray, Linda-Gail Bekker, M. Juliana McElrath, Georgia D. Tomaras, Paul Goepfert, Spyros Kalams, Lindsey R. Baden, Michelle Lally, Raphael Dolin, William Blattner, Artur Kalichman, J. Peter Figueroa, Jean Pape, Mauro Schechter, Olivier Defawe, Stephen C. De Rosa, David C. Montefiori, Gary J. Nabel, Lawrence Corey, Michael C. Keefer, and the NIAID HIV Vaccine Trials Network

**Affiliations:** 1 Aurum Institute for Health Research, Klerksdorp, South Africa; 2 Centre for AIDS Programme of Research in South Africa (CAPRISA), University of Kwa-Zulu Natal, Durban, South Africa; 3 Statistical Center for HIV/AIDS Research and Prevention, Fred Hutchinson Cancer Research Center, Seattle, Washington, United States of America; 4 Division of AIDS, National Institute of Allergy and Infectious Diseases (NIAID), National Institute of Health (NIH), Bethesda, Maryland, United States of America; 5 Vaccine Research Center, NIAID, NIH, Bethesda, Maryland, United States of America; 6 Perinatal HIV Research Unit, University of the Witwatersrand, Johannesburg, South Africa; 7 Desmond Tutu HIV Foundation, University of Cape Town, Cape Town, South Africa; 8 Duke University Medical Center, Durham, North Carolina, United States of America; 9 University of Alabama, Birmingham, Alabama, United States of America; 10 Vanderbilt University, Nashville, Tennessee, United States of America; 11 Harvard-Brigham and Women's Hospital, Boston, Massachusetts, United States of America; 12 Alpert Medical School of Brown University and Miriam Hospital, Providence, Rhode Island, United States of America; 13 Harvard Medical School- Beth Israel Deaconess Medical Center, Boston, Massachusetts, United States of America; 14 University of Maryland, College Park, Maryland, United States of America; 15 Centro de Referencia e Treinamento em DST/AIDS, Coordenacao dos Institutos de Pesquisa, San Paulo, Brazil; 16 Epidemiology Research and Training Unit (ERTU), Kingston, Jamaica; 17 Cornell-GHESKIO, Institut National de Laboratoire et de Recherches, Port au Prince, Haiti; 18 Projeto Praça Onze, Hospital Escola São Francisco de Assis, Universidade Federal do Rio de Janeiro, Rio de Janeiro, Brazil; 19 Duke Human Vaccine Institute, School of Medicine, Duke University, Durham, North Carolina, United States of America; 20 Department of Medicine, University of Rochester School of Medicine & Dentistry, Rochester, New York, United States of America; University of Cape Town, South Africa

## Abstract

**Background:**

The safety and immunogenicity of a vaccine regimen consisting of a 6-plasmid HIV-1 DNA prime (envA, envB, envC, gagB, polB, nefB) boosted by a recombinant adenovirus serotype-5 (rAd5) HIV-1 with matching inserts was evaluated in HIV-seronegative participants from South Africa, United States, Latin America and the Caribbean.

**Methods:**

480 participants were evenly randomized to receive either: DNA (4 mg IM by Biojector) at 0, 1 and 2 months, followed by rAd5 (10^10^ PU IM by needle/syringe) at 6 months; or placebo. Participants were monitored for reactogenicity and adverse events throughout the 12-month study. Peak and duration of HIV-specific humoral and cellular immune responses were evaluated after the prime and boost.

**Results:**

The vaccine was well tolerated and safe. T-cell responses, detected by interferon-γ (IFN-γ) ELISpot to global potential T-cell epitopes (PTEs) were observed in 70.8% (136/192) of vaccine recipients overall, most frequently to Gag (54.7%) and to Env (54.2%). In U.S. vaccine recipients T-cell responses were less frequent in Ad5 sero-positive versus sero-negative vaccine recipients (62.5% versus 85.7% respectively, p = 0.035). The frequency of HIV-specific CD4+ and CD8+ T-cell responses detected by intracellular cytokine staining were similar (41.8% and 47.2% respectively) and most secreted ≥2 cytokines. The vaccine induced a high frequency (83.7%–94.6%) of binding antibody responses to consensus Group M, and Clades A, B and C gp140 Env oligomers. Antibody responses to Gag were elicited in 46% of vaccine recipients.

**Conclusion:**

The vaccine regimen was well-tolerated and induced polyfunctional CD4+ and CD8+ T-cells and multi-clade anti-Env binding antibodies.

**Trial Registration:**

ClinicalTrials.gov NCT00125970

## Introduction

Control of the HIV pandemic is a major global health priority and it is likely that the development of a safe and effective vaccine to prevent HIV infection and/or HIV-related disease will be needed to achieve this goal [1]. Results from a recently reported phase III study of a combination vaccine regimen conducted in Thailand (RV144) by the Thai Ministry of Public Health and the U.S. Military HIV Research Program has created optimism that a preventive vaccine can be developed, although the efficacy of that regimen was judged to be marginal, short-lived and not sufficient to be useful at a population level [2]. The RV144 regimen consisted of canarypox HIV-gag/protease/envelope boosted by rgp120 B/E protein and produced strong anti-gp120 binding antibodies and T-cell help as demonstrated by lymphoproliferation. It is anticipated that data from this study can provide a framework to inform the development of new vaccine approaches.

A major obstacle to the development of a highly effective vaccine regimen is posed by the marked genetic diversity among global HIV-1 isolates, which is more pronounced in the viral envelope than the internal structural and regulatory proteins [3]. One approach to address viral diversity has been to include immunogens from multiple HIV-1 subtypes in the candidate vaccine preparation. The Dale and Betty Bumpers Vaccine Research Center (VRC) at the U.S. National Institute of Allergy and Infectious Diseases (NIAID) has employed this strategy in the development of a combination vaccine regimen consisting of a 6-plasmid DNA vaccine boosted with a 4-component replication-defective recombinant adenovirus serotype 5 (rAd5) vectors; genes encoding Envelope proteins from subtypes A, B, and C, and a Gag-Pol fusion protein from subtype B are included in each vaccine, and the DNA, but not the rAd5, encodes Nef from subtype B [4], [5], [6], [7], [8]. This regimen has shown promise in SIV challenge studies of a nonhuman primate model, has been shown to be safe and immunogenic in phase I studies and is currently being evaluated for vaccine activity [9], [10].

The objectives of this phase II clinical trial were to evaluate the safety and immunogenicity of the VRC multiclade DNA-HIV prime/rAd5-HIV boost in HIV-1 uninfected healthy adult participants at NIAID HIV Vaccine Trials Network (HVTN) clinical research sites in the Americas (United States, Haiti, Jamaica, and Brazil) and South Africa. The study was conducted in diverse geographic regions in order to evaluate safety and immunogenicity in settings with different circulating HIV clades and prevalence of pre-existing Ad5 immunity. This study is the largest of three phase II trials evaluating the same vaccine regimen. The two other trials were conducted in sub-Saharan Africa only: one funded and implemented by the U.S. Military HIV Research Program (USMHRP protocol RV172) [11] and the other by the International AIDS Vaccine Initiative (IAVI protocol V001) [12]. Our report will provide additional safety and immunogenicity data not evaluated in the RV172 and IAVI V001 clinical trials.

## Methods

### Ethics statement

The study protocol was approved by institutional review boards at each of the participating sites: Universidade Federal do Rio de Janeiro, Hospital Universitário Clementino Fraga Filho Faculdade de Medicina for the Rio, Brazil site; Secretaria de Estado da Saúde, Coordenadoria de Controle de Doenças Centro de Referência e Treinamento DST/AIDS for the Sao Paulo, Brazil site; Comité des Droits Humains des Centres GHESKIO; Ministère de la Santé Publique et de la Population National Ethics Committee and Weill Cornell Medical College, Human Research Protection Programs, Division of Research Integrity for the Port au Prince, Haiti site; Ministry of Health and Environment, Standards & Regulations Division; University of the West Indies Ethics Committee for the Kingston site; University of Alabama at Birmingham IRB for the Birmingham, AL site; Partners Human Research Committee for the Boston, MA (Brigham & Women's) site; Miriam Hospital, Office of Research Administration Communications and Committee Review for the Providence, RI site; Vanderbilt IRB for the Nashville, TN site; University of Rochester Research Subjects Review Board for the Rochester, NY site, University of Maryland, School of Medicine IRB for the Baltimore, MD (IHV) site; University of Kwazulu-Natal, Biomedical Research Ethics Administration for the Klerksdorp, South Africa site; University of Cape Town, Health Sciences Faculty Research Ethics Committee for the Cape Town, South Africa site; and University of the Witwatersrand, Johannesburg, Human Research Ethics Committee for the Soweto, South Africa site. The study was also approved by each country's Regulatory agency and was reviewed by the US Food and Drug Administration before being allowed to proceed. All study participants provided written informed consent prior to participation. The protocol for this trial and supporting CONSRT checklist are available as supporting information; see Protocol S1 and Checklist S1. The trial is registered at ClinicalTrials.gov, registration NCT00125970.

### Study type, population and randomization

Participants were randomized 1∶1 to placebo or vaccine and followed for 12 months after enrollment. Randomization was done by the Statistical Center for HIV/AIDS Research and Prevention using computer generated random numbers stratified by geographic region and done in blocks to ensure balance across groups. The study pharmacist maintained security of the randomization list. All participants and study staff, apart from the study pharmacist, were blinded to treatment assignment.

### Eligibility criteria

Participants were eligible if between 18 and 50 years of age, in good general health based on history, clinical examination and laboratory investigations, practiced behaviors that placed them at low or intermediate risk for HIV acquisition, had no history of receiving investigational products, immunosuppressive medication, blood products, immunoglobulin or vaccines within study-defined periods prior to enrollment. Female participants of childbearing potential were not pregnant or planning to become pregnant and agreed to consistently use contraception for 21 days prior to their first vaccination until 9 months after first vaccination.

### Study product and vaccination schedule

The DNA-HIV vaccine (VRC-HIVDNA-016-00-VP) was manufactured by Vical Incorporated (San Diego, CA) and is composed of six DNA plasmids in equal concentrations that encode Gag, Pol, and Nef from clade B (strains HXB2, NL4-3, NY5/BRU respectively) and HIV-1 Env glycoproteins from clade A (strain 92rw020), clade B (strains HXB2/BaL), and clade C (strain 97ZA012). The DNA placebo was sterile phosphate buffered saline (PBS). Four mg of DNA-HIV or placebo were delivered intramuscularly at 0, 1 and 2 months using the Biojector® 2000 Needle-Free Injection System.

The rAd5-HIV vaccine (VRC-HIVADV014-00-VP, rAd5) was a replication-defective, vaccine containing a mixture of four rAd5 vectors in a 3∶1∶1∶1 ratio that encode the HIV-1 Gag-Pol polyprotein from clade B (strains HXB2-NL4-3) and HIV-1 Env glycoproteins from clades A, B and C matching the DNA Env components. The rAd5-HIV was manufactured by Molecular Medicine (San Diego, CA) under contract to GenVec Incorporated (Gaithersburg, MD). The rAd5-HIV placebo was adenoviral final formulation buffer (*FFB, VRC-DILUENT013-DIL-VP*), that was also used as a diluent for the product. The rAd5-HIV or placebo was delivered intramuscularly using a needle and syringe at 6 months. The dose of rAd5-HIV was 10^10^ PU.

### Safety evaluation

Reactogenicity assessments were performed on all participants for 3 days following each injection. Participants recorded symptoms using a post-vaccination symptom log and were contacted daily by the study site during this time. Reactogenicity was assessed on local symptoms and signs at the injection site (pain, tenderness, erythema, induration and tenderness and enlargement of axillary lymph nodes) and systemic signs (body temperature) and symptoms (malaise and/or fatigue, myalgia, headache, chills, arthralgia, nausea, vomiting). All local and systemic signs, symptoms and laboratory measures of safety were coded according to the Medical Dictionary for Regulatory Activities terminology, and graded using the US Division of AIDS Table for Grading the Severity of Adult and Pediatric Adverse Events as mild, moderate, severe or potentially life-threatening. Adverse events were assessed for their relationship to study product.

Risk reduction counselling was provided at all study visits and women of child bearing potential were advised to avoid pregnancy for 9 months after the last vaccination. Participants were advised not to have HIV testing outside of the study. During the study HIV status was determined using HIV-1 ELISA and HIV RNA PCR. At the end of study, several licensed diagnostic HIV ELISA assays (Abbot HIVAB HIV 1/2 [rDNA], BioRad Genetic System HIV 1/2 Plus O EIA, BioRad Genetic System HIV 1/2 rLAV, bioMerieux Vironostika HIV-1) were performed on sera from HIV-uninfected participants to assess the false positive rate of each assay.

### Immunogenicity analysis

#### Interferon-γ (IFN-γ) ELISpot assay

ELISpot assays were performed on participants' peripheral blood mononuclear cells (PBMCs) cryopreserved within 8 hours of collection using a standardized, validated, bulk IFN-γ ELISpot assay [13] 6 weeks after the fourth vaccination on all available specimens. Global and Clade C potential T-cell epitope (PTE) [14] pools for HIV Env, Gag, Pol and Nef were used at a final concentration of 1.0 µg/ml.

### Intracellular cytokine staining assay

Intracellular cytokine staining (ICS) assays were performed on cryopreserved PBMCs by flow cytometry using PBMCs to determine both HIV-specific and Ad5-specific CD4+ and CD8+ T-cell responses [15] at 6 weeks after the fourth vaccination. For the detection of HIV-specific T cells, thawed PBMC were cultured overnight and then stimulated for six hours with the same HIV-1 peptide pools as for ELISpot [13], [16].

For the detection of Ad5-specific T cells, an “empty” vector lacking HIV-1 gene inserts (provided by Dr. Gary Nabel, NIH Vaccine Research Center, Bethesda, MD) was used to stimulate PBMC. The Ad5 vector was added to PBMC cultures at a ratio of 10,000 Ad5 particle units per cell, six hours later the cells were treated with Brefeldin A, and the ICS assay was performed after an overnight incubation.

Initially eight-color [16] and then 10-color (once validated) [13] ICS assays were used. Criteria for an evaluable response and positivity were based on background measurements and the number of CD4+ and CD8+ cells T cells examined [16]. The frequency and magnitude of CD4+ and CD8+ T-cells producing IFN-γ or interleukin (IL)-2 in response to stimulation with global PTE was reported. The degree of polyfunctionality was evaluated by determining the percent of reactive CD4+ and CD8+ T-cells that produced one or more cytokines (IFN-γ, IL-2 or TNF-α).

### Binding antibodies

Serum HIV-1 specific IgG responses (1/20 dilution) against p55 Clade B Gag (Protein Sciences), Group M Consensus (Con S) gp140 CFI, VRC Clade A gp140, VRC Clade B gp140 and DU123 Clade C gp140 (provided by Dr. L. Liao and B. Haynes, Duke University) were measured at four weeks after the fourth vaccination by a standardized HIV-1 luminex assay as previously described [17], [18]. Antibody measurements were acquired on a Bio-Plex instrument (Bio-Rad) and the readout was expressed as mean fluorescent intensity (MFI). The positive control in each assay was HIV positive sera and the negative control was HIV-negative human sera and blank beads. Samples with blank bead MFI>10,000 were excluded. Samples were determined to be positive if both the MFI and MFI minus blank were greater than 3-fold over the baseline (study visit 2) MFI and MFI minus blank, respectively, and the MFI minus blank was at least 732 MFI (based on the average+3 standard deviations of 25 seronegative plasma samples).

### HIV-1 neutralization assay

Neutralizing antibodies against HIV-1 were measured as a function of reduction in Tat-regulated luciferase (Luc) reporter gene expression after a single round of infection in TZM-bl cells that are permissive to infection to a wide range of HIV strains. Serum and plasma samples were tested at four weeks after the fourth vaccination for an ability to neutralize homologous vaccine strains (92RW020.2, 97ZA012.29, Bal.26) and tier-1 strains that are highly sensitive to neutralization (MN and SF162.LS) [19], [20]. Assays are considered positive if the titer is ≥25. Antibodies that neutralized tier 1 viruses were evaluated further to determine whether they could neutralize a multi-subtype panel of tier 2 viruses. Serum samples from responders were screened at a 1∶10 dilution for neutralizing activity against two subtype A viruses (Q23.17, Q842.d12.), four subtype B viruses (QH0692.42, SC422661.8, PVO.4, AC10.0.29) and four subtype C viruses (Du156.12, Du172.17, Du422.1, ZM197M.PB7). As a control for nonspecific activity, the samples were also assayed against a pseudovirus containing the envelope glycoproteins of murine leukemia virus (MLV).

### Missing data

Samples could not be included in the immunogenicity analysis if: there was insufficient sample quantity; the sample could not be located at the repository; the sample was not collected or collected out of the visit window; the participant was terminated from the study or was HIV-infected at the time of the blood draw.

### Statistical methods

The sample size of 240 vaccine and 240 placebo recipients per group allowed a robust characterization of safety and immunogenicity. Specifically, the sample size of 240 in the vaccine group provided a 90% chance of observing at least one serious adverse event if the true rate of such an event were at least 1%. After accounting for a 10% dropout rate, the sample size of the vaccine group (n = 216) allows a reasonably precise estimate of the immune response rate, with a maximum 95% confidence interval (CI) width of 14%.

To summarize the T-cell response data, positive response rates to any peptide pool and to each individual protein were reported. Positivity of the individual IFN-γ ELISpot responses was determined by a one-sided bootstrap test of the null hypothesis that the experimental well responses were twice those of the background (α = 0.05) [21]. A Westfall-Young approach was used to adjust for the multiple comparisons across peptide pools. Peptide pools with adjusted one-sided p-values≤0.05 were declared positive. In addition, the mean difference in the experimental and negative control wells had to exceed 50 SFC per 10^6^ PBMC for the response to be positive. Positivity of the ICS responses was determined by a one-sided Fisher's exact test applied to each peptide pool-specific response versus the negative control response with a discrete Bonferroni adjustment for the multiple comparisons. Peptide pools with adjusted p-values less than α = 0.00001 were considered positive. Confidence intervals for response rates were calculated with the score test method [22]. Response rates were compared by two-sided Fisher's exact tests. Generalized estimating equations (GEE) for binary data with an unstructured correlation matrix were used to model local and systemic reaction rates separately over time. A GEE model with time, treatment, and a treatment*time interaction term was used to evaluate whether the longitudinal trend in reaction rates differed between vaccine and placebo groups. McNemar's tests were used to compare the CD4+ and CD8+ response rates to the empty Ad5 vector in vaccine and placebo recipients separately. SAS (Version 9.1; SAS Institute) and R (Version 2.11.1) were used for all analyses. The analysis comparing HIV-specific CD4+ and CD8+ T-cell responses by baseline Ad5 status was restricted to U.S. participants only as they had the largest proportion of Ad5 seronegative vaccine recipients and the most complete ICS data available.

## Results

**Figure 1:**
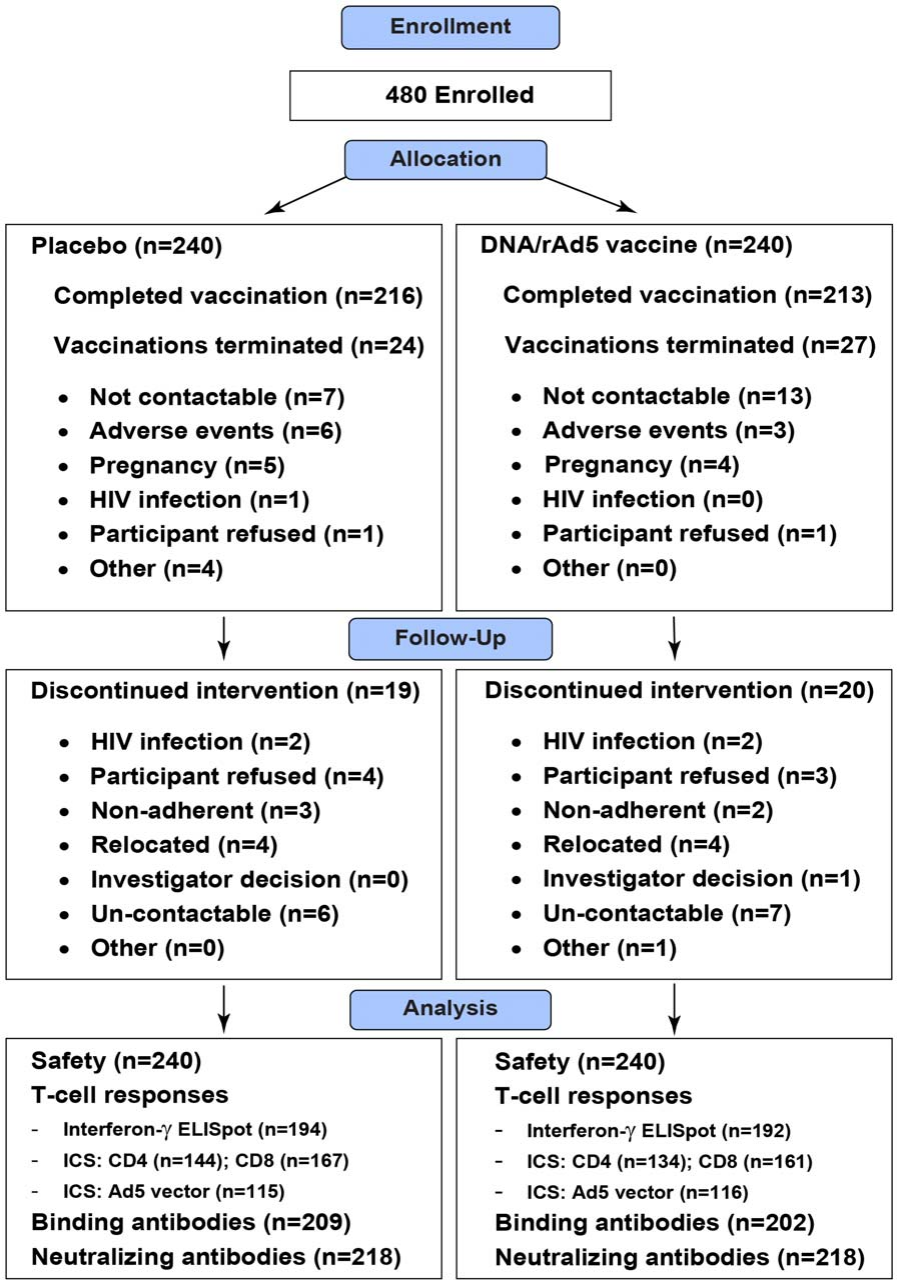
Number of individuals enrolled, randomized to vaccine or placebo, followed-up and analyzed. ICS = Intracellular cytokine staining, Ad5 = Adenovirus serotype 5.

The number of individuals enrolled and randomized to vaccine or placebo, followed-up and analyzed are shown in **Figure 1**. Between July 2006 and December 2007, 480 participants were enrolled and followed up: 240 were enrolled in South Africa and 240 were enrolled in the Americas (n = 180 in the United States and n = 60 in Latin America and Caribbean [Jamaica, Haiti and Brazil]). Of the 240 vaccine and placebo recipients, 206 (85.8%) and 207 (86.3%) participants completed all vaccinations and study visits. The baseline characteristics of the cohort are summarized in **Table 1**. The vaccine and placebo arms were similar with respect to geographic region, age, gender, ethnic group, sexual orientation, pre-existing Ad5 neutralizing antibodies and the percent completing vaccinations and all study visits.

**Table 1:** Cohort characteristics.

		Placebo (N = 240)	Vaccine (N = 240)
**Geographic region**	United States	90 (37.5%)	90 (37.5%)
	South Africa	120 (50%)	120 (50%)
	Latin America & Caribbean	30 (12.5%)	30 (12.5%)
**Median age (range) (yrs)**		26.0 (18–50)	25.0 (18–50)
**Female (%)**		127 (53%)	127 (53%)
**Ethnic group (%)**	White	58 (24%)	58 (24%)
	Black	161 (67%)	162 (68%)
	Hispanic	16 (7%)	14 (6%)
	Other	5 (2%)	11 (5%)
**Sexual orientation**	Heterosexual	210 (88%)	212 (88%)
	Homosexual	20 (8%)	22 (9%)
	Bisexual	10 (4%)	6 (3%)
**Pre-existing Ad5 Nab titer** *	**<12**		
	United States	24 (27%)	38 (42%)
	South Africa	8 (7%)	4 (3%)
	Latin America & Caribbean	3 (10%)	4 (13%)
	**>12**		
	United States	66 (73%)	52 (58%)
	South Africa	105 (88%)	109 (91%)
	Latin America & Caribbean	27 (90%)	26 (87%)
	**Missing**		
	United States	0 (0%)	0 (0%)
	South Africa	7 (6%)	7 (6%)
	Latin America & Caribbean	0 (0%)	0 (0%)
**Number vaccinated**	Month 0 (DNA/placebo)	240 (100%)	240 (100%)
	Month 1 (DNA/placebo)	234 (98%)	231 (96%)
	Month 2 (DNA/placebo)	228 (95%)	224 (93%)
	Month 6 (Ad5/placebo)	216 (90%)	213 (89%)
**HIV infections**		4 (1.7%)	3 (1.3%)

Latin America & Caribbean (Brazil, Jamaica, Haiti),

*Expressed as the proportion for each region.

### Safety

**Figure 2:**
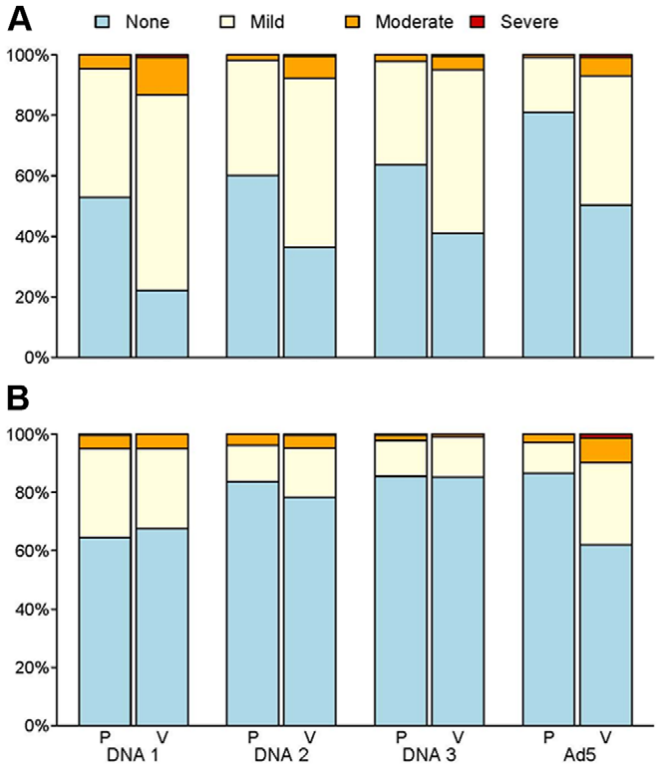
Maximal local and systemic reactogenicity after each vaccination. Frequency of local (A) and systemic (B) reactions occurring within three days following each DNA and rAd5 vaccination. Local injection site reactions included pain, tenderness, erythema and induration. Systemic reactions included malaise/fatigue, headache, chills, myalgia, arthralgia or increased body temperature. P = Placebo, V = Vaccine.

Injection site reactions (≥mild) were more common in DNA-HIV and rAd5-HIV vaccine recipients than placebo recipients (DNA-HIV: 86.3% vs 67.5%, p<0.001; Ad5-HIV: 49.8% vs 19.0%, p<0.001) (**Figure 2A**). Local reactions decreased with each subsequent dose of DNA or placebo (p<0.0001) with no effect modification by treatment (p = 0.06). Maximal systemic symptoms and signs (≥mild) did not differ between DNA-HIV and placebo recipients overall (p = 0.582) but were significantly more common in rAd5-HIV vaccine than placebo recipients (38.0% vs 13.4%, p<0.001) (**Figure 2B**). Systemic reactions also decreased with each subsequent dose of DNA or placebo (p<0.0001) with no effect modification by treatment (p = 0.40).

Detailed monitoring showed that the number of DNA-HIV and rAd5-HIV vaccine recipients that had one or more adverse events was similar to that of placebo recipients. Signs and symptoms of upper respiratory tract infections and headache were the most frequent adverse events in DNA-HIV recipients (**Table 2**). There were no significant alterations in liver function tests and white blood cell counts in rAd5-HIV vaccine compared to placebo recipients (**Table 2**). Of the 9 pregnancies that occurred in the placebo arm, there were two elective and one spontaneous abortion and six healthy infants born. Of the 7 pregnancies in the vaccine arm, there were four elective abortions, 2 healthy infants born, and the outcome of one pregnancy was unknown. Four placebo and three vaccine recipients became HIV-infected during the 12 month study follow up period, all in the South African cohort. One vaccinee and two placebo recipients were found to be HIV positive after terminating the study based on a sample collected at the Month 12 study visit. The vaccine commonly produced antibodies to HIV proteins after the rAd5 boost; 87.9% ([204/232], 95% CI 83.1%–91.5%) of recipients had a false positive HIV-1 test to at least one of the diagnostic HIV ELISAs used.

**Table 2:** Adverse events occurring more frequently in vaccine participants.

DNA vaccine or placebo.	Vaccine (n = 240)		Placebo (n = 240)	
	N	(%)	N	(%)
**Total with one or more adverse events**	**177**	**(73.8%)**	**172**	**(71.7%)**
Upper respiratory tract infection	38	(15.8%)	31	(12.9%)
Headache	16	(6.7%)	14	(5.8%)
Nasopharyngitis	14	(5.8%)	9	(3.8%)
Neutrophil count decreased	9	(3.8%)	3	(1.3%)
Lymphadenopathy	8	(3.3%)	3	(1.3%)
Pharyngitis	8	(3.3%)	2	(0.8%)
Proteinuria	8	(3.3%)	3	(1.3%)
Haematuria	7	(2.9%)	6	(2.5%)
Diarrhoea	5	(2.1%)	4	(1.7%)
Injection site pruritus	5	(2.1%)	0	(0.0%)
Toothache	5	(2.1%)	3	(1.3%)
Anaemia	4	(1.7%)	0	(0.0%)
Blood pressure increased	4	(1.7%)	3	(1.3%)
Cough	4	(1.7%)	1	(0.4%)
Dermatitis contact	4	(1.7%)	1	(0.4%)
Epistaxis	4	(1.7%)	2	(0.8%)
Influenza	4	(1.7%)	1	(0.4%)
Injection site swelling	4	(1.7%)	1	(0.4%)
Weight decreased	4	(1.7%)	2	(0.8%)
Dermatitis	3	(1.3%)	2	(0.8%)
Excoriation	3	(1.3%)	0	(0.0%)
Furuncle	3	(1.3%)	0	(0.0%)
Lower respiratory tract infection	3	(1.3%)	1	(0.4%)
Menorrhagia	3	(1.3%)	0	(0.0%)
Sexually transmitted disease	3	(1.3%)	0	(0.0%)
Stomach discomfort	3	(1.3%)	0	(0.0%)
Tooth abscess	3	(1.3%)	2	(0.8%)
Urticaria	3	(1.3%)	1	(0.4%)
Vomiting	3	(1.3%)	2	(0.8%)

rAd5 vaccine or placebo	Vaccine (n = 213)		Placebo (n = 216)	
**Total with one or more adverse events**	**148**	**(69.5%)**	**151**	**(69.9%)**
Alanine aminotransferase increased	17	(8.0%)	10	(4.6%)
Nasopharyngitis	11	(5.2%)	11	(5.1%)
Weight decreased	9	(4.2%)	2	(0.9%)
Neutropenia	7	(3.3%)	6	(2.8%)
Neutrophil count decreased	6	(2.8%)	5	(2.3%)
Anaemia	5	(2.3%)	4	(1.9%)
Vaginal candidiasis	5	(2.3%)	4	(1.9%)
Pain in extremity	4	(1.9%)	1	(0.5%)
Viral infection	4	(1.9%)	2	(0.9%)
Abdominal pain lower	3	(1.4%)	0	(0.0%)
Diarrhoea	3	(1.4%)	1	(0.5%)
Otitis media	3	(1.4%)	0	(0.0%)
Sinusitis	3	(1.4%)	2	(0.9%)
Subcutaneous abscess	3	(1.4%)	1	(0.5%)
Vomiting	3	(1.4%)	0	(0.0%)

### T-cell responses detected by IFN-γ ELISpot

**Figure 3:**
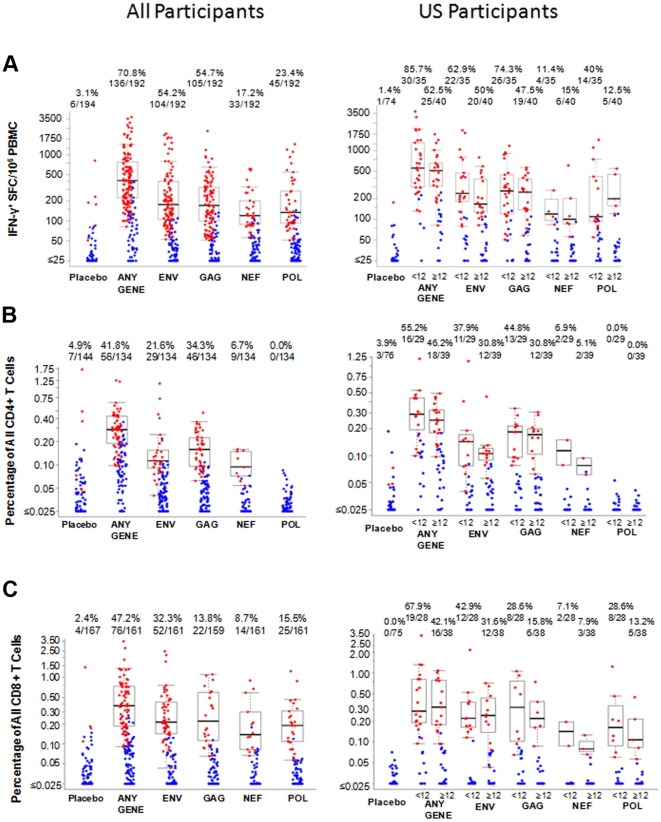
Magnitude of T-cell responses recognizing global PTEs 6 weeks after the Ad5 boost in all participants and by Ad5 titer in US participants. Shown are the magnitude of T-cell responses as measured by IFN-γ ELISpot responses (A) and percentage of CD4+ (B) and CD8+ (C) T-cells producing interferon-γ and/or interleukin-2 in response to Env, Gag, Pol or Nef six weeks after rAd5 boost in all participants (left) and in United States participants stratified by adenovirus serotype-5 titer (<12, ≥12, right). Only the Ad5 data for US participants is shown as they had the highest proportion of participants who were Ad5 negative at baseline. Positive responders are shown in red and negative responders in blue. The boxplots show the distribution of responses in positive responders only. The box indicates the median and inter-quartile range; whiskers extend to 1.5 times the inter-quartile range from the upper or lower quartile. The numbers above the graphs indicate the number positive relative to the total number examined and the corresponding percentage positive.

Six weeks after the rAd5-HIV boost, the percent of participants with detectable T-cell responses by IFN-γ ELISpot, using global PTE peptides, to any antigen among placebo and vaccine recipients was 3.1% (6/194) and 70.8% (136/192), respectively (p<0.01) (**Table 3**). Responses were observed for all antigens, but most frequently to Gag (54.7%) and Env (54.2%), and did not differ by geographical region (**Table 3**). Among the 136 vaccine recipients with positive responses, 30.9%, 33.8%, 28.7% and 6.6% had responses to 1, 2, 3 and 4 genes, respectively, with the most frequent combination of responses to Env/Gag (23.5%), Env/Gag/Pol (19.1%), Env only (14.0%) and Gag only (14.0%). The median magnitude of response (SFC/10^6^ PBMC) were: any antigen 408; Env 177; Gag 171; Nef 120 and Pol 134 (**Figure 3**).

**Table 3:** Frequency of interferon-γ T cell ELISpot, intracellular cytokine staining, binding and neutralizing antibody and Ad5 vector responses.

			Placebo			Vaccine	
		N	%	95% CI	N	%	95% CI
**IFN-γ ELISpot** *	Overall	194	3.1	1.4–6.6	192	70.8	64.0–76.8
	**Region**						
	LAC	23	4.3	0.8–21.0	24	70.8	50.8–85.1
	USA	74	1.4	0.2–7.3	75	73.3	62.4–82.0
	South Africa	97	4.1	1.6–10.1	93	68.8	58.8–77.3
	**Antigen**						
	Env	194	2.1	0.8–5.2	192	54.2	47.1–61.1
	Gag	194	0.5	0.1–2.9	192	54.7	47.6–61.6
	Nef	194	0.0	0.0–1.9	192	17.2	12.5–23.2
	Pol	194	0.5	0.1–2.9	192	23.4	18.0–29.9
**Intracellular Cytokine Staining** *	**CD4**						
	Overall	144	4.9	2.4–9.7	134	41.8	33.8–50.3
	Env	144	3.5	1.5–7.9	134	21.6	15.5–29.4
	Gag	144	1.4	0.4–4.9	134	34.3	26.8–42.7
	Nef	144	0.0	0.0–2.6	134	6.7	3.6–12.3
	Pol	144	0.0	0.0–2.6	134	0.0	0.0–2.8
	**CD8**						
	Overall	167	2.4	0.9–6.0	161	47.2	39.6–54.9
	Env	167	1.8	0.6–5.1	161	32.3	25.6–39.9
	Gag	165	0.6	0.1–3.4	159	13.8	9.3–20.1
	Nef	167	0.0	0.0–2.2	161	8.7	5.3–14.1
	Pol	167	0.0	0.0–2.2	161	15.5	10.7–21.9
	**Ad5 vector responses** *						
	Any	115	51.3	42.3–60.2	116	75.0	66.4–82.0
	CD4	92	62.0	51.7–71.2	92	83.7	74.8–89.9
	CD8	115	13.9	8.7–21.4	116	37.9	29.6–47.0
**Binding antibody responses** **	gp140 Con S	209	1.0	0.3–3.4	202	94.6	90.5–96.9
	gp140 Clade C	209	0	0.0–1.8	202	93.1	88.7–95.8
	gp140 Clade A	209	0	0.0–1.8	202	83.7	77.9–88.1
	gp140Clade B	209	0	0.0–1.8	202	94.6	90.5–96.9
	gp41	209	0.5	0.1–2.7	202	93.1	88.7–95.8
	P55	209	1.0	0.3–3.4	202	45.5	38.8–52.4
**Neutralizing antibody responses** **	92RW020.2	218	0.0	0.0–1.7	220	0.0	0.0–1.7
	97ZA012.29	218	0.0	0.0–1.7	220	0.0	0.0–1.7
	Bal.26	218	0.0	0.0–1.7	220	0.9	0.2–3.3
	MN	218	5.0	2.8–8.8	220	18.6	14.0–24.3
	SF162.LS	218	0.0	0.0–1.7	220	7.7	4.9–12.0

All responses are to Global PTE peptide pools.

*6 weeks after Ad5 boost,

**4 weeks after rAd5 boost. USA = United States of America. LAC = Latin America and Caribbean. N's refer to number of samples with evaluable data (that passed all quality control filters). ELISpot assays were run on 209 placebos (P) and 205 vaccinees (V): Latin America: 24 P, 24 V; USA: 81 P, 80 V; South Africa: 104 P, 101 V. CD4 and CD8 ICS assays were run on 175 P and 167 V; Ad5 vector ICS assays were run on 115 P and 116 V. Neutralizing antibody assays were run on 220 P and 222 V. Luminex binding antibody assays were run on 219 P and 206 V.

In contrast to Ad5-seronegative subjects, a smaller proportion of U.S. subjects who had Ad5 neutralizing antibodies (Ad5 NAb) at baseline had positive T-cell responses for any antigen (62.5% vs. 85.7%, p = 0.035), Env (50% vs. 62.9%, p = 0.352), Gag (47.5% vs. 74.3%, p = 0.021) and Pol (12.5% vs. 40.0%, p = 0.008); the placebo response rate was 1.4% (1/74) overall. Not unexpectedly, responses to Nef were similar (15.0% vs. 11.4%, p = 0.742) as Nef was not included in the rAd5-HIV vaccine (**Table 4**). The median magnitude (SFC/10^6^ PBMC) of responses were slightly lower among Ad5 seropositive versus seronegative participants for any antigen (513 versus 560), Env (167 versus 239), Gag (250 versus 259) and Nef (100 versus 120) but was higher for Pol (200 versus 110) (**Figure 3**).

**Table 4:** Frequency of ELISpot, CD4 and CD8 T-cell ICS responses 6 weeks after Ad5 boost among US participants, stratified by pre-existing Ad5 NAb titer.

	Pre-existing Ad5 nAb titer <12				Pre-existing Ad5 nAb titer ≥12			
	Placebo		Vaccine		Placebo		Vaccine	
	%	95%CI	%	95%CI	%	95%CI	%	95%CI
**IFN-γ ELISpot**								
	**N = 20**		**N = 35**		**N = 54**		**N = 40**	
Overall	5.0%	0.9%–23.6%	85.7%	70.6%–93.7%	0.0%	0.0%–6.6%	62.5%	47.0%–75.8%
**Antigen**								
Gag	0.0%	0.0%–16.1%	74.3%	57.9%–85.8%	0.0%	0.0%–6.6%	47.5%	32.9%–62.5%
Pol	5.0%	0.9%–23.6%	40.0%	25.6%–56.4%	0.0%	0.0%–6.6%	12.5%	5.5%–26.1%
Nef	0.0%	0.0%–16.1%	11.4%	4.5%–26.0%	0.0%	0.0%–6.6%	15.0%	7.1%–29.1%
Env	0.0%	0.0%–16.1%	62.9%	46.3%–76.8%	0.0%	0.0%–6.6%	50.0%	35.2%–64.8%
**Intracellular Cytokine Staining**								
**CD4+**	**N = 21**		**N = 29**		**N = 55**		**N = 39**	
Overall	0.0%	0.0%–15.5%	55.2%	37.5%–71.6%	5.5%	1.9%–14.9%	46.2%	31.6%–61.4%
**Antigen**								
Gag	0.0%	0.0%–15.5%	44.8%	28.4%–62.5%	3.6%	1.0%–12.3%	30.8%	18.6%–46.4%
Pol	0.0%	0.0%–15.5%	0.0%	0.0%–11.7%	0.0%	0.0%–6.5%	0.0%	0.0%–9.0%
Nef	0.0%	0.0%–15.5%	6.9%	1.9%–22.0%	0.0%	0.0%–6.5%	5.1%	1.4%–16.9%
Env	0.0%	0.0%–15.5%	37.9%	22.7%–56.0%	1.8%	0.3%–9.6%	30.8%	18.6%–46.4%
**CD8+**	**N = 21**		**N = 28**		**N = 54**		**N = 38**	
Overall	0.0%	0.0%–15.5%	67.9%	49.3%–82.1%	0.0%	0.0%–6.6%	42.1%	27.9%–57.8%
**Antigen**								
Gag	0.0%	0.0%–15.5%	28.6%	15.3%–47.1%	0.0%	0.0%–6.6%	15.8%	7.4%–30.4%
Pol	0.0%	0.0%–15.5%	28.6%	15.3%–47.1%	0.0%	0.0%–6.6%	13.2%	5.8%–27.3%
Nef	0.0%	0.0%–15.5%	7.1%	2.0%–22.6%	0.0%	0.0%–6.6%	7.9%	2.7%–20.8%
Env	0.0%	0.0%–15.5%	42.9%	26.5%–60.9%	0.0%	0.0%–6.6%	31.6%	19.1%–47.5%
**Ad5 vector** *	**N = 12**		**N = 22** **		**N = 37** **		**N = 24** **	
Overall	91.7%	64.6%–98.5%	95.5%	78.2%–99.2%	83.8%	68.9%–92.3%	87.5%	69.0%–95.7%
CD4+	91.7%	64.6%–98.5%	90.5%	71.1%–97.3%	86.1%	71.3%–93.9%	87.0%	67.9%–95.5%
CD8+	25.0%	8.9%–53.2%	40.9%	23.3%–61.3%	24.3%	13.4%–40.1%	54.2%	35.1%–72.1%

*Overall CD4+ and CD8+ T cell ICS responses to the empty Ad5 vector,

**Data missing for one participant for CD4+ T-cell responses.

### Intracellular cytokine staining responses

#### CD4+ and CD8+ T-cell IFN-γ and/or IL2 responses

A similar frequency of vaccine recipients had HIV-specific CD4+ (56/134 = 41.8%) and CD8+ (76/161 = 47.2%) T-cell responses producing IL-2 and/or IFN-γ in response to at least one peptide pool (**Table 3**). Among 130 vaccine recipients with concurrent CD4+ and CD8+ T-cell responses to at least one antigen, 16.9% (22/130) had CD4+ and CD8+ T-cell responses to the same antigen, that is Env, Gag, Nef or Pol. The distribution of responses to individual antigens was however different for each T-cell: the highest frequency of CD4+ responses were to Gag (34.3%) and Env (21.6%) and there were no responses to Pol; the highest frequency CD8+ T-cell responses were seen to Env (32.3%) and Pol (15.5%). U.S. vaccine recipients who were Ad5 seropositive versus seronegative had lower response rates, particularly for CD8+ T cells (CD4: 46.2% vs. 55.2%, p = 0.624; CD8: 42.1% vs. 67.9%, p = 0.048), which was consistent for all antigens (**Table 4**). The median magnitude of CD4+ and CD8+ T-cell responses were similar (**Figure 3**) but the antigen specificity differed by T-cell type: the greatest median magnitude of CD4+ T-cell response was to Gag; the median magnitude of CD8+ T-cell responses were similar for all antigens. Among Ad5 seropositive versus seronegative U.S. participants the median magnitude of CD4+ responses (percent positive) were slightly lower for all antigens (Env: 0.11 vs. 0.14; Gag: 0.17 vs. 0.18; Nef: 0.08 vs. 0.11). Similarly the median magnitude of CD8+ T-cells responses were lower in seropositive U.S. participants (Gag: 0.22 vs.0.32; Nef: 0.08 vs. 0.14; Pol: 0.11 vs. 0.16) apart from Env (0.22 and 0.24) (**Figure 3**).

#### Polyfunctionality of CD4+ and CD8+ T-cells

**Figure 4:**
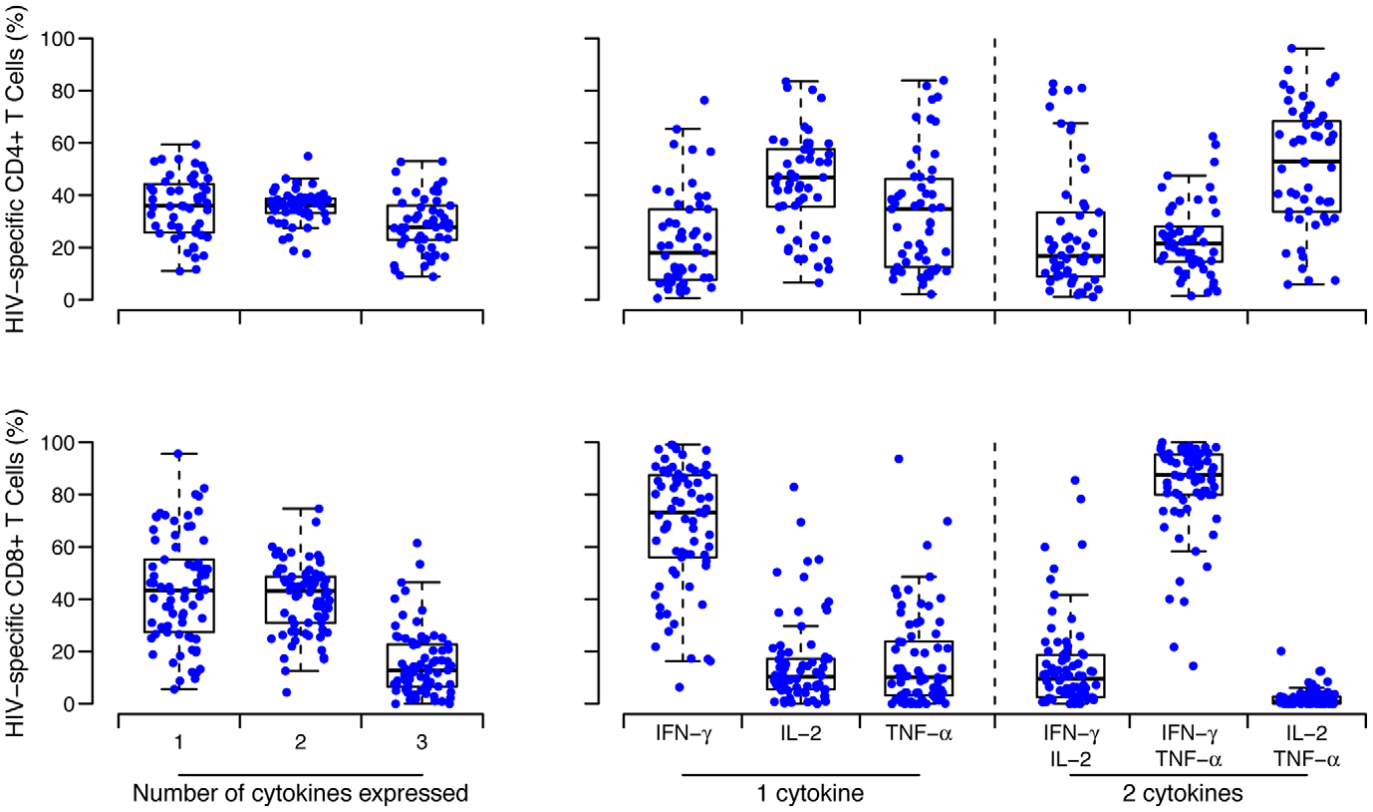
Polyfunctional CD4+ and CD8+ T-cell IFN-γ, TNF-α and IL2 cytokine responses. The left graphs show the percentage of the HIV-specific CD4+ (upper panel) or CD8+ (lower panel) T-cells that are producing one, two or three cytokines in the vaccine recipients. Intracellular cytokine staining analyses were done on PBMC obtained six weeks after the rAd5 boost. The right graphs depict the percentage of cells producing interferon-γ, interleukin-2 or tumor necrosis factor-α in those cells producing one cytokine (middle panels), and the percentage of cells co-producing two cytokines (panels on the right). The boxplots show the distribution of responses in positive responders only. The box indicated the median and inter-quartile range; whiskers extend to 1.5 times the inter-quartile range from the upper or lower quartile.

The proportion of vaccine recipients with CD4+ and CD8+ reactive T-cells to PTE peptides that produced one or more cytokines (IFN-γ, IL-2, TNF-α) are shown in **Figure 4**. The proportion who had CD4+ T-cells producing one, two or three cytokines was similar. Among single and double-cytokine-producing CD4+ responsive T-cells, IL-2 and IL-2/TNF-α were produced most often. Most CD8+ reactive T-cells produced only one or two cytokines. Among single and double-cytokine-producing CD8+ T-cells, IFN-γ and IFN-γ/TNF-α were produced most often. Among U.S. participants, the proportion of CD4+ and CD8+ responsive T-cells that produced one or more cytokine did not differ by Ad5 Nab status (data not shown).

#### Ad5 vector T-cell responses

**Figure 5:**
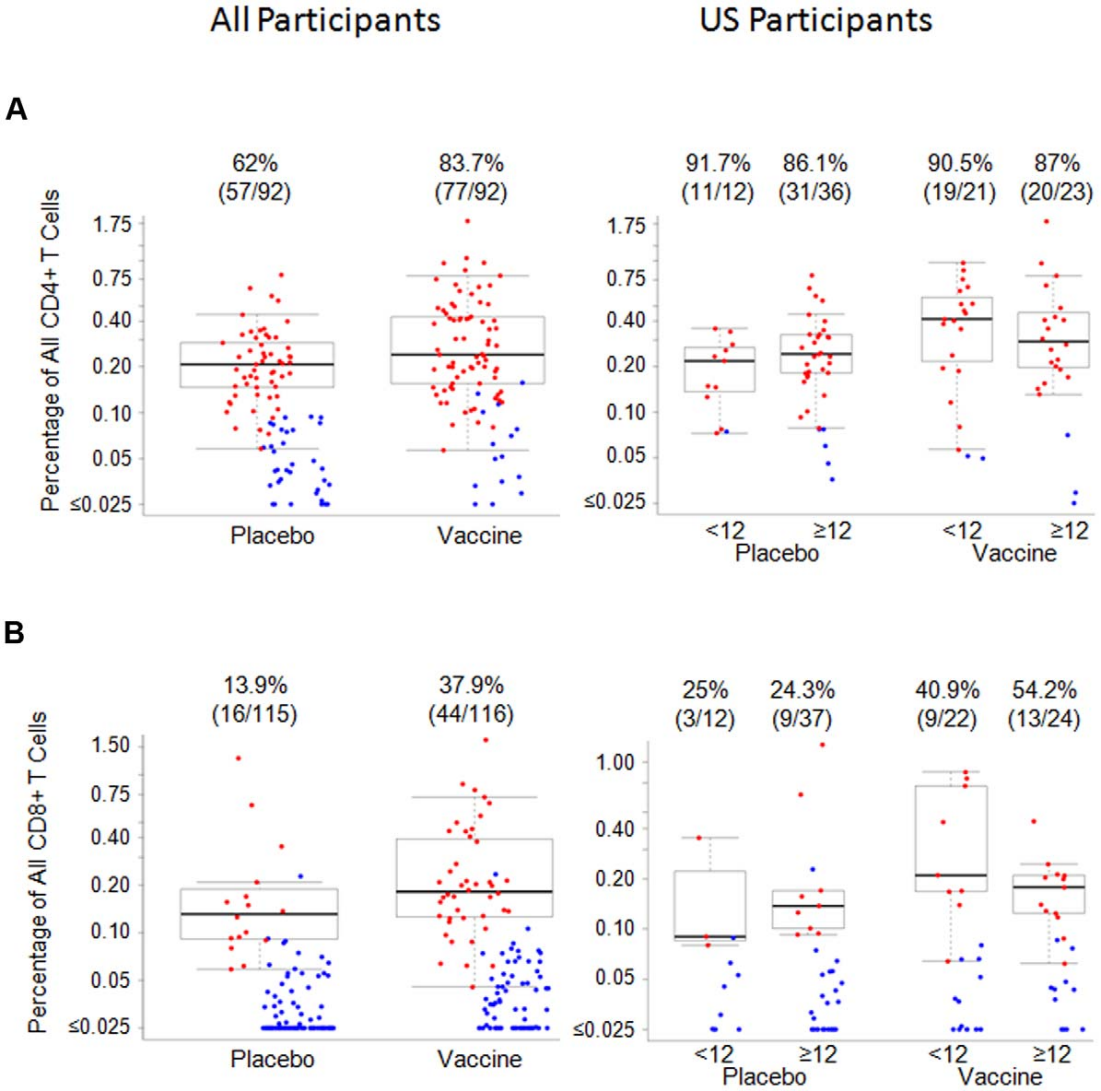
Magnitude of CD4+ and CD8+ T cell ICS responses to empty Ad5 vector. Percentage of CD4+ (top panels) and CD8+ T cells (bottom panels) producing interferon-γ and/or interleukin 2, or both, by intracellular cytokine staining, in response to Ad5 empty vector stimulation in all (left panels) or US only (right panels) placebo and vaccine recipients 6 weeks after the Ad5 boost. Positive responders are shown in red and negative responders in blue. The box plots and numbers above the graphs are as in Figure 2.

IL-2 and/or IFN-γ ICS responses six weeks after the Ad5 boost to the empty Ad5 vector were significantly higher in vaccine (n = 116) compared to placebo (n = 115) recipients overall (75% versus 51.3%, p<0.001) (**Table 3**). A significantly greater proportion of CD4+ compared to CD8+ reactive T cells responded to the empty Ad5 vector in vaccine (83.7% versus 37.9%, respectively, p<0.0001) and placebo (62.0% versus 13.9%, respectively, p<0.0001) recipients. The magnitude of reactive CD4+ and CD8+ T-cell responses to the empty rAd5 vector were similar in vaccine and placebo recipients (**Figure 5**). Among vaccine and placebo recipients enrolled in the U.S. the frequency and magnitude of responses to the empty rAd5 vector were similar in Ad5 seronegative and seropositive participants (**Table 4**, **Figure 5**), indicating that the Ad5-specific T-cell responses were influenced by previous exposure to other adenovirus serotypes and cross reactive responses to these serotypes.

### Binding and neutralizing antibody responses

A very high frequency of binding antibody responses were observed to Con S (94.6%), Clade A (83.7%), Clade B (94.6%) and Clade C (93.1%) gp140 oligomers and to gp41 (93.1%); and moderate responses to p55 (45.5%) (**Table 3**).

**Figure 6:**
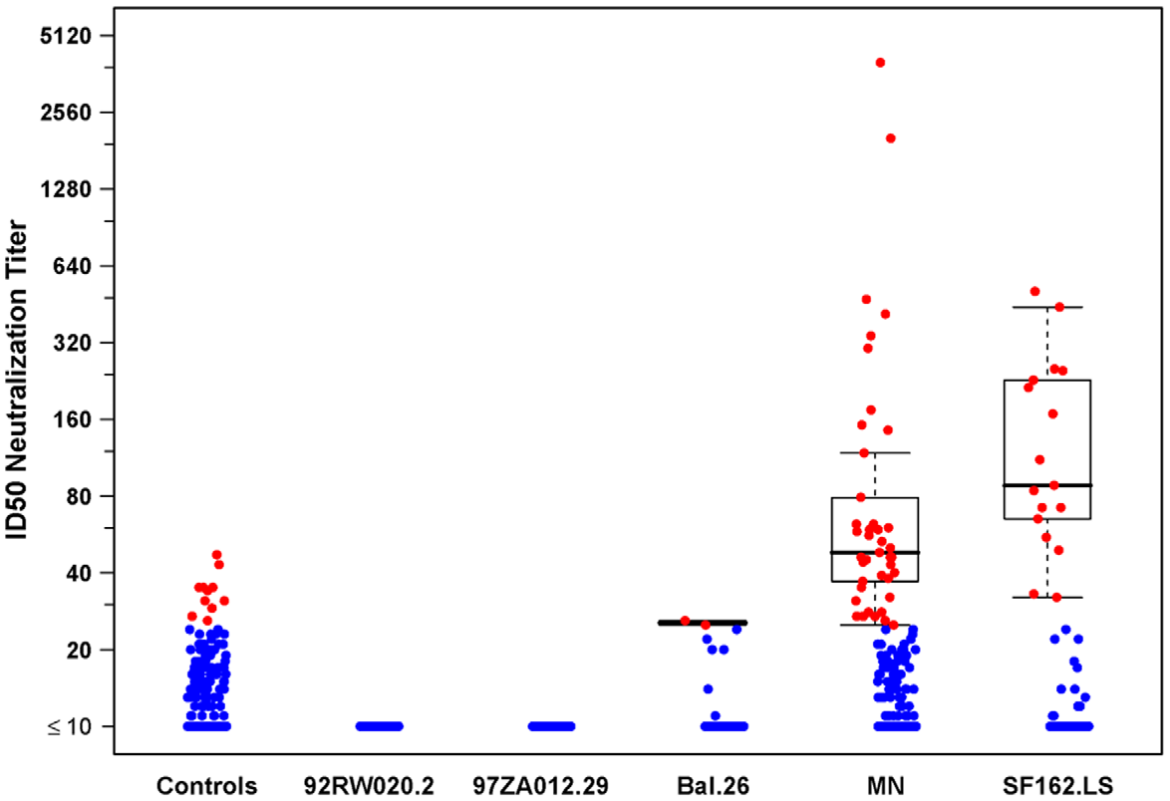
Neutralizing antibody responses to Tier 1 viruses. Positive responders are shown in red and negative responders in blue. The boxplots show the distribution of responses in positive responders only. The box indicates the median and inter-quartile range; whiskers extend to 1.5 times the inter-quartile range from the upper or lower quartile.

Neutralizing antibody (NAb) assays were performed for 218 and 220 placebo and vaccine recipients, respectively. There were no Nab responses detected to 92RW020.2 and 97ZA012.29 isolates. The greatest frequency of responses was to MN (18.6%), SF162.LS (7.7%) and Bal.26 (0.9%) Tier 1 viruses (**Table 3**). Despite potent neutralizing activity against MN and SF162.LS (**Figure 6**) very little neutralizing activity was detected against tier 2 viruses (data not shown).

## Discussion

This study of the VRC multigene, multiclade DNA-HIV prime, rAd5-HIV boost vaccine regimen showed that the vaccines were well tolerated, safe and immunogenic. T-cell immunogenicity of the vaccine regimen, as measured by the IFN-γ ELISpot assay, was consistent with those reported from the IAVI V001 trial in Kenya and Rwanda and the URMHRP RV172 trial in Kenya, Tanzania and Uganda [11], [12]. In addition, our study provides a more in-depth look at HIV-specific immune responses using the ICS assay, as well as contrasting these data with that directed towards the rAd5 vector itself.

The results from two recent preventive HIV vaccine efficacy trials provide important insights into what may be required to develop an effective HIV-1 vaccine. The RV144 trial in Thailand showed modest efficacy of the recombinant canarypox vector vaccine (ALVAC-HIV) prime, which expresses subtype E gp120, and the ALVAC-HIV and AIDSVAX subtype B/E gp120 subunit vaccine boost in a cohort that was at relatively low-risk for heterosexual HIV acquisition. That study has highlighted the potential importance of inducing Env-specific antibodies in the protection against HIV infection [2], [23], [24], even those lacking classical neutralizing activity. The extent to which having both Env and internal antigens in the vaccine preparation, the role of HIV-specific CD4+ versus CD8+ T-cell responses and a study population with a relatively low HIV incidence is the subject of ongoing investigation. In contrast, the phase IIB study of the replication-defective Merck rAd5-vectored vaccine (Merck 023/HVTN 502; the STEP Study) composed of clade B Gag, Nef and Pol (without Env) and given at 0, 1 and 6 months to high-risk men who have sex with men and women was stopped prematurely when a pre-defined futility criteria was met and there was no evidence that the vaccine provided protection against HIV infection or reduced viral load among infected participants, despite frequent and strong CD8+ T-cell responses to vaccine specific antigens [13].

Nevertheless, in sub-studies evaluating subjects with selected HLA types [25] and in an intensive sequence analysis of breakthrough HIV isolates [26] there was evidence of a CD8 T-cell effect. Subjects with HLA-B27, HLA-B57, or HLA-B5801 who were vaccinated had significantly lower viral loads than placebo recipients bearing the same alleles [25]. Similarly, comparing breakthrough viruses from vaccinees and placebo recipients there was a conspicuous signature sequence in Gag aa84 that involves a number of overlapping MHC class I-restricted epitopes [26].

Similar to the RV144 ALVAC/AIDSVAX HIV vaccine regimen the VRC DNA-HIV prime, rAd5-HIV boost regimen included Env and induced Env-specific binding antibody responses to the vaccine strain antigens [24]. Binding antibody responses to the VRC vaccine strain envelopes were of similar or higher magnitude compared to the vaccine elicited antibody responses in RV144. However, additional assays will be needed to determine whether there are differences in the quality of the antibody response, in terms of antibody specificity, avidity and functional anti-viral properties of the vaccine elicited responses in HVTN 204 compared to RV144. If antibody correlates of protection are identified in the future analysis of RV144, these assays could be applied to samples from this study.

The vaccine regimen evaluated in this study induced comparable CD8 T-cell response magnitude and quality compared to the responses induced by the Merck rAd5-HIV vaccine. However, there are differences in antigen content, rAd5 vector construction, and vaccine regimen that may influence the specificity and breadth of the immune response. The VRC rAd5 expresses Env antigens in addition to Gag and Pol and has deletions in E3, E4 as well as E1 adenovirus genes that prevent production of adenovirus structural proteins, thus focusing the antigen presentation machinery on the vaccine antigen instead of the vector. In addition, compared to the vaccine studied in the RV144 trial, the VRC regimen induces a greater frequency and magnitude of HIV-specific CD8+ T-cell responses [13], [24], [27].

South African participants in the HVTN204 trial had a very high prevalence of pre-existing Ad5 Nabs titers (89%), which is similar to other studies from Central and East Africa [11], [12]. In contrast, a far lower proportion of US participants were Ad5 sero-positive (66%) at baseline, which is in keeping with other studies [28]. Neither the USMHRP RV172 nor IAVI V001 trials showed that the frequency of T-cell responses in African participants vaccinated with the VRC DNA-HIV prime/rAd5-HV boosts differed by baseline Ad5 NAb status [11], [12]. In the RV172 trial there was a non-significant trend to lower magnitude of IFN-γ ELISPOT responses in participants with higher baseline Ad5 titers. In contrast to RV172 and V001, the HVTN204 trial provides strong evidence that pre-existing Ad5 NAb results in a modest reduction in the frequency of IFN-γ ELISPOT, CD4+ and CD8+ T-cell ICS responses to Env, Gag and Pol but not Nef, which was not included in the rAd5 boost. Of interest, the frequency of CD4+ T-cell responses to the Ad5 vector were twice that of the responses to the HIV gene inserts, whereas CD8+ T-cell responses to the Ad5 vector and HIV gene inserts were similar. The biological basis for this finding is not clear.

Thus, because pre-existing immunity to Ad5 in the developing world is moderately high, strategies to overcome pre-existing neutralizing antibodies from natural Ad5 infection are being explored. Natural infection with Ad5 and rAd5 vaccination induce different patterns of neutralizing antibody response, with natural infection eliciting both fiber and capsid-specific responses and vaccination eliciting more exclusively capsid-specific responses [29]. Use of chimeric adenovirus vectors or rare serotype adenovirus vectors, may avoid the impact of pre-existing immunity to the vector.

The results from this and other studies have shown that the VRC multi-clade DNA-HIV prime, rAd5-HIV boost regimen is safe and, like the RV144 ALVAC/AIDSVAX HIV vaccine regimen, induces a high frequency of Env-specific binding antibodies [8], [27] with poor and low levels of neutralizing activity. The VRC vaccine regimen also induces: durable, concurrent, polyfunctional CD4+ and CD8+ T-cell responses specific for multiple HIV antigens [8], [12]; and central memory and effector CD8+ T cell responses with antiviral activity, which may play a role in preventing infection and controlling chronic infection [12], [30], [31]. However, antigen specific T-cell responses are reduced in Ad5 Nab seropositive individuals. A limitation of this study is that the effect of pre-existing Ad5 Nabs on HIV-specific T-cell responses was not determined for all participants. These data were important for supporting the initiation of a test of concept study of the DNA-HIV prime, rAd5-HIV boost regimen in HVTN 505, but because of the findings of the STEP Study, the study population was limited to circumcised, Ad5 seronegative men in the U.S. Recently, based on the immunogenicity data from HVTN 204 and the RV144 studies and recent findings showing that non-human primates immunized with the DNA/rAd5 platform were protected from repeated mucosal SIV challenge [32], HIV acquisition has been added as a primary endpoint in the ongoing HVTN 505 study.

## Supporting Information

Protocol S1 **Trial Protocol.**
(PDF)

Checklist S1 **CONSORT Checklist.**
(DOC)

## References

[1] Joint United Nations Programme on HIV/AIDS (2009). 2009 AIDS epidemic update.. http://data.unaids.org/pub/Report/2009/jc1700_epi_update_2009_en.pdf.

[2] Rerks-Ngarm S, Pitisuttithum P, Nitayaphan S, Kaewkungwal J, Chiu J (2009). Vaccination with ALVAC and AIDSVAX to prevent HIV-1 infection in Thailand.. N Engl J Med.

[3] Korber B, Gaschen B, Yusim K, Thakallapally R, Kesmir C (2001). Evolutionary and immunological implications of contemporary HIV-1 variation.. Br Med Bull.

[4] Catanzaro AT, Koup RA, Roederer M, Bailer RT, Enama ME (2006). Phase 1 safety and immunogenicity evaluation of a multiclade HIV-1 candidate vaccine delivered by a replication-defective recombinant adenovirus vector.. J Infect Dis.

[5] Catanzaro AT, Roederer M, Koup RA, Bailer RT, Enama ME (2007). Phase I clinical evaluation of a six-plasmid multiclade HIV-1 DNA candidate vaccine.. Vaccine.

[6] Eller MA, Eller LA, Opollo MS, Ouma BJ, Oballah PO (2007). Induction of HIV-specific functional immune responses by a multiclade HIV-1 DNA vaccine candidate in healthy Ugandans.. Vaccine.

[7] Graham BS, Koup RA, Roederer M, Bailer RT, Enama ME (2006). Phase 1 safety and immunogenicity evaluation of a multiclade HIV-1 DNA candidate vaccine.. J Infect Dis.

[8] Koup RA, Roederer M, Lamoreaux L, Fischer J, Novik L (2010). Priming immunization with DNA augments immunogenicity of recombinant adenoviral vectors for both HIV-1 specific antibody and T-cell responses.. PLOS One.

[9] Letvin NL, Mascola JR, Sun Y, Gorgone DA, Buzby AP (2006). Preserved CD4+ central memory T cells and survival in vaccinated SIV-challenged monkeys.. Science.

[10] Mattapallil JJ, Douek DC, Buckler-White A, Montefiori D, Letvin NL (2006). Vaccination preserves CD4 memory T cells during acute simian immunodeficiency virus challenge.. J Exp Med.

[11] Kibuuka H, Kimutai R, Maboko L, Sawe F, Schunk MS (2010). A phase 1/2 study of a multiclade HIV-1 DNA plasmid prime and recombinant adenovirus serotype 5 boost vaccine in HIV-Uninfected East Africans (RV 172).. J Infect Dis.

[12] Jaoko W, Karita E, Kayitenkore K, Omosa G, Allen S (2010). Safety and immunogenicity study of multiclade HIV-1 adenoviral vector vaccine alone or as boost following a multiclade HIV-1 DNA vaccine in Africa.. PLOS One PLoS One.

[13] McElrath MJ, De Rosa SC, Moodie Z, Dubey S, Kierstead L (2008). HIV-1 vaccine-induced immunity in the test-of-concept Step Study: a case-cohort analysis.. Lancet.

[14] Li F, Malhotra U, Gilbert PB, Hawkins NR, Duerr AC (2006). Peptide selection for human immunodeficiency virus type 1 CTL-based vaccine evaluation.. Vaccine.

[15] Bull M, Lee D, Stucky J, Chiu YL, Rubin A (2007). Defining blood processing parameters for optimal detection of cryopreserved antigen-specific responses for HIV vaccine trials.. J Immunol Methods.

[16] Horton H, Thomas EP, Stucky JA, Frank I, Moodie Z (2007). Optimization and validation of an 8-color intracellular cytokine staining (ICS) assay to quantify antigen-specific T cells induced by vaccination.. J Immunol Methods.

[17] Goepfert PA, Tomaras GD, Horton H, Montefiori D, Ferrari G (2007). Durable HIV-1 antibody and T-cell responses elicited by an adjuvanted multi-protein recombinant vaccine in uninfected human volunteers.. Vaccine.

[18] Tomaras GD, Yates NL, Liu P, Qin L, Fouda GG (2008). Initial B-cell responses to transmitted human immunodeficiency virus type 1: virion-binding immunoglobulin M (IgM) and IgG antibodies followed by plasma anti-gp41 antibodies with ineffective control of initial viremia.. J Virol.

[19] Mascola JR, Souza D, Gilbert P, Hahn BH, Haigwood NL (2005). Recommendations for the design and use of standard virus panels to assess neutralizing antibody responses elicited by candidate human immunodeficiency virus type 1 vaccines.. J Virol.

[20] Polonis VR, Brown BK, Rosa BA, Zolla-Pazner S, Dimitrov DS (2008). Recent advances in the characterization of HIV-1 neutralization assays for standardized evaluation of the antibody response to infection and vaccination.. Virology.

[21] Moodie Z, Price L, Gouttefangeas C, Mander A, Janetzki S (2010). Response definition criteria for ELISPOT assays revisited.. Cancer Immunol Immunother.

[22] Agresti A, Coull BA (1998). Approximate is better than exact for interval estimation of binomial parameters.. Am Stat.

[23] Flynn NM, Forthal DN, Harro CD, Judson FN, Mayer KH (2005). Placebo-controlled phase 3 trial of a recombinant glycoprotein 120 vaccine to prevent HIV-1 infection.. J Infect Dis.

[24] Pitisuttithum P, Gilbert P, Gurwith M, Heyward W, Martin M (2006). Randomized, double-blind, placebo-controlled efficacy trial of a bivalent recombinant glycoprotein 120 HIV-1 vaccine among injection drug users in Bangkok, Thailand.. J Infect Dis.

[25] Fitzgerald DW, Janes H, Robertson M, Coombs R, Frank I (2011). An Ad5-vectored HIV-1 vaccine elicits cell-mediated immunity but does not affect disease progression in HIV-1-infected male subjects: results from a randomized placebo-controlled trial (the Step study).. J Infect Dis.

[26] Rolland M, Tovanabutra S, Decamp AC, Frahm N, Gilbert PB (2011). Genetic impact of vaccination on breakthrough HIV-1 sequences from the STEP trial.. Nat Med.

[27] Seaman MS, Xu L, Beaudry K, Martin KL, Beddall MH (2005). Multiclade human immunodeficiency virus type 1 envelope immunogens elicit broad cellular and humoral immunity in rhesus monkeys.. J Virol.

[28] Mast TC, Kierstead L, Gupta SB, Nikas AA, Kallas EG (2010). International epidemiology of human pre-existing adenovirus (Ad) type-5, type-6, type-26 and type-36 neutralizing antibodies: correlates of high Ad5 titers and implications for potential HIV vaccine trials.. Vaccine.

[29] Cheng C, Gall JG, Nason M, King CR, Koup RA (2010). Differential specificity and immunogenicity of adenovirus type 5 neutralizing antibodies elicited by natural infection or immunization.. J Virol.

[30] Freel SA, Lamoreaux L, Chattopadhyay PK, Saunders K, Zarkowsky D (2010). Phenotypic and functional profile of HIV-inhibitory CD8 T cells elicited by natural infection and heterologous prime/boost vaccination.. J Virol.

[31] Spentzou A, Bergin P, Gill D, Cheeseman H, Ashraf A (2010). Viral inhibition assay: a CD8 T cell neutralization assay for use in clinical trials of HIV-1 vaccine candidates.. J Infect Dis.

[32] Letvin NL, Rao SS, Montefiori DC, Seaman MS, Sun Y (2011). Immune and Genetic Correlates of Vaccine Protection Against Mucosal Infection by SIV in Monkeys.. Sci Transl Med.

