# Supplementary material for: A Phase IIA Randomized Clinical Trial of a Multiclade HIV-1 DNA Prime Followed by a Multiclade rAd5 HIV-1 Vaccine Boost in Healthy Adults (HVTN204)
Source: PLoS One. 2011 Aug 3;6(8):e21225. doi: 10.1371/journal.pone.0021225 (PMC3152265; doi:10.1371/journal.pone.0021225)
Supplement: Protocol S1 — Trial Protocol. (PDF) [file pone.0021225.s001.pdf]

THIS PROTOCOL IS BEING PROVIDED IN SUPPORT OF THE MANUSCRIPT REPORTING RESULTS FROM THE HVTN 204 VACCINE TRIAL. FOR ANY OTHER USE, PLEASE CONTACT TEMPLATE@HVTN.ORG.

PROTOCOL
HVTN 204

A Phase II clinical trial to evaluate the safety and immunogenicity of a multiclade HIV-1 DNA plasmid vaccine, VRC-HIVDNA016-00-VP, followed by a multiclade recombinant adenoviral vector HIV-1 vaccine boost, VRC-HIVADV014-00-VP, in HIV-1 uninfected adult participants

FOR REVIEW ONLY

BB IND 12326 HELD BY DAIDS

CLINICAL TRIAL SPONSORED BY

Division of AIDS (DAIDS)
National Institute of Allergy and Infectious Diseases (NIAID)
National Institutes of Health (NIH)
Department of Health and Human Services (DHHS)
Bethesda, Maryland, USA

VACCINE PROVIDED BY

Dale and Betty Bumpers Vaccine Research Center (VRC), NIAID, NIH
Bethesda, Maryland, USA

May 24, 2005

204v1 FOR COPYING ONLY

This page is intentionally blank

FOR REVIEW ONLY

Brief contents

Detailed contents 4
Schema 8
Overview 9
Introduction 11
Ethical considerations 12
Foreword 13
HIV epidemic at HVTN clinical trials sites 15

Study products

1 Product background and rationale 18
2 Study product descriptions 25
3 Preclinical studies 28
4 Clinical studies 33
5 Summary 41

Study design

6 Study objectives 44
7 Study type, study population, and eligibility criteria 45
8 Safety and immunogenicity evaluations 48
9 Statistical considerations 51

Study operations

10 Protocol conduct 60
11 Informed consent 61
12 Procedures 63
13 Study product preparation and administration 65
14 Safety monitoring and review 69
References 75
Protocol history 82

Appendices

Appendix A: Laboratory procedures 85
Appendix B: Procedures at HVTU 86

FOR REVIEW ONLY

Detailed contents

Brief contents 3
Schema 8
Overview 9
Introduction 11
Ethical considerations 12
Foreword 13
HIV epidemic at HVTN clinical trials sites 15

Study products

1 Product background and rationale 18
1.1 Rationale for multiclade vaccines for HIV-1 18
1.2 Rationale for DNA and adenovirus-vectored vaccines 19
1.2.1 DNA Vaccines 19
1.2.2 Adenovirus-vectored vaccines 20
1.2.3 Expected distribution of Ad5 neutralizing antibody titers 22
1.2.4 Rationale for prime-boost vaccine strategies 23
2 Study product descriptions 25
2.1 Source and characteristics of the HIV gene inserts 25
2.2 DNA vaccine plasmids: VRC-HIVDNA016-00-VP 26
2.3 Biojector® 2000 Needle-free Injection System 26
2.4 Recombinant adenoviral vectors: VRC-HIVADV014-00-VP 26
3 Preclinical studies 28
3.1 Summary of preclinical safety studies: VRC DNA plasmids 28
3.1.1 Biodistribution of VRC-HIVDNA006-00-VP 28
3.1.2 Repeat-toxicity of VRC-HIVDNA006-00-VP 28
3.2 Summary of preclinical safety studies: VRC rAd and DNA prime followed by rAd boost 29
3.2.1 Repeat-toxicity of DNA prime/rAd5 boost HIV vaccine regimen 29
3.3 Summary of preclinical immunogenicity studies 30
4 Clinical studies 33
4.1 DNA plasmid vaccine trials 33
4.1.1 Protocol VRC 001 33
4.1.2 Protocol VRC 004 34
4.1.3 Protocol HVTN 044 35
4.1.4 Protocol HVTN 052 36
4.1.5 Protocol RV 156 36
4.1.6 Protocol VRC 007 36
4.2 Recombinant adenoviral vector vaccine trials 37
4.2.1 Protocol VRC 006 38
4.2.2 Protocol HVTN 054 39
4.3 Prime-boost trials 39
4.3.1 Protocol HVTN 057 39
4.3.2 Protocol VRC 009 40
4.3.3 Protocol VRC 008 40
4.3.4 Protocol VRC 010 40
5 Summary 41
5.1 Rationale for trial design 41

FOR REVIEW ONLY

5.2 Plans for future product development and testing....42

Study design

6 Study objectives....44
6.1 Primary objectives....44
6.2 Secondary objectives....44
7 Study type, study population, and eligibility criteria....45
8 Safety and immunogenicity evaluations....48
8.1 Considerations for trial start....48
8.2 Distinguishing intercurrent HIV infection from vaccine-induced positive serology....48
8.3 Immunogenicity evaluation....49
8.3.1 Humoral immunogenicity studies....49
8.3.1.1 Binding antibodies by ELISA (HVTN)....49
8.3.1.2 Neutralizing antibody assay (HVTN)....49
8.3.1.3 Binding and neutralizing antibody assays (VRC)....49
8.3.1.4 Neutralizing antibody against viral vector (Ad5)....49
8.3.2 Cellular immunogenicity studies....50
8.3.2.1 IFN-γ ELISpot (HVTN and VRC)....50
8.3.2.2 Intracellular cytokine staining (HVTN and VRC)....50
8.4 HLA typing....50
8.5 Ancillary studies....50
9 Statistical considerations....51
9.1 Overview....51
9.2 Objectives....51
9.3 Endpoints....51
9.3.1 Safety....51
9.3.2 Immunogenicity....51
9.3.3 Social impacts....52
9.4 Accrual and sample size....52
9.4.1 Sample size calculations for safety....53
9.4.2 Sample size calculations for immunogenicity....53
9.4.3 Sample size calculations across HVTN 204, RV172, and IAVI-V001....54
9.5 Statistical analysis....55
9.5.1 Analysis variables....55
9.5.2 Baseline comparability....55
9.5.3 Safety analysis....55
9.5.4 Immunogenicity analysis....56
9.5.5 Social impact analysis....57
9.5.6 Interim analyses....57
9.6 Randomization of treatment assignments....58

Study operations

10 Protocol conduct....60
11 Informed consent....61
11.1 Screening consent form....61
11.2 Protocol-specific consent form....61
11.3 Assessment of understanding....61
12 Procedures....63
12.1 Pre-enrollment procedures....63
12.2 Post-enrollment procedures....63

FOR REVIEW ONLY

12.3 Total blood volumes....64
12.4 Laboratory procedures....64
13 Study product preparation and administration....65
13.1 Schema and vaccine regimen....65
13.2 Study product formulation and preparation....65
13.2.1 VRC-HIVDNA016-00-VP (DNA)....65
13.2.2 DNA Placebo (phosphate buffered saline, PBS)....66
13.2.3 VRC-HIVADV014-00-VP (multiclade HIV-1 recombinant adenoviral vector vaccine, rAD)....66
13.2.4 VRC-DILUENT013-DIL-VP (final formulation buffer, FFB, placebo)....66
13.2.5 Labeling procedures to preserve blinding....67
13.3 Study product administration....67
13.3.1 VRC-HIVDNA016-00-VP (DNA) vaccine or placebo (PBS)....67
13.3.2 VRC-HIVADV014-00-VP (rAD vector) or placebo (labeled as VRC-DILUENT013-DIL-VP)....67
13.4 Study product acquisition....68
13.5 Pharmacy records....68
14 Safety monitoring and review....69
14.1 Assessing reactogenicity....69
14.2 Grading of adverse events....69
14.3 Adverse event reporting and safety pause/AE review rules....69
14.3.1 Adverse events to which safety pause/AE review rules apply....70
14.3.2 PSRT AE review and safety pause....70
14.3.3 Review and notification following safety pause....71
14.4 Expedited adverse event reporting to DAIDS....71
14.4.1 EAE reporting level....71
14.4.2 Study agents for expedited reporting to DAIDS....71
14.4.3 Grading severity of events....71
14.4.4 EAE reporting periods....71
14.5 Participant departure from schedule of vaccinations....72
14.5.1 Delaying vaccinations for a participant....72
14.5.2 Stopping vaccinations for a participant....72
14.5.3 Participant termination from the study....72
14.6 Study termination (for all participants)....73
14.7 HVTN review of cumulative safety data....73
14.7.1 Daily review....73
14.7.2 Weekly review....73
14.7.3 Quarterly review....74
14.7.4 Safety Monitoring Board review....74
References....75
Protocol history....82

Appendices

Appendix A: Laboratory procedures....85
Appendix B: Procedures at HVTU....86

Tables and figures

Table 1-1 Ad5 neutralizing antibody titers tested by Merck & Co., Inc. [76]....23
Table 1-2 Ad5 neutralizing antibody titers tested by the VRC....23

FOR REVIEW ONLY

Table 3-1 Summary of preclinical immunogenicity studies 32
Table 4-1 Multiclade HIV DNA vaccine experience in uninfected participants as of May 2005 33
Table 4-2 Multiclade HIV adenoviral vaccine, VRC-HIVADV014-00-VP, experience in uninfected participants as of May 2005..... 38
Table 7-1 Study inclusion criteria..... 46
Table 7-2 Study exclusion criteria 47
Table 9-1 Probability of response for different safety scenarios for vaccinees (n=240)..... 53
Table 9-2 Exact two-sided confidence intervals (CI) for the CD8+ T-cell response rate based on observing a particular rate of responses in vaccinees (n=216). CI's for larger loss-to-follow-up (LTFU) rates are also shown, n=204 with 15% LTFU, n=192 with 20% LTFU. 54
Table 9-3: Power to detect a difference in at least one of the 3 Fisher's Exact tests. 54
Table 12-1 Pre-enrollment procedures..... 63
Table 12-2 Post-enrollment procedures 64
Table 14-1 Schedule of reactogenicity assessments 69
Table 14-2 Adverse event notification and safety pause/AE review rules..... 70
Protocol history and modifications 82

FOR REVIEW ONLY

Schema

Study products

DNA vaccine: HIV-1 DNA vaccine VRC-HIVDNA-016-00-VP (clade B Gag, Pol, and Nef; clades A, B, and C Env)

DNA placebo: Phosphate buffered saline (PBS)

rAd5 vaccine: HIV-1 recombinant adenoviral vector vaccine VRC-HIVADV014-00-VP (clade B Gag-Pol fusion; clades A, B, C Env)

rAd5 placebo: Adenoviral vector final formulation buffer (FFB) VRC-DILUENT013-DIL-VP

Routes of injection

DNA and PBS: IM via needle-free Biojector

rAd5 and FFB: IM via needle and syringe

Study arm	Americas	southern Africa	Vaccination schedule in months (days)			
			0 (0)	1 (28)	2 (56)	6 (168)
Group 1	120	120	4 mg DNA	4 mg DNA	4 mg DNA	10^{10} PU rAd5
Group 2	120	120	PBS	PBS	PBS	FFB
Total	240	240				

PU = viral particle units

FOR REVIEW ONLY

Overview

Title

A Phase II clinical trial to evaluate the safety and immunogenicity of a multiclade HIV-1 DNA plasmid vaccine, VRC-HIVDNA016-00-VP, followed by a multiclade recombinant adenoviral vector HIV-1 vaccine boost, VRC-HIVADV014-00-VP, in HIV-1 uninfected adult participants

Participants

Healthy HIV-1-uninfected adult participants (18 to 50 years old)

Number of participants

Total 480: 240 vaccine, 240 placebo

Primary objectives

Safety

To evaluate the safety and tolerability of 3 doses of 4.0 mg of an HIV-1 DNA vaccine followed by 1 dose of an adenoviral vector vaccine at 1 x 10^{10} viral particle units.

Immunogenicity

Evaluate the immunogenicity of 3 VRC HIV-1 DNA-6-plasmid vaccine doses at 4.0 mg/dose followed by VRC HIV-1 rAd5 vaccine boost at 10^{10} PU in HIV-1 uninfected adults.

Study products

DNA plasmid vaccine: VRC-HIVDNA-016-00-VP

VRC-HIVDNA-016-00-VP is composed of six DNA plasmids in equal concentrations that encode Gag, Pol, and Nef from clade B (strains HXB2, NL4-3, NY5/BRU) and the HIV-1 Env glycoproteins from clade A (strain 92rw020), clade B (strains HXB2/BaL), and clade C (strain 97ZA012).

Recombinant adenoviral vector vaccine: VRC-HIVADV014-00-VP

VRC-HIVADV014-00-VP is a recombinant product composed of 4 adenoviral vectors (Ad) (in a 3:1:1:1 ratio) that encode the HIV-1 Gag-Pol polyprotein from clade B (strains HXB2-NL4-3) and HIV-1 Env glycoproteins from clade A (strain 92rw020), clade B (strains HXB2/Bal), and clade C (strain 97ZA012).

DNA placebo

The placebo for the DNA vaccine, VRC-HIVDNA-016-00-VP, is sterile phosphate buffered saline (PBS).

Adenoviral vector placebo

The placebo for the adenoviral vector vaccine, VRC-HIVADV014-00-VP, is adenoviral final formulation buffer (FFB), VRC-DILUENT013-DIL-VP.

Study design

Multicenter, randomized, placebo-controlled, double-blind trial

FOR REVIEW ONLY

Study duration

12 months per participant

Safety monitoring

HVTN 204 Protocol Safety Review Team

HVTN Safety Monitoring Board

Vaccine provider

Dale and Betty Bumpers Vaccine Research Center (VRC), National Institute of Allergy and Infectious Diseases (NIAID), National Institutes of Health (NIH) (Bethesda, Maryland, USA)

IND sponsor

Division of AIDS (DAIDS), NIAID, NIH, Department of Health and Human Services (DHHS) (Bethesda, Maryland, USA)

Study sites

US and non-US HIV Vaccine Trial Units (HVTUs)

HVTN Core Operations

HVTN Vaccine Leadership Group/Core Operations Center, Fred Hutchinson Cancer Research Center (FHCRC) (Seattle, Washington, USA)

Statistical and data management center

Statistical Center for HIV/AIDS Research and Prevention (SCHARP), FHCRC

HVTN Endpoint laboratories

Duke University Medical Center (Durham, North Carolina, USA)

FHCRC/University of Washington (Seattle, Washington, USA)

Viral and Rickettsial Disease Laboratory (Richmond, California, USA)

South Africa Immunology Laboratory and National Institute for Communicable Disease (Johannesburg, South Africa)

FOR REVIEW ONLY

Introduction

The ongoing worldwide epidemic of the human immunodeficiency virus type 1 (HIV-1) remains one of the major global health challenges. HIV-1 causes the acquired immunodeficiency syndrome (AIDS), which is responsible for tremendous human suffering and economic loss throughout the world. Currently, over 42 million people are living with HIV-1 infection [1]. Without treatment, it is likely that nearly all of these will die of AIDS in the next 2 decades.

Since 1996, potent new antiretroviral therapies, including combination regimens with protease inhibitors, have created the possibility that HIV-1 infection might become a chronic, manageable disease among individuals with access to these medications. In the US, AIDS deaths are down to 16,000 per year as a result of the new antiretrovirals [2]. However, for the developing world, where over 95% of the 5 million annual incident HIV-1 infections occur [1], it is unlikely that these drugs will be widely accessible, due to many logistical challenges associated with their use.

Globally, 14,000 new infections occur each day. More than 3 million AIDS deaths occur per year [1], and nearly 20 million have died since the HIV epidemic began [3]. AIDS has become the leading infectious disease killer, the fourth leading cause of death overall. In severely affected countries, life expectancy has fallen by more than 10 years [1]. AIDS is the leading killer in Africa, with over 28 million Africans living with HIV/AIDS. Sub-Saharan Africa has been affected most; in 7 Sub-Saharan African countries, over 20% of adults (aged 15 to 49) are living with HIV/AIDS [3]. For example, in Botswana, 38.8% of adults aged 15 to 49 are infected with HIV, while in South Africa 24.8% of women in antenatal clinics are infected [3].

The need for better education, better treatment access, better prevention programs, and better prevention technologies is therefore clear. Specifically, the need for a safe, effective, and affordable HIV-1 vaccine is paramount [4,5]. The ideal HIV-1 vaccine for global use should meet several of the following criteria:

- proven safety in healthy HIV-uninfected persons
- induction of long-lasting HIV-specific cell-mediated and humoral immunity capable of conferring protection against HIV
- tolerability
- potential for production in sufficient quantity to meet global needs
- affordability
- stability during distribution and storage

FOR REVIEW ONLY

Ethical considerations

Multiple candidate HIV vaccines will need to be studied simultaneously in different populations around the world before a successful HIV preventive vaccine is found. It is critical that universally accepted ethical guidelines are followed at all sites involved in the conduct of these clinical trials. The HVTN has addressed ethical concerns in the following ways:

- HVTN trials are designed and conducted to enhance the knowledge base necessary to find a preventive vaccine, with methodology that is scientifically rigorous and valid, and in accordance with Good Clinical Practice (GCP) guidelines.
- HVTN scientists and protocol team members incorporate the philosophies underlying major codes, declarations, and other guidance documents relevant to human subject research into the design and conduct of HIV vaccine clinical trials.
- HVTN scientists and protocol team members are committed to substantive community input into the planning, conduct, and follow up of the research which will help ensure that locally appropriate cultural and linguistic needs of study populations are met.
- The HVTN advocates that all HVTN sites should develop a plan for the care and treatment of participants who develop HIV infection during a trial. This plan should be formulated by representatives of host countries, communities from which potential trial participants will be drawn, sponsors, and the HVTN.
- Prior to implementation, HVTN trials are rigorously reviewed by both local and national regulatory bodies, in addition to scientists who have no involvement with the trial under consideration.
- The HVTN recognizes the importance of institutional review and values the role of in-country Institutional Review Boards (IRBs) and Institutional Ethics Committees (IECs) as custodians responsible for ensuring the ethical conduct of research in the local setting.
- The HVTN provides training so that all participating sites similarly ensure fair subject selection, protect the privacy of research subjects, and obtain meaningful informed consent.

FOR REVIEW ONLY

Foreword

This study, HVTN 204, and two others, RV 172 and IAVI-001, are conducted with the Vaccine Research Center (VRC), U.S. National Institute of Allergy and Infectious Diseases (NIAID) of the U.S. National Institutes of Health (NIH) to qualify their six-plasmid multiclade HIV-1 DNA vaccine boosted with a multiclade HIV-1 recombinant adenovirus-5 vector (rAd5) vaccine for efficacy trials.

These three studies are being conducted by three independent trial networks and each network consists of non-overlapping national and international partnerships to execute vaccine clinical trials. The main objective of these three trials taken together is to attain safety and immunogenicity data sufficient to proceed with efficacy testing within each of the networks. Phase II safety data that support the safety of this prime-boost vaccine approach as well as immunogenicity data that show distinct improvement, relative to products currently in Phase III clinical trials (ALVAC and GP120), in terms of breadth or quantitative immune responses, are needed to move into an efficacy trial.

All three networks are part of the Partnership for AIDS Vaccine Evaluation (PAVE). PAVE is a consortium of organizations that are actively involved in HIV vaccine clinical research and development and includes the Division of AIDS (DAIDS) NIAID-NIH, Vaccine Research Center (VRC), NIAID-sponsored HVTN and U.S. Military HIV Research Program (USMHRP), the Centers for Disease Control and Prevention (CDC), and the International AIDS Vaccine Initiative (IAVI). This consortium is charged with bringing their cumulative global expertise to bear on the conduct of large-scale efficacy trials.

The HVTN, the USMHRP, and the IAVI, working closely with international host site investigators from each one of the countries to implement the studies, wrote HVTN 204, RV 172 and IAVI-V001, respectively. The combined study data will constitute the primary end of Phase II data set for safety and immunogenicity of this prime-boost Investigational New Drug (IND). It is crucial that all three trials, although independent from one another in implementation, are developed with sufficient harmonization to guarantee compatibility of the respective data sets for aggregated analyses.

The three studies will provide data from the U.S. and countries in eastern Africa, the Caribbean, South America, and southern Africa. The regions are defined as follows:

- United States of America, South America, and the Caribbean in HVTN 204
- Southern Africa in HVTN 204
- Eastern Africa in RV 172 and IAVI-V001

All three trials will be conducted in accordance with current Good Clinical Practice (cGCP), International Conference on Harmonization (ICH) guidelines and the revised U.S. Code of Federal Regulations—21 and 45 CFRs. Copies of all the above documents and any other information and/or guidelines that are applicable for the safe and ethical conduct of the study will be available at each clinical site. The U.S. FDA will be the regulatory agency granting the IND application for the three studies. However, all country specific regulations for approval, implementation, and reporting will be followed as applicable.

The three organizations worked with the vaccine developer, VRC, as well as with the studies' sponsor, the DAIDS NIAID-NIH, to harmonize all three trials during the development phase of the protocols. This harmonization was done to guarantee that the three trials will collect information from each study population using standardized terms and definitions, so that the data can be merged and used to move this vaccination approach into efficacy testing. Although the populations will be different given the diverse geographic locations and associated variations in socio-economic and medical infrastructure, virtually identical inclusion and exclusion criteria will be applied among the three trials.

Furthermore, the harmonization of the three trials will continue through the implementation period of the studies with continued communication and meetings, as needed, to discuss any elements of one trial that may affect and/or trigger modification of all three trials. Although safety management of each trial will be

FOR REVIEW ONLY

independently followed for each protocol, there will be a continued and open exchange of safety information channeled through the common DAIDS Medical Officer for all three trials and the vaccine developer. In addition, members of each network will be available to participate in other network's protocol safety reviews teams (PSRTs).

The Phase I part of IAVI-V001 and RV 172 will be focused on evaluating the safety and immunogenicity of two dose levels of rAd5 in participants with a broad range of preexisting Ad5 antibody titers, with and without prior DNA priming. These studies will complement VRC 006 and VRC 008 to determine whether additional effort should be given to the evaluation of the 10^{11} PFU dose of rAd5.

Finally, the three studies together, RV 172, HVTN 204, and IAVI-V001, are powered to detect small differences in safety and tolerability (between vaccine and placebo); and RV 172 and HVTN 204, the Phase II studies, are powered to detect small differences in immunogenicity between the vaccine and placebo arms.

In view of the collective value of these three studies and the effort to harmonize, a single protocol was considered. However, the infrastructure development and training needed to conduct this effort as a single, integrated study would have required considerable cost and time which can only be justified for implementation of an efficacy trial. The time for such an effort would have delayed acquisition of the critical data. It was judged by the studies' sponsor, the vaccine developer, and the participating networks to be both more efficient and judicious use of available funds to quickly determine if additional investment for a PAVE directed efficacy trial is warranted. The three study approach will allow all three studies to explore their own secondary objectives, while assuring harmony of primary objectives.

FOR REVIEW ONLY

HIV epidemic at HVTN clinical trials sites

The VRC candidate vaccines being tested in HVTN 204 are multi-clade A/B/C immunogens, and as such are intended for use in the widespread geographic regions where these subtypes circulate. The HVTN is a global clinical research network, including multiple clinical trials sites in areas where subtype B and C infections are transmitted. Specifically, the HVTN has 12 clinical sites in the US, 5 in the Caribbean region, and 4 in South America where subtype B infection is predominant, and there are 4 clinical trials sites in southern Africa where subtype C circulates. A subset of these sites will be chosen to participate in HVTN 204. The widespread geographic composition of the network not only allows for access to a large number of study participants in HIV subtype-appropriate regions, but also allows for information to be gathered on toxicity profiles and immune responses in regions of the world where the population background Ad5 seroprevalence varies appreciably.

Sub-Saharan African countries continue to bear the brunt of the global HIV/AIDS pandemic, having only just over 10% of the world's population, but over 60% of the world's HIV-infected people [1]. Southern Africa continues to be most severely affected subregion in the world with seroprevalence in urban antenatal clinics of over 25%. The predominant mode of transmission is through heterosexual contact and females constitute 57% of HIV infected people in Sub-Saharan Africa. HVTN 204 is being planned to be carried out at sites in two of the hardest hit countries in the world, South Africa and Botswana, where the HIV prevalence has been found to be as high as 25-33% among young adults, and the annual HIV incidence is estimated to be in the range of 4-6%.

The Americas, in general, are much less severely affected than Africa, although there are some areas and population groups that clearly bear a disproportionate burden of the disease. In the United States, the seroprevalence rate is estimated to be approximately 0.5% among the general population, and the epidemiology has evolved over the years from being more heavily concentrated in the male homosexual and bisexual population to now being increasingly found among racial/ethnic minorities (especially women) who have acquired it through heterosexual contact and injection drug use. Indeed, HIV/AIDS ranks among the top three causes of death in African American men between the ages of 25-54 and women between the ages of 35-44 years. In Latin America, HIV was likely introduced into the population roughly the same time as in the US and the overall seroprevalence rate is estimated to be 0.6%. Brazil, the continent's largest country, has seen the epidemic spread from the major urban areas into all regions of the country and like in the US has evolved to increasingly affect women through heterosexual contact. While the overall population seroprevalence rate in Brazil is approximately 0.7%, it may be as high as 23% in high-risk groups in some regions. The Caribbean region is the second most highly affected region in the world, and AIDS is the leading cause of death for adults between the ages of 15-44. The overall prevalence in adults in the region is estimated to be at 2.3%. HIV transmission in the Caribbean region has long been concluded to be primarily by heterosexual contact, and it is estimated that new infections are now twice as likely to occur in women. Haiti has the largest number of people living with HIV in the region and the latest round of HIV surveillance data from antenatal clinics shows a rate between 1.8% and 7%, depending on the location, with poor and uneducated women having higher rates than their more advantaged counterparts. The second largest number of cases of HIV in the Caribbean region is in Jamaica, with seroprevalence rates between 1.4-2.7% in antenatal clinics and between 5-8% in STD clinics. In addition to sites in southern Africa, HVTN 204 is being planned to be carried out at sites in the US, South America, and the Caribbean.

FOR REVIEW ONLY

This page is intentionally blank

FOR REVIEW ONLY

STUDY PRODUCTS

FOR REVIEW ONLY

1 Product background and rationale

1.1 Rationale for multiclade vaccines for HIV-1

HIV has an enormous potential to generate genetically diverse variants because of the high error rate of reverse transcription, large viral burden, high replication rate, and pressure from immune defenses and anti-retroviral treatment. A recent analysis of genomic sequences from different regions in the world has identified at least 9 major subtypes (A, B, C, D, F, G, H, J and K) and dozens of recombinant forms [6]. Genetic diversity occurs within the individual patient as well with as much as 10% difference noted in the variable viral envelope sequences within a single infected patient [7-9]. A major challenge to designing HIV vaccines is to identify and target viral structures that are the critical determinants for protective humoral and cellular responses across the widest possible range of diversity. Genetic and antigenic variation is a particular problem for generating neutralizing antibody responses against HIV. The envelope glycoprotein is the most variable among the HIV genes, and the antibody response elicited tends to be type-specific. In contrast, CD8+ T cells can recognize epitopes from internal structural and regulatory proteins in addition to the envelope glycoprotein, and these frequently occur within more highly conserved regions [10]. For this reason, CD8+ T-cell responses may be more broadly cross-reactive than many antibody responses.

While the immunological relevance of HIV genetic diversity is not fully understood, studies of HIV vaccine volunteers and infected patients suggest that a successful global HIV vaccine program will need to protect against the diverse strains and subtypes predominating in the target populations, as has been the case for influenza. In a report from Merck & Co., Inc., the frequency of T cell immune responses recognizing clade A and clade C determinants appeared lower than to clade B in volunteers with a positive response to recombinant adenoviral-Gag (clade B) vaccine; among the 13 vaccinees with PBMC reactive to clade B peptides, 77% had PBMC recognizing pools of either clade A (10/13) or C (10/13) Gag peptides [11]. Lacking a thorough understanding of protective targets over the broad array of circulating HIV strains, a globally successful vaccine will most likely need to include many HIV antigens to represent the full range of variants that will need to be controlled. An analysis of HIV genetic variation and CTL epitopes based on data in the Los Alamos database has concluded that there may be an advantage in using a consensus sequence or an isolate from within each clade for capturing the largest number of relevant T cell epitopes in the vaccine immunogen [12,13]. The use of multivalent vaccines, containing a defined mixture of immunogens from a number of prevalent subtypes is a feasible approach to achieve broadly protective HIV vaccines. The World Health Organization / UNAIDS HIV Vaccine Advisory Committee has recommended that candidate HIV vaccines be designed based upon the strains prevalent in the country in which trials are to be conducted [14]. This approach is the foundation for the design of VRC-HIVDNA016-00-VP and VRC-HIVADV014-00-VP, which incorporate HIV *gag*, and *pol* (and *nef* in VRC-HIVDNA016-00-VP), genes from clade B as well as more diverse *env* genes from clades A, B, and C, which together represent the viral subtypes responsible for about 90% of new HIV infections in the world [15].

FOR REVIEW ONLY

1.2 Rationale for DNA and adenovirus-vectored vaccines

1.2.1 DNA Vaccines

DNA vaccines have been tested in animal models and clinical trials for a variety of different pathogens, including influenza [16], malaria [17,18], and hepatitis B [19], in addition to HIV-1. Typically, these studies have shown that the vaccines are safe and well-tolerated, and there has been no evidence of induction of antinuclear or anti-double stranded DNA antibodies. However, overall immunogenicity has been generally disappointing and various strategies to improve the immunogenicity profile have been a major area of research over the last several years. Optimization strategies include studies of dose escalation and vaccination route comparison, and the published results so far suggest that DNA vaccines can be delivered safely at single doses ranging from 250 µg to 5 mg of DNA, with one trial reaching a cumulative dose of 20 mg after multiple vaccinations [11]. Also, it is postulated that the lack of potent immunogenicity in humans and nonhuman primates is a technical issue that can be overcome by experimentation with improved expression and/or enhanced delivery systems. Gene expression can be augmented by stronger gene enhancers and by improved translational efficiency of foreign DNA in mammalian cells through modifications to human codon sequences [20,21]. In addition, new DNA formulations given with cytokine adjuvants may improve immunogenicity [22-24].

Many novel HIV-specific DNA constructs have been tested in animal models, and the key research now focuses on immunogenicity and efficacy in nonhuman primates and humans [25]. Protection from SHIV challenge has been demonstrated after DNA vaccination alone, without a heterologous boost, when given in combination with a plasmid IL-2/Ig adjuvant [26] while several studies show protection in prime-boost experiments (see Section 1.2.4).

The first series of human clinical trials of an anti-HIV DNA vaccine involved 2 different vaccines, one expressing Env and Rev proteins, and the other expressing Gag and Pol proteins, which were developed by Apollon and Wyeth Lederle Vaccines, Inc. Two studies were carried out with the Env/Rev vaccine in HIV-infected subjects with CD4 counts >500. The Env/Rev vaccine was also tested in a small phase I study in HIV-negative subjects at the NIH Clinical Center. Shortly thereafter, the Gag/Pol DNA vaccine was tested in the NIAID-supported AIDS Vaccine Evaluation Group (AVEG 031), also in HIV-negative participants. In all these studies, the plasmid was administered with bupivacaine to enhance DNA uptake, gene expression, and immune responses. Doses ranged in these studies from 30 mcg to 3 mg, and vaccines were generally administered intramuscularly up to 4 times over a period of 6 months. These studies provided extensive safety data, including long-term follow-up for at least 2.5 years, and revealed no significant clinical or laboratory findings in any of the subjects [27,28]. Sporadic low level T lymphocyte immune responses were seen in these studies, but the conclusions were generally limited by the small number of subjects per group [29].

Subsequently, a number of clinical trials with DNA vaccines have been conducted by the HVTN, Merck Vaccines, the International AIDS Vaccine Initiative (IAVI) and the Australian-New South Wales group. In most of the studies, the DNA vaccine was or will be administered as prime in prime-boost regimens with a variety of booster vaccines [30-33]. To date, the HVTN has tested DNA vaccines developed by Emory University, Epimmune Inc., and Chiron Corp. in small phase I trials. The largest experience with anti-HIV DNA vaccines comes from the Merck program, which has administered their DNA gag vaccine to over 300 HIV-uninfected volunteers [James Kublin, personal communication]. The cumulative experience with all these vaccines reflects the findings in the non-HIV DNA vaccine studies,

FOR REVIEW ONLY

i.e., they are safe and well-tolerated, but induce only modest B- and T-cell stimulation when given alone [30-33].

The Vaccine Research Center, NIAID, NIH in collaboration with the Division of AIDS (DAIDS), NIAID, NIH has previously sponsored two intramural clinical trials with two different HIV-1 DNA vaccines. These are a single plasmid clade B Gag-Pol vaccine (VRC 4302) and a multiclade Gag-Pol-Nef and Env, 4-plasmid vaccine (VRC-HIVDNA009-00-VP). A total of 65 subjects have received these DNA vaccines in doses ranging from 0.5-8 mg, and the safety data to date indicates that these DNA vaccines are well tolerated and safe in healthy volunteers. The cellular immunogenicity of VRC-HIVDNA009-00-VP through week 12, as measured by intracellular cytokine staining (ICS) and enzyme-linked immunospot (ELIspot), show good CD4+ responses to the Env immunogens, but responses to the Gag, Pol and Nef are weak to absent. CD8+ responses are less pronounced than CD4+ (more information on this study can be found in Section 4). In addition, VRC-HIVDNA009-00-VP is also now undergoing further evaluation in two other Phase I clinical trials conducted by the HVTN, HVTN 052, which is fully enrolled and evaluating two- versus three-injections of the multiclade DNA vaccine, VRC-HIVDNA009-00-VP, in 120 participants, and HVTN 044, is evaluating VRC-HIVDNA009-00-VP in combination with escalating doses of a plasmid IL-2/Ig adjuvant in 70 participants. Safety data from the studies again indicates that the DNA vaccines are well-tolerated, while immunogenicity results from the HVTN studies are currently pending. See Section 4 for a more complete presentation of the clinical data.

The rationale for the development of the current 6-plasmid vaccine, VRC-HIVDNA016-00-VP, derives from the poor immunogenicity of the Gag, Pol and Nef components of the 4-plasmid vaccine in the intramural study. The new generation vaccine separates the gag, pol and nef genes into separate plasmids, rather than having one plasmid that produces a fusion protein immunogen. In addition to splitting the genes into separate plasmids there are two other changes in the plasmid construction. These are: 1) a change in the promoter (to CMV/R) incorporated into these plasmids and 2) a 68 amino acid addition to the gag gene in the VRC 4401 (Gag protein only) plasmid as compared to the VRC 4306 (Gag-Pol-Nef fusion protein) plasmid that was in VRC-HIVDNA009-00-VP. The CMV/R promoter consists of the translational enhancer region of the CMV immediate early region 1 enhancer substituted with the 5'-untranslated HTLV-1 R-U5 region of the human T-cell leukemia virus type 1 (HTLV-1) long terminal repeat (LTR) to optimize gene expression further.

Preclinical data in monkeys with VRC-HIVDNA016-00-VP indicate that the separate gag, pol and nef plasmids generate more consistent and stronger immune responses to the Gag, Pol and Nef immunogens than were produced by the fusion protein immunogen. The clades A, B and C env plasmids in VRC-HIVDNA016-00-VP are identical to the three env plasmids in VRC-HIVDNA009-00-VP except that the promoter CMV/R has been used rather than the previous CMV promoter. Therefore the vaccine is expected to elicit immune responses against several proteins from a variety of HIV-1 strains and incorporates the safety features of previous HIV-1 vaccines, while potentially improving the immunogenicity.

1.2.2 Adenovirus-vectored vaccines

Replication-competent adenovirus vaccines in oral form have been administered to millions of military personnel, and over 42,000 have participated in well-controlled clinical trials to evaluate the safety and efficacy of these agents. These studies are well summarized in the literature [34-38] and have shown these vaccines to be safe and highly effective in preventing acute respiratory disease in recruits. The potential risk for live adenovirus to cause serious infection in immunocompromised hosts and potential concerns for oncogenic potential of

FOR REVIEW ONLY

some serotypes have lead to the development of replication-defective adenoviral vectors as vaccines and gene therapy vectors.

Adenoviral vectors incorporating a variety of expressed proteins have been used for gene therapy studies in many different human diseases, including cancer, cystic fibrosis, and cardiovascular disease, and have been delivered via several routes of administration including aerosol, intradermal, intramyocardial, intravenous, intrapleural and intratumoral [39-52]. Hundreds of human subjects have taken part in studies to evaluate adenoviral vectors as gene therapy agents, with many of these evaluating vectors based upon adenovirus type 5. Data from these and other studies found that side effects of adenoviral vectors were minor, local, or absent in most cases where the agents were administered intradermally or intramuscularly, with no significant vector-induced toxicities. One study assessed ten clinical trials for the safety parameters [53] and risk factors [50] of low (<10^9 particle units, PU)- and intermediate (10^9-10^{10} PU) – dose adenoviral vectors, delivered by various routes (nasal, bronchial, percutaneous injection into solid tumor, intradermal, epicardial injection of myocardium, and injection of skeletal muscle) to 90 individuals and 12 controls for treatment of a variety of conditions (cystic fibrosis, colon cancer metastases, severe coronary artery disease, and peripheral vascular disease). Local administration of these doses of adenoviral vectors appeared to be well tolerated. The major adverse events seemed to be primarily associated with characteristics of the study population (age, co-morbid conditions) and/or trial procedures (surgery) rather than dose, route of administration, expressed transgene, or number of administrations.

In the context of a large body of clinical research experience with adenovirus-based vectors, the death of a teenager in a gene therapy trial at the University of Pennsylvania prompted extensive reviews of safety data from both human and animal studies of these agents, many at the direction of the NIH Recombinant DNA Advisory Committee (RAC) and the Food and Drug Administration (FDA). The 18 year-old patient, suffering from ornithine transcarbamylase deficiency (OTCD), died after receiving a dose of 3.8 x 10^{13} PU of an E1/E4–deleted serotype 5 adenoviral vector directly into the hepatic artery [54]. An NIH report summarizing a review of clinical data from the case concluded that the participant's death was most likely due to a systemic adenoviral vector-induced shock syndrome, caused by a cytokine cascade that led to disseminated intravascular coagulation, acute respiratory distress syndrome, and multiorgan failure. Post-mortem bone marrow biopsy revealed red cell aplasia. The data suggested that the high dose of adenoviral vector delivered directly to the liver quickly saturated available receptors for the vector in that organ, leading to systemic dissemination which induced the fatal immune response [55].

In the University of Pennsylvania study, 19 OTCD patients received doses ranging from 1.86 x 10^{11} to 3.8 x 10^{13} E1/E4–deleted adenoviral vector particles infused directly into the hepatic circulation. The trial was halted after the death described above. Essentially all study subjects experienced one or more of the following: fevers, myalgias, nausea, and occasional emesis. Nearly all subjects showed a mild and transient thrombocytopenia without consistent abnormalities in coagulation, and higher dose levels were associated with subsequent abnormal liver function studies [54]. Similar results have been observed in animal studies [56-61] with the implication that systemic administration of adenoviral vectors might cause liver abnormalities and fever and might be associated with these symptoms more often than in patients treated via other routes of administration. Close clinical monitoring to detect such symptoms will be carried out in the present study and the adenoviral vectors will be administered by intramuscular injection, with a maximum study dose of 10^{10} PU.

Significant preclinical and clinical evidence also exists indicating that immune responses against HIV and other pathogens can be induced by direct gene transfer of immunogen-

FOR REVIEW ONLY

expressing genes via recombinant adenoviral vectors. Studies in non-human primates have shown that replication-incompetent serotype 5 (Ad5) adenoviral vectors can generate cellular immune responses against several viruses including HIV-1, SIV and Ebola [62-66]. Strong cellular immune responses were demonstrated using an IFN-γ ELISpot assay in baboons immunized with 10^{11} particles of replication-incompetent Ad5, with HIV-1 Gag-specific T-cells as high as 0.2% of circulating lymphocytes [63]. Rhesus macaques immunized with SIV Gag-based Ad5 vectors showed potent cytotoxic T lymphocyte (CTL) responses that correlated with protection (reduced CD4 loss, contained acute and chronic viremia, and reduced morbidity and mortality when challenged with a pathogenic strain of SIV) [62].

Preexisting vector immunity appears to attenuate immunologic responses to Ad5 vaccines in animal studies, but evidence exists that this attenuation may be overcome by increasing vaccine doses or using a prime-boost approach or both. In a mouse model, preexisting immunity to Ad5 resulted in markedly reduced cytotoxic T-cell responses to Ebola virus glycoprotein following immunization with an adenoviral vector expressing the Ebola antigen, compared with mice without prior vector immunity. Priming the mice with DNA encoding the Ebola antigen largely reversed this attenuation of cellular immune response to the glycoprotein after boosting with the adenoviral vector. Humoral immune responses to the Ebola antigen were also significantly reduced in mice with prior immunity to the adenoviral vector, but were not significantly increased by DNA priming [67]. Macaque studies done by Merck with their SIV Gag-Ad5 vaccine also indicate that immune responses are attenuated by preexisting immunity to Ad5, and that increasing the dose of the vaccine may overcome preexisting immunity [11,68].

Reports from Phase I dose escalation clinical trials of recombinant adenovirus type 5 (Ad5) vector vaccines developed by Merck & Co., Inc. and encoding clade B HIV-1 Gag, which examined doses ranging from 10^8 to 10^{11} PU per injection, described the vaccines as well tolerated [11,68]. These studies found moderate and sporadic injection site reactions, as well as sporadic fever with malaise, chills, and body aches, apparently more common at higher doses of the vaccine [11,68]. All of these adverse events were self-limited, and typically resolved within 48 hours. Preexisting immunity to Ad5 appeared to be associated with differences in side effects and immunogenicity. Local and systemic reactions were more common in participants with low Ad5 neutralizing antibody titers at baseline, and were attenuated following a booster dose of the adenoviral vector [68]. These studies demonstrated ELISpot responses 4 weeks following the second injection in 43%- 91% of participants, depending upon dose level administered and preexisting Ad5 neutralizing antibody titer. The frequency of responses to the Ad5 HIV-1 Gag vaccine appeared to be influenced by preexisting humoral immunity to Ad5 at all dose levels. The beneficial immunogenic effect of increasing the vaccine dose appeared most marked in the high (>1:200) titer preexisting Ad5 neutralizing antibody group, but the number of subjects was small [68].

1.2.3 Expected distribution of Ad5 neutralizing antibody titers

Extensive population data on the global distribution of neutralizing antibodies (NAb) to Ad5 are limited, but appears to be widespread in the regions where it circulates, and is often acquired at an early age [69]. Most of the data comes from studies done in the United States or other developed countries, and suggest that at baseline roughly one third of adults in developed countries will be seronegative (titer <1:10 - 1:20) for Ad5-neutralizing antibodies, and roughly one third will have titers above the 1:200 range [11,51,68,70,71]. Considerable variation exists among studies, however, with some reporting detectable neutralizing antibodies to Ad5 in as few as one third of US subjects [72,73].

FOR REVIEW ONLY

An analysis of reports to WHO between 1967 and 1976 found no difference between Northern and Southern Hemispheres in the relative incidence of Ad5 among reported adenovirus infections [74]. However, recent studies in sub-Saharan Africa have found the seroprevalence of neutralizing antibodies to Ad5 to be approximately 80-90% [71,75]. Merck, WRAIR, the HVTN and the VRC have recently collected, or are in the process of collecting seroprevalence data on neutralizing titers to Ad5 in many countries that have a high incidence of HIV infection (Table 1-1 and Table 1-2). Preliminary studies suggest that the titer of 1:200 in the Merck assay is roughly equivalent to the titer of 1:500 in the VRC assay. The significant prevalence of Ad5 immunity in these countries necessitates clinical investigation of the effect of prior immunity on the immunogenicity of type 5 adenoviral vector vaccines, and the development of strategies to overcome this effect, before large-scale clinical trials of such HIV vaccines are undertaken.

Table 1-1 Ad5 neutralizing antibody titers tested by Merck & Co., Inc. [76]

	<18 (%)	18-200 (%)	>200 (%)
Brazil (n=183)	8.2	29.5	62.3
Botswana (n=63)	8.8	26.5	64.7
Cameroon (n=225)	8.9	28.9	62.2
Malawi (n=49)	12.2	32.7	55.1
South Africa (n=182)	10.4	21.4	68.2
Thailand (n=1006)	6.0	13.7	80.3
US (n=779)	38.8	25.4	35.8

Table 1-2 Ad5 neutralizing antibody titers tested by the VRC

	<12 (%)	12-500 (%)	>500 (%)
India (n=96)	19.8	29.2	51.0
Tanzania (n=53)	5.7	43.4	50.9
Uganda (n=110)	19.1	30.9	50.0
US (n=85)	50.6	10.6	38.8

1.2.4 Rationale for prime-boost vaccine strategies

Prime-boost regimens have shown promise in non-human primate models of HIV infection. Such regimens have the potential for raising high levels of immune responses. DNA vaccine priming followed by a recombinant viral vector boost with a modified vaccinia Ankara (rMVA) [77] or replication-deficient Ad5 [65] have been shown to attenuate a pathogenic SHIV infection in rhesus macaques, most likely by the generation of a $CD8^+$ CTL response. Merck Research Laboratories has published preclinical studies of priming for HIV-1 specific immunity using an adjuvant-formulated DNA vaccine followed with Ad5 vaccine boost. This generates levels of T-cell immune response that are comparable to those in naive animals receiving multiple high doses of Ad5 HIV-1 vaccines [78].

FOR REVIEW ONLY

A prime-boost regimen has also shown promise in a preclinical model for prevention of Ebola virus infection in a study sponsored by the VRC, NIH and the Special Pathogens Branch, CDC. Cynomolgus macaques immunized with a combination DNA plasmid (a mixture of four DNA plasmids encoding glycoproteins from three Ebola strains and nucleoprotein from one strain) and boosted with a replication-deficient adenoviral vector encoding the glycoprotein resisted lethal viral challenge [66].

These preclinical studies suggest that DNA plasmid vaccine priming, followed by replication-defective adenoviral vector vaccine boost, as part of a carefully crafted vaccine strategy, can elicit potent and protective T-cell immune responses in animal models.

FOR REVIEW ONLY

2 Study product descriptions

2.1 Source and characteristics of the HIV gene inserts

The VRC DNA-HIV vaccine (VRC-HIVDNA016-00-VP) and VRC Ad5-HIV vaccine (VRC-HIVADV014-00-VP) contain largely matched HIV gene inserts, but they are not identical. The Gag and Pol proteins in both vaccines exhibit highly conserved domains. In the Ad5 vaccine they are present as a fusion protein, whereas they are expressed by separate plasmids in the DNA-HIV vaccine. In addition, the DNA-HIV vaccine includes a plasmid that encodes for Nef, whereas Nef is not included in the Ad5-HIV vaccine mixture. The clade A, B and C Env's are synthetic versions of modified, truncated envelope glycoproteins (gp145 in the DNA-HIV vaccine and a slightly shorter gp140 in the Ad5-HIV vaccine).

Gag

The synthetic *gag* gene in both vaccines is from HIV-1 clade B strain HXB2. In order to construct the fusion Gag-Pol expressed by the Ad5-HIV vaccine, a sequence encoding a 68 base pair amino acid sequence was deleted from the *gag* gene. No additional amino acid modifications were made to the *gag* gene in either vaccine.

Pol

The synthetic *pol* gene for both vaccines is from HIV-1 clade B strain NL4-3, and common mutations were introduced in the synthetic protease and reverse transcriptase genes. The protease modification prevents processing of the *pol* gene product, and reduces the potential for functional protease, reverse transcriptase and integrase enzymatic activity. In addition, the *pol* gene in the Ad5-HIV vaccine (VRC-HIVADV014-00-VP) is nonfunctional, because it is present as a fusion protein with the *gag* gene.

Nef

The DNA-HIV vaccine contains a plasmid that encodes for Nef from HIV-1 clade B strain NY5/BRU (LAV-1) recombinant clone pNL4-3. Nef is an accessory protein against which a vigorous T-cell response is mounted in natural infection. Two amino acids in the myristylation site in the HIV-1 *nef* gene were deleted to abrogate MHC class I and CD4+ down-regulation by the Nef protein [38, 39]. There is no *nef* gene included in the rAd5 mixture.

Env A, B and C

The sequences used to create the DNA plasmids encoding Env are derived from three HIV-1 CCR5-tropic strains of virus. These genes have been truncated and modified to improve immunogenicity, which has been demonstrated in mice [79] and monkeys [19]. The clade A Env protein sequence is from strain 92rw020. The clade B Env protein sequence is from strain HXB2 (X4-tropic), which was engineered to replace the region encoding HIV-1 envelope polyprotein amino acids 275 to 361 from X4gp160/h with the corresponding region from the BaL strain (CCR5-tropic). The V1 and V2 loops have been deleted from the clade B *env* gene in the Ad5-HIV vaccine (VRC-HIVADV014-00-VP) to improve stability and yield of the vector in the producer cell line. The clade C Env protein sequence is from strain 97ZA012.

FOR REVIEW ONLY

2.2 DNA vaccine plasmids: VRC-HIVDNA016-00-VP

VRC-HIVDNA016-00-VP is composed of 6 closed, circular DNA plasmids that are each 16.67% (by weight) of the vaccine and are designed to express clade B HIV-1 Gag, Pol and Nef, and HIV-1 Env glycoprotein from clade A, clade B, and clade C. The DNA expression vectors are similar to those used for other candidate vaccines currently undergoing evaluation in clinical studies by the VRC and HVTN. The plasmid and host *E. coli* strain used in the production of the vaccine are characterized in accordance with the relevant sections of the “Points to Consider in the Production and Testing of New Drugs and Biologicals Produced by Recombinant DNA Technology” (1985), the “Supplement: Nucleic Acid Characterization and Genetic Stability” (1992), “Points to Consider in Human Somatic Cell Therapy and Gene Therapy” (1991, 1998), and “Points to Consider on Plasmid DNA Vaccines for Preventive Infectious Disease Indications” (1996).

2.3 Biojector® 2000 Needle-free Injection System

The plasmid DNA vaccine and the PBS placebo will be delivered by the intramuscular route, as this optimally elicits cellular immune responses in animal models [26]. The Biojector® 2000 Needle-Free Injection System will be used for these intramuscular injections. The Biojector uses sterile, single-use syringes that deliver the study material intramuscularly using a compressed carbon dioxide cartridge. The study agent is expelled under pressure through a micro-orifice at high velocity in a fraction of a second.

The Biojector® 2000 Needle-Free Injection System has been approved by the US Food and Drug Administration (FDA) for parenteral administration of medications and immunizations. *In vivo* testing of this system by Bioject, Inc. (Bedminster, New Jersey, USA) has demonstrated effective immune responses with no associated serious adverse experiences. It has been shown to enhance the antibody response to hepatitis A vaccine in humans [80,81] and to HIV DNA vaccines in guinea pigs and rabbits (verbal communication, John Shiver). In studies to evaluate the potential for DNA integration in guinea pigs, it was shown that the extrachromosomal DNA copy number in skin and muscle at 6 weeks was several hundred–fold higher after Biojector delivery than after needle delivery of plasmid [82]. This suggests the potential for more prolonged expression of the transgene, without integration. The Biojector also has been shown to improve the transgene specific T-cell responses to malaria DNA vaccines compared to needle injection in studies in Rhesus macaques and humans [83-86]. A comparative study in humans, VRC008, will commence in Q1 2005 to further investigate the potential benefit that the Biojector may contribute to immunogenicity.

2.4 Recombinant adenoviral vectors: VRC-HIVADV014-00-VP

The recombinant adenoviral vector product VRC-HIVADV014-00-VP (rAd5) is a replication-deficient, combination vaccine containing a mixture of four recombinant serotype 5 adenoviral vectors. The process for constructing the four VRC-HIVADV014-00-VP recombinant adenoviral vectors is based upon a rapid vector construction system (AdFAST™, GenVec, Inc.), which generates four adenoviral vectors, each expressing one of the four HIV antigens gp140(A), gp140(B)dv12, gp140(C) and GagPol(B) driven by the cytomegalovirus (CMV) immediate-early promoter. To construct the adenoviral vector, the HIV-1 DNA sequence was subcloned using standard recombinant DNA techniques into an expression cassette in an E1-shuttle plasmid. Manufacturing is based upon production in a proprietary cell line (293-ORF6), yielding adenoviral vectors that are replication deficient. The vectors are purified using CsCl centrifugation. The product is formulated as a sterile liquid injectable dosage form for intramuscular injection.

FOR REVIEW ONLY

The GV11 adenoviral backbone was chosen to reduce the risk of replication-competent adenovirus (RCA) generation during clinical production. The GV11 backbone contains deletions of two essential regions, E1 and E4, as well as a partial E3 deletion that render the vaccine product replication-deficient. The generation of RCA would require two independent recombination events in a single adenovirus genome, predicted to be an extremely rare event [87]. The Ad_{GV} (HIV).11D vectors contain HIV-1 antigen open reading frame (ORF) expression cassettes inserted to replace the deleted adenovirus E1 gene region. The other deleted adenovirus regions have been replaced with a transcriptionally inert spacer element (T1S1) that enhances production of the adenoviral vectors [88].

The 293-ORF6 cell line used to propagate these E1, E4 and partial E3 deleted vectors was developed at GenVec, Inc. These cells were constructed by stably transforming 293 cells (which are of human embryonic kidney origin) with an inducible E4-ORF6 expression cassette. This enables the cells to efficiently complement the E1-, E4-, and partial E3-deleted adenoviral vectors, provide increased transgene capacity and greatly reduce the potential to generate replication-competent adenovirus. The particular clone that has given rise to the cell line is the A232 clone. The multiclade adenoviral vector vaccine product, VRC-HIVADV014-00-VP, will be a 3:1:1:1 ratio of the adenoviral vectors that encode for HIV-1 Gag/Pol polyprotein from clade B and HIV-1 Env glycoproteins from clades A, B, and C, respectively. Final product meeting all test specifications will be released for use in the proposed clinical study. Vials will be filled to 1.2 mL volume with 1 $x10^{10}$ particle units/mL.

FOR REVIEW ONLY

3 Preclinical studies

3.1 Summary of preclinical safety studies: VRC DNA plasmids

Prior to preparing the Investigational New Drug application (IND) for the 6-plasmid HIV-1 DNA vaccine, VRC-HIVDNA016-00-VP, a request was sent to the FDA for guidance and clarification regarding preclinical safety testing. The response received from the FDA was that the preclinical safety testing done on similar multi-component DNA plasmid products should be sufficient to support the IND for VRC-HIVDNA016-00-VP. The FDA advised that the amount of product administered to study subjects should not exceed that which had been administered in previous DNA vaccine studies (i.e., three vaccinations at the 8 mg dose level).

Preclinical toxicology studies have not been conducted with VRC-HIVDNA016-00-VP. In this section a brief summary of preclinical biodistribution and toxicology studies with a similar 6-plasmid DNA vaccine (VRC-HIVDNA006-00-VP) is provided. Given the high degree of homology for the DNA plasmids, the FDA concurred that preclinical testing of a single combination clade A/B/C product (VRC-HIVDNA006-00-VP) would obviate the necessity for evaluating very similar products to assess toxicological effects, biodistribution, and potential integration. VRC-HIVDNA006-00-VP was not used in a clinical trial, however, the preclinical toxicology and biodistribution studies conducted with this vaccine were used to support the clinical testing of a similar 4-plasmid vaccine, VRC-HIVDNA009-00-VP, and a newer 6-plasmid vaccine, VRC-HIVDNA0016-00-VP which showed a more promising immunogenicity profile in preclinical studies.

TherImmune Research Corporation (Gaithersburg, MD) conducted preclinical safety studies under Good Laboratory Practices (GLP) using a DNA plasmid vaccine VRC-HIVDNA006-00-VP, including a single-dose biodistribution and a repeat-dose toxicity study, using intramuscular injections delivered by a needleless injection system were conducted in New Zealand White rabbits.

3.1.1 Biodistribution of VRC-HIVDNA006-00-VP

In the biodistribution study, evaluation on Day 8 showed that the highest signals were found in the tissues at or adjacent to the injection site. The magnitude of positive signal produced from Day 8 tissues was greatly diminished at each subsequent time point (Day 30 and Day 60), indicating clearance of the test article. All animals survived until the scheduled sacrifice, and no obvious difference in the biodistribution pattern was observed between female and male animals. The biodistribution studies are summarized in more detail in the Investigator's Brochures for the DNA plasmid vaccines VRC-HIVDNA009-00-VP and VRC-HIVDNA016-00-VP.

3.1.2 Repeat-toxicity of VRC-HIVDNA006-00-VP

The toxicity test revealed no apparent test-related alterations in clinical pathology parameters. Although there were some changes observed, they followed no obvious pattern. Therefore, the changes were attributed to individual animal variation in small sample-sized groups. While some organ weight parameters were affected, these alterations could not be verified in the clinical pathology or histology data and therefore were considered incidental.

FOR REVIEW ONLY

More detail on the toxicology studies done with VRC-HIVDNA006-00-VP is provided in the Investigator's Brochures for the DNA plasmid vaccines VRC-HIVDNA009-00-VP and VRC-HIVDNA016-00-VP.

3.2 Summary of preclinical safety studies: VRC rAd and DNA prime followed by rAd boost

The Investigator's Brochures provide more extensive information about the preclinical safety studies for the DNA plasmid vaccine and rAd5 vaccine administered as single agents; the FDA did not require that additional biodistribution studies be performed on the combination regimen.

Gene Logic Inc. (Gaithersburg, MD) conducted a repeated-dose toxicology study of the 4-plasmid DNA vaccine (VRC-HIVDNA009-00-VP) in combination with the rAd5 vaccine (VRC-HIVADV014-00-VP) boost in New Zealand White rabbits under GLP. The DNA was delivered intramuscularly using a Biojector device and the rAd5 vaccine was delivered intramuscularly via needle and syringe. This study determined effects of vaccination on mortality, clinical observations, body weights and changes, food consumption, ophthalmology, immunogenicity, organ weights and ratios, gross & histopathology, clinical chemistries, hematology, and coagulation parameters. More detail on the toxicology studies performed with rAd5 vaccine administered alone, and as part of a prime-boost combination is provided in the Investigator's Brochure for the rAd5 vaccine VRC-HIVADV014-00-VP.

3.2.1 Repeat-toxicity of DNA prime/rAd5 boost HIV vaccine regimen

DNA vaccine and PSB control were administered 4 times (study day 1, 22, 43, and 64), and adenoviral vector vaccine and diluent control were administered twice (study day 85 and 106). In this study, all animals survived to sacrifice and necropsy. In the adenoviral vector alone group, no treatment-related observations were made with regard to morbidity/clinical observations, body weights and changes, and ophthalmology. No prime-boost treatment-related observations were made with regard to morbidity/clinical observations and ophthalmology. However, possible prime-boost treatment effects were seen with body weights and changes, particularly in treated females. Differences began to be noted as early as study day 36, but became statistically significantly different from control females on study days 71, 78, 92, 99, and 108 for body weights and days 85-92 for body weight changes in prime-boost treated females. These animals continued to gain weight over the course of the study, but did not gain as much weight as the controls.

Additionally, minimal erythema was seen at the injection sites in a couple of treated males and one control female after the second adenoviral vector injection when given alone. In contrast, in the prime-boost regimen, vaccination with the DNA prime resulted in Draize observations of minimal to moderate edema and erythema in a few treated animals, increasing in frequency and severity with repeated dosing. This was a result of the combination of injection with the Biojector and the active vaccination, as these observations also occurred in the control animals but to a lower amount and lesser degree. These findings were consistent with toxicology studies performed with the DNA vaccination alone. Boost (adenoviral vector delivered by needle and syringe) injections did not increase the frequency or severity of the Draize observations seen at earlier time points (after priming doses).

Clear treatment-related (adenoviral vector alone and prime-boost) observations were seen in gross and histopathology at the injection sites and in the histopathological findings of inflammation in the perineural tissue of the sciatic nerve (near the injection site). These latter lesions consisted of chronic inflammatory cells (small macrophages and lymphocytes) in the

FOR REVIEW ONLY

connective tissue around the sciatic nerve and in adjacent lymphatics and blood capillaries. This inflammation was likely the result of the distal injection sites with drainage toward proximal lymph nodes. The injection site reactions were less frequent and less severe in the recovery sacrifice animals than in the immediate sacrifice animals for both the adenoviral vector alone and the prime-boost regimens, demonstrating the reversibility of the injection site reactions.

Fever was seen in the 24 hours subsequent to the initial, but not second, adenoviral vector vaccination (adenoviral vector only arm), and was more striking in treated males than females. These fevers resolved by 48 hours. Likewise, fever was seen in treated males and females in the 24 hours subsequent to the initial, but only in the first 3 hours after the second adenoviral vector boost (only in treated females), in the prime-boost treated animals. These fevers resolved by 48 hours after the initial and 24 hours after the second (treated females only) adenoviral vector boost.

Food consumption in the rabbits was less in the 24 hours (adenoviral vector alone and prime-boost) to 48 hours (prime-boost) following each adenoviral vector vaccination, but resolved, and did not result in differences in body weights or changes in males or females inoculated with adenoviral vector alone or treated males in the prime-boost regimen. However, it did result in the noted body weight changes in treated females in the prime-boost regimen (although these differences began prior to exposure to adenoviral vector as discussed above).

There were many other observations, particularly in clinical chemistries and hematology parameters, which were unclear in their relationship to treatment because they either remained within the normal range for the species and laboratory (even though there were statistically significant differences from matched control animals on study) or they were outside the normal range and different from the control animals on study but were not consistent between genders or across time points. None of these findings correlated with clinical observations or gross or histopathological findings. Of note among these, however, was the finding of statistically significant (from matched controls on study) elevated triglycerides on the second day subsequent to the initial adenoviral vector inoculation in both treated males (mean was 1.5 times the upper limit of normal - ULN) and females (mean was >1 but <1.5 time the ULN) receiving adenoviral vector vaccination alone and on the day subsequent to the initial adenoviral vector boost in treated males (mean was between 3-4 times the ULN), but not treated females, receiving the prime-boost regimen.

3.3 Summary of preclinical immunogenicity studies

Although there are no animal models of HIV-1 infection that is highly predictive of what will be seen with vaccination in human clinical trials, immunogenicity studies are typically carried out in mice and monkeys, and challenge studies are often carried out in macaques with the SIV analogues of the candidate HIV-1 vaccines. As shown in the Table 3-1 below, preclinical immunogenicity studies were conducted in mice, rabbits and non-human primates with several of the VRC-HIV DNA and Ad5-vectored vaccines at the VRC and Beth Israel Deaconess Medical Center, Harvard Medical School (Boston, MA). The immunogenicity studies in mice and rabbits, as well as the non-human primate studies VRC-02-035 and VRC-03-060 were conducted using materials from the same exact bulk lots as those intended for clinical trials and formulated exactly as clinical trial material.

In summary, the studies shown below indicate that the previous generation of VRC DNA and Ad5 vaccines was able to induce partial protection from challenge in the SHIV model system, and that the improvements made with the current generation of both VRC candidate HIV vaccines (VRC-HIVDNA-016-00-VP and VRC-HIVADV-014-00-VP) can reasonably be

FOR REVIEW ONLY

expected to induce immune responses of greater magnitude and breadth than those from the earlier constructs. More detail on these studies can be found in the Investigator's Brochures.

FOR REVIEW ONLY

Table 3-1 Summary of preclinical immunogenicity studies

Test System	Dose	Treatments per Animal	Treatment Period	Study Duration	Conclusions	References
Mouse	rAd: 1 x 10^{11}PU	1	3 wks	4 wks	VRC-HIVADV014-00-VP immunization elicited humoral and cellular immune responses in mice.	Summary in VRC-HIVADV014-00-VP IB Section 5.3.1
Mouse	DNA: VRC-HIVDNA016-00-VP, 50µg	1	0 day	3 wks	Vaccination with the CMV/R plasmid encoding gag-pol-nef fusion protein (contained in VRC-HIVDNA016-00-VP vaccine product) elicits higher HIV-1specific cellular immune responses in mice than the unmodified 1012 plasmid encoding the same fusion protein (contained in VRC-HIVDNA009-00-VP vaccine product).	Summary in VRC-HIVDNA016-00-VP IB Section 4.2.1
Rabbit	DNA: VRC-HIVDNA009-00-VP, 4 mg<br><br>rAd: 1 x 10^{11} PU	6	106 days	119 days	Immunization VRC-HIVDNA009-00-VP DNA prime followed by VRC-HIVADV014-00-VP boost elicited humoral immune responses in rabbits.	Gene Logic (formerly TherImmune) Repeated Dose Toxicity Study #1195-114 and Summary in VRC-HIVADV014-00-VP IB Section 5.3.2
Rhesus macaques	DNA: (SIV)<br>10 mg<br><br>rAd: 2 x 10^{12} PU	3 DNA + 1 rAd	0, 4, 8 (DNA) + 26 wks (rAd)	64 wks	Immunization with SIVmac239 *gag-pol-nef* + (89.6P or HXB2 BaL *env* DNA prime/rAd (no *nef*) boost confers partial protection against SHIV-89.6 challenge. Matched (89.6P) or mismatched (HXB2 BaL) Env immunogens conferred better immunity than vaccination without Env.	Study ASP-015 Letvin, et al. Virology (2004) 78:7490-7
Rhesus macaques	DNA: (SIV)<br>9 mg<br><br>rAd: 1 x 10^{12} PU	3 DNA + 1 rAd	0, 4, 8 wks (DNA) + 26 wks (rAd)	42 wks	Immunization with a four component multiclade vaccine resulted in broader responses without loss of immunogenicity to any component as compared with the vaccines consisting of plasmids and rAd expressing SIV Gag/Pol-(Nef) and HIV-1 Env from a single clade.	Study VRC-026A Summary in VRC-HIVADV014-00-VP IB Section 5.4.1
Cynomolgus macaques	DNA: 4-plasmid VRC-HIVDNA009-00-VP or 6-plasmid HIV-DNA016-00-VP, 8 mg<br><br>rAd:VRC-HIVADV014-00-VP 1 x 10^{11} PU	3 DNA<br>1 rAd	0, 4, 8 wks (DNA) + 38 wks (4-plasmid rAd boost) or 24 wks (6-plasmid rAd boost)	58 wks	Cynomolgus macaques receiving DNA prime/rAd boost immunization with the 6-plasmid DNA vaccine that expresses HIV-1 Gag, Pol, Nef and clade A, B and C Env (VRC-HIVDNA016-00-VP), and boosted with rAd expressing HIV-1 Gag/Pol and 3 Env, elicited cellular immune responses to all vaccine antigens. The 6-plasmid prime appeared t o be more immunogenic than the 4-plasmid DNA vaccine prime.	Study VRC-02-035 Summary in VRC-HIVDNA016-00-VP IB Section 2.3.2

FOR REVIEW ONLY

4 Clinical studies

4.1 DNA plasmid vaccine trials

Human trials conducted with other plasmid DNA vaccines using similar clinical products have revealed no significant toxicity, autoimmunity, or other adverse reactions [14,17,27]. The status of completed and ongoing Phase I VRC, HVTN, and USMHRP studies with HIV-1 plasmid DNA vaccines developed by the VRC are summarized in Table 4-1 and briefly described below:

Table 4-1 Multiclade HIV DNA vaccine experience in uninfected participants as of May 2005

HIV DNA vaccine formulation	Study	Dose (mg)	# active doses planned (participants in active arm)	# active doses to date (participants in active arm)	Comment
**VRC-HIVDNA009-00-VP**<br>4 plasmids (multiclade):<br>clade B *gag-pol-nef,*<br>clade A *env*,<br>clade B *env*,<br>clade C *env*.	VRC 004	2.0<br>4.0<br>8.0	15 (5)<br>60 (20)<br>45 (15)	15 (5)<br>60 (20)<br>44 (15)	Vaccinations completed and study unblinded;<br>also included 10 placebo participants (29 placebo injections)
	HVTN 052	4.0	300 (120)	≥ 292 (120)	Of 540 blinded doses (300 active and 240 placebo) planned, 8 total were not given (still blinded).
	RV 156	4.0	45 (15)	~30 (15)	Enrollment initiated in January 2005. Study also includes 15 placebo participants (45 planned placebo injections)
**VRC-HIVDNA016-00-VP**<br>6 plasmids (multiclade):<br>clade B *gag,*<br>clade B *pol*,<br>clade B *nef*,<br>clade A *env*,<br>clade B *env*,<br>clade C *env*.	VRC 007	4.0	45 (15)	44 (15)	Vaccinations completed; open label; no placebos.
	VRC 008	4.0	120 (40)	0	Initiation May 2005
Summary: A dose range of 2.0 mg to 8.0 mg was evaluated for the 4-plasmid DNA vaccine. The majority of experience is with the 4.0 mg dose. This dose range was well tolerated.<br>Phase I studies (uninfected participants) include plans for 630 vaccine injections in 230 participants.<br>As of May 5, 2005, 190 uninfected participants have received one or more vaccine injections.					
Note: Experience with a clade B single plasmid vaccine, with the 4-plasmid DNA vaccine in combination with an IL2/Ig adjuvant, and with the 4-plasmid DNA vaccine in HIV-infected participants is not shown. These studies together include more than 50 vaccinees to date.					

4.1.1 Protocol VRC 001

The VRC completed a Phase I, randomized, controlled, double-blinded dose escalation study, VRC 001 (BB-IND 9782), to evaluate safety, tolerability, dose and immune response of an HIV plasmid DNA vaccine expressing a clade B Gag-Pol fusion protein (pGag (del fs)

FOR REVIEW ONLY

PolΔPRΔRTΔIN/h; VRC-4302), which is similar in composition to VRC-HIVDNA009-00-VP and VRC-HIVDNA016-00-VP. Each of three groups of seven healthy, HIV-negative volunteers received a constant dose of the vaccine (5 people) or a phosphate buffered saline (PBS) control (2 people) by intramuscular inoculation. Once safety was established, successive groups received a higher dose. Study groups received three immunizations containing 0.5 mg (Group 1), 1.5 mg (Group 2), or 4.0 mg (Group 3) of the DNA vaccine or placebo. All 21 participants received all planned injections, which were well tolerated. All participants completed the study. There were no serious adverse events attributed to the study agent.

4.1.2 Protocol VRC 004

Protocol VRC-004 (03-I-0022) is a Phase I randomized, controlled, double-blinded dose escalation study to evaluate safety, tolerability, dose and immune response of a multiclade HIV plasmid DNA vaccine identified as VRC-HIVDNA009-00-VP. This study opened to accrual in November 2002 and was fully enrolled with 50 healthy HIV-negative participants in August 2003. Forty participants received vaccine injections and 10 participants received placebo injections. The three-injection schedule (administered at Weeks 0, 4 and 8), was completed in 5 of 5 participants randomized to 2 mg vaccine injections, 20 of 20 participants randomized to 4 mg vaccine injections, 14 of 15 participants randomized to 8 mg vaccine injections and 9 of 10 participants randomized to placebo injections. The unblinded final study results indicate that the vaccine injections were as well tolerated as the placebo injections.

In the vaccine groups, there were three adverse events possibly related to vaccine that required expedited reporting to the IND sponsor. These were a grade 3 asymptomatic neutropenia with onset 27 days after 3rd vaccination (4 mg group), a grade 3 urticaria with onset 4 days after 3rd vaccination (4 mg group) and a grade 2 maculopapular rash with onset 27 days after 2nd vaccination (8 mg group). All resolved without sequelae. Other factors in the occurrence of the urticaria include concomitant bladder infection, yeast infection and multiple antibiotics. The rash resulted in discontinuation from the vaccination schedule after the 2nd injection and it was clinically consistent with either a drug eruption or a viral exanthem. The diary cards indicate that vaccine injections were well tolerated. No participants reported severe symptoms on diary cards. Most participants (80-100% per group), including placebo recipients, reported at least one local symptom (pain/tenderness, induration or erythema) at some point in the 7 days after an injection. Most participants (70-80% per group), including placebo recipients, also reported at least one systemic symptom in the 7 days after an injection. No vaccine recipients reported fever. Chills and nausea were infrequent in all participants (0-20% per dose group). Headache and myalgia were reported in 20-50% per dose group. Malaise was the most common systemic symptom, occurring in 50-60% of vaccinees and 40% of placebo recipients at least once in the 7 days following a study injection. During the study the most frequently recorded laboratory adverse events included asymptomatic hyperglycemia and hypoglycemia. The unblinded data show that placebo recipients had higher incidence of both hyperglycemia and hypoglycemia and these data support the clinical impression that variations in blood glucose are unrelated to study vaccinations.

Preliminary immunogenicity data through Week 12 from the VRC 004 study, when sorted by treatment assignment indicate that $CD4^+$ responses were detected in nearly 100% of recipients at all dose levels. $CD8^+$ responses were detected in nearly half. The greatest responses (in frequency and magnitude) were generally observed as directed against Env. The immunogenicity response to the Gag, Pol and Nef are weak to absent. There is a trend to

FOR REVIEW ONLY

greater responses in the 4 mg and 8 mg dose compared to the 2 mg dose, although not statistically significant. There was a statistically significant increase in the response after 3 injections compared to 2 injections at both the 4 mg and 8 mg dose levels, although there is no way to determine if this was due to the 3rd injection or simply a maturation of the response following the 2nd injection. Definitive responses are first detectable with the 4 mg and 8 mg dose at the 6-week time point (2 weeks after the second injection). When compared with the 8 mg dose, the 4 mg dose offers the combination of a good safety profile, greater ease of administration, and approximately equivalent cellular immunogenicity.

Fourteen of 35 vaccinees in the 4 mg and 8 mg groups had a positive ELISA at one or more points between Week 8 and Week 52 when tested by a commercial HIV antibody test. The optical density (O.D.) of the ELISA results usually decreased over time; six participants were ELISA positive by the commercial assay at Week 52.

Some VRC 004 participants may be offered the opportunity to enroll in a separate study (VRC 009) in which they could receive a single boost injection of VRC-HIVADV014-00-VP, a VRC recombinant adenoviral vector vaccine, at 1 x 10^{10} PU. That study will provide safety data of the adenoviral vector vaccine as a booster vaccine and will complement the immunogenicity data obtained in HVTN 057.

4.1.3 Protocol HVTN 044

Protocol HVTN 044 (BB-IND 10914) is a placebo-controlled Phase I study to evaluate the safety and immunogenicity of the 4 mg dose of the 4 plasmid multiclade VRC-HIVDNA009-00-VP vaccine administered in combination with escalating doses (0.1, 0.5, 1.5 and 4.0 mg) of a plasmid cytokine adjuvant VRC-ADJDNA004-IL2-VP (IL-2/Ig) in 70 HIV-negative participants. For this study, the multiclade DNA vaccine is being administered using the Biojector apparatus. The study opened for enrollment in December 2003, but is still accruing due to the multiple built-in safety pauses for the dose escalation of the adjuvant. As of March 2005, 54 out of 70 participants have been enrolled into this trial, 38 of which could have received the multiclade VRC-HIVDNA009-00-VP vaccine. All enrolled participants have received the first injection, 39 have received the 2nd injection, 31 have received the 3rd injection, and 17 have received all 4 injections.

Local reactions to the study vaccine, including mild or moderate pain and/or tenderness at the injection site, were reported by 47 participants (81% of these were mild in severity). Mild erythema and/or induration were reported by 21 participants. Systemic reactogenicity symptoms (i.e., malaise, myalgia, headache, nausea, vomiting, chills or arthralgia) were experienced by 34 participants. The vast majority (79%) of these were mild in severity. There was one report of severe (grade 3) malaise which is discussed in greater detail below.

There were two grade 3 events possibly related to vaccine that required expedited reporting to the IND sponsor. One participant developed a grade 3 decrease in CD4 count that was considered possibly related to the study vaccine. This participant was enrolled in the study with a preexisting grade 2 CD4 lymphopenia, and is currently being worked up for a possible diagnosis of sarcoidosis. If confirmed, sarcoidosis would be the likely contributor to the low CD4 counts. The HVTN Safety Monitoring Board reviewed this event at the time of its occurrence in an unblinded fashion and determined that it was safe to continue the study. Another participant developed grade 3 severe malaise 1 day after the 3rd vaccination. These symptoms resolved by day 3 and further vaccinations were discontinued for this participant. This event was also reviewed in an unblinded fashion by the HVTN Safety Monitoring Board, which determined that it was safe to proceed with the study.

FOR REVIEW ONLY

4.1.4 Protocol HVTN 052

The HVTN is conducting a Phase IB study, HVTN 052 (BB-IND 10681), to evaluate the safety and immunogenicity of the 4-plasmid VRC-HIVDNA009-00-VP in a two- versus a three-injection regimen in 180 participants (120 vaccine/60 control). The schedule being compared is 0, 4 and 8 weeks versus 0 and 8 weeks, and all injections are given by Biojector. The study opened for enrollment in December 2003 and accrual was completed on October 19, 2004. As of May 2005, all 180 participants are beyond Week 8; 8 of the 540 planned injections were not administered. The study remains blinded.

Local reactions of pain and/or tenderness at the injection site were reported by 158 (88%) of the participants. Mild erythema and/or induration were reported by 36% of the participants. All local reactions were mild or moderate, except in one participant, who experienced an episode of severe injection site pain that started 30 minutes after the first vaccination. The pain was mild by the following day and resolved by day 4.

Sixty percent of the participants experienced mild or moderate symptoms of systemic reactogenicity (malaise, myalgia, headache, nausea, vomiting, chills or arthralgia), the vast majority of which (83%) were mild.

As of February 2005, an unblinded review of HVTN 052 safety data by the HVTN Safety Monitoring Board indicated that there were no significant differences in AEs or SAEs across treatment groups. As of March 2005, there have been three adverse events that required expedited reporting to the IND sponsor, excluding those ultimately deemed not related. One participant experienced severe injection site pain that was mild by the day after vaccination as discussed above. A 41-year-old male participant developed an increased glucose (261 mg/dl) at visit 5, 13 days after the 2nd vaccination. All other laboratory values were within normal limits at this visit. The participant had a family history of diabetes mellitus and a BMI of 35.7 and reported eating a very large breakfast, as well as soda and cookies, prior to the blood draw. This event was ultimately attributed to a new diagnosis of diabetes mellitus and determined unlikely to be related to vaccination. One 44-year-old male participant developed grade 3 platelet elevation. The participant had pre-enrollment platelet counts that met the inclusion criterion but were somewhat above the site’s upper limit of normal. The participant is asymptomatic and continues protocol visits, during which the elevations have persisted.

4.1.5 Protocol RV 156

RV 156 is a Phase I clinical trial to evaluate the safety and immunogenicity of the multiclade HIV-1 DNA plasmid vaccine, VRC-HIVDNA009-00-VP, in uninfected adult volunteers in Uganda. The study is fully enrolled with 30 participants, half of whom receive three 4 mg doses of the 4-plasmid DNA vaccine and half of whom receive placebo. As of March 25, 2005, no notable vaccine-associated adverse events had been identified.

4.1.6 Protocol VRC 007

VRC 007 (04-I-0254) is the first Phase I study of the 6-plasmid DNA vaccine, VRC-HIVDNA016-00-VP. This open-label study enrolled 15 participants between August 17, 2004 and October 28, 2004. Fourteen of the 15 participants received 3 intramuscular injections of a 4 mg dose of vaccine administered by Biojector; one participant was lost to follow-up after two vaccinations. The last study vaccination was administered on December 22, 2004. This summary represents interim results through May 4, 2005. No participants reported fever following vaccination. Reactogenicity, as reported on 43 diary cards, was none to mild except that two participants reported moderate injection site pain and one participant reported moderate nausea and malaise. The only adverse event requiring expedited reporting

FOR REVIEW ONLY

to the IND sponsor was a grade 3 generalized urticaria. The participant had reported starting an antihistamine about 2 weeks after first vaccination but reported at that time that the reason was latex allergy. While being screened for the rollover boost study, VRC 010, it was learned that the subject had experienced generalized urticaria around the time of the second vaccination when the supply of antihistamine ran out. As of May 2005 the participant has chronic urticaria that are well controlled by antihistamine. Evaluation is ongoing. The etiology is unknown, but at this time the chronic urticaria are assessed as possibly related to study vaccine. As of May 2005, there have been two moderate (grade 2) adverse events possibly attributed to vaccine. These were intermittent dizziness of 2 days duration beginning 13 days after the second vaccination in one participant (this participant received the third vaccination without recurrence of symptoms) and asymptomatic hypoglycemia in another participant, first noted at the follow-up visit that was 14 days after the third vaccination. Fourteen participants remain on study. The last safety evaluation of the participant lost to follow-up was by telephone one day after the second vaccination; at that time the participant reported no side effects from the vaccination.

An unexpected local injection site reaction for this DNA vaccine has been observed. Mild cutaneous lesions (0.5-1.0 cm diameter) at the vaccination site occurred after 4 of 44 (9%) vaccinations administered; these occurred in 3 of 15 (20%) participants. Participants were routinely asked to call if they experience any unusual problem after study vaccinations. The vaccination site cutaneous lesions did not alarm participants enough to prompt them to contact the VRC Clinic prior to their next regularly-scheduled visit. In retrospect, three participants reported that they experienced skin lesions that started as a small papule or vesicle within 3 days after vaccination. After a few days the papule or vesicle unroofed and a scab formed. There was surrounding mild erythema and mild induration. After the scab came off, the skin healed without treatment. None of the cutaneous lesions were associated with pustular exudates, fever, rash or urticaria. They did not appear to be either a local infection or allergic reaction.

The first three cutaneous lesions were discovered at the first post-vaccination clinic visit (days 14 ± 3 Day); at that time they were largely resolved. The fourth cutaneous lesion, was examined in the clinic while still in an active stage, and it was biopsied at post-vaccination day 6. This biopsy demonstrated a microscopic subcutaneous and dermal perivascular lymphocytic infiltrate. The infiltrate was composed almost exclusively of CD3 positive cells, including both $CD4^+$ and $CD8^+$ T cells. There were rare eosinophils present and rare giant cells noted. The process appeared to be primarily a subcutaneous and dermal response to vaccination with cutaneous manifestations.

The reason these reactions have been seen in VRC 007 and not studies evaluating other DNA vaccines delivered intramuscularly by Biojector is not known. Whether these reactions correlate with the strength of the vaccine-induced immune response also is not yet known. Eight of the 14 participants remaining on study have had a vaccine-induced positive HIV ELISA by a commercial test at one or more time points; this includes all 3 participants who had a cutaneous lesion. Preliminary immunogenicity data from VRC 007 (6-plasmid DNA product) suggests the Env-specific T cell responses are similar to those seen in VRC 004 (4-plasmid DNA product), and now Gag- and Nef-specific responses also are present.

4.2 Recombinant adenoviral vector vaccine trials

The status of completed and ongoing Phase I VRC and HVTN studies with the HIV-1 adenoviral vector vaccine developed by the VRC are summarized in the Table 4-2 and briefly described below:

FOR REVIEW ONLY

Table 4-2 Multiclade HIV adenoviral vaccine, VRC-HIVADV014-00-VP, experience in uninfected participants as of May 2005

Ad vaccine formulation	Study	Single dose (PU)	# active doses planned	# active doses to date	Comment
**VRC-HIVADV014-00-VP** 4 adenoviral vectors with inserted plasmids (multiclade): clade B *gag-pol* clade A *env*, clade B *env*, clade C *env*	VRC 006	10^9 10^{10} 10^{11}	10 10 10	10 10 10	Vaccinations completed. Study includes 6 placebo participants.
	VRC 008	10^9 10^{10}	20 20	0 0	Initiation May 2005
	VRC 009	10^{10}	≤ 32	8	First enrolled in January 2005
	VRC 010	10^{10}	≤ 14	1	First enrolled in May 2005
	HVTN 054	10^{10} 10^{11}	20 20	≤ 5 0	First enrolled in April 2005. Study includes 8 placebo participants.
	HVTN 057	10^{10}	60	60	Vaccinations completed. Study includes 10 placebo participants.
Summary: A dose range of 10^9 PU to 10^{11} PU has been evaluated in Phase I studies. The majority of experience is with the 10^{10} PU dose. Reactogenicity increases with higher doses, but the full dose range is well-tolerated. The experience as of May 2005 includes approximately 94 injections of the 10^{10} PU dose and 10 injections of the 10^{11} PU dose.					

4.2.1 Protocol VRC 006

The Vaccine Research Center (VRC) is conducting VRC 006 (04-I-0172), "A Phase I Clinical Trial to Evaluate the Safety and Immunogenicity of a Recombinant Multiclade HIV-1 Adenoviral Vector Vaccine, VRC-HIVADV014-00-VP, in Uninfected Adult Volunteers." This is a randomized, placebo-controlled, double-blinded, dose escalation study to examine safety, tolerability and immune response following a single injection of VRC-HIVADV014-00-VP at a dose of 10^9 PU, 10^{10} PU, or 10^{11} PU. Each group includes 12 participants (10 vaccine; 2 placebo). VRC 006 was initiated on July 19, 2004 and the study completed enrollment of 36 participants on November 10, 2004. The NIAID Intramural Data and Safety Monitoring Board (DSMB) reviewed the preliminary safety data through 14 days of follow-up prior to each dose escalation. The preliminary data indicate that the vaccine appears to be safe for healthy participants at the three dose levels evaluated. The 10^9 and 10^{10} PU dose levels are associated with less reactogenicity than the 10^{11} PU dose level. In both the 10^9 and 10^{10} PU dose groups the local and systemic parameters recorded on the 5-day diary card were none to mild in severity and none of the participants experienced fever. In the 10^{11} PU dose group, four participants reported fever on Day 1 (3 mild and 1 moderate in severity). Each of the four participants with fever also reported moderate headache on Day 1 and three of these participants also reported at least one other moderate systemic parameter (malaise, myalgia, chills). Two participants without fever reported at least one moderate systemic symptom (malaise, myalgia, nausea). One participant in the 10^{11} PU dose group reported moderate injection site pain; injection site reactogenicity was otherwise none or mild.

As of April 27, 2005, there has been one grade 4 (potentially life-threatening) and three grade 2 (moderate) adverse events that are possibly related to vaccination. The study remains blinded to vaccine vs. placebo injection assignments. The grade 4 adverse event was a seizure that occurred 64 days after study injection in a healthy participant in the 10^{11} PU dose group who had a history of a single seizure three years prior to study enrollment. Following the

FOR REVIEW ONLY

review of past medical records and test results and given the history of a prior seizure and the timing of the event more than 2 months after study injection, the seizure was assessed as unrelated to study agent. The grade 2 adverse events possibly related to study agent include: 1) asymptomatic neutropenia noted 21 days after study injection in a participant known to sometimes have asymptomatic low neutrophil counts prior to enrollment; 2) diarrhea (duration one day) in a different participant on the third day after study injection and 3) steatohepatitis (fatty liver) diagnosed after extensive evaluation to identify the cause of a persistent grade 1 ALT (alanine aminotransferase) elevation that was noted starting 25 days after the study vaccination in a clinically asymptomatic participant. A hepatology consultant reported an impression that the condition likely existed prior to study enrollment. Contributing factors to the persistent grade 1 ALT may be alcohol consumption and recent weight gain. A diagnosis of steatohepatitis is overall considered to be a grade 2 condition, but the liver function tests remained at grade 1 severity for about 5 months and then was within normal range at last study visit; a repeat ultrasound showed fatty liver was still present.

Although more reactogenicity has been observed with the 10^{11} PU dose, it appears to be a well-tolerated dose and analgesic/antipyretic nonprescription medications may be self administered for relief of the short-term symptoms. The interim immunogenicity data suggest a dose effect with increasing immune response at higher doses. The number of participants with vaccine-induced ELISA at study week 12 by commercial HIV-antibody assay increases from 3 in the 10^9 PU group, to 6 in the 10^{10} PU group, and to 9 in the 10^{11} PU group among the 12 participants (two placebo and ten vaccine recipients) per group. Preliminary immunogenicity data from VRC 006 suggest the majority of vaccinees develop both CD4+ and CD8+ Env, Gag, and Pol specific T cell responses.

4.2.2 Protocol HVTN 054

HVTN 054 is the second Phase I study of the rAd vaccine, VRC-HIVADV014, as a single agent in uninfected adult participants. This randomized, placebo-controlled, dose escalation study was submitted to BB-IND 11661 in December 2004 and opened to accrual in April 2005. It is designed to enroll two groups of 24 participants with low Ad5 neutralizing antibody titer (<1:12) that will be randomized to rAd or placebo in a 5:1 ratio. The first group of vaccinees will receive 10^{10} PU rAd and the second group will receive 10^{11} PU rAd.

4.3 Prime-boost trials

4.3.1 Protocol HVTN 057

HVTN 057 (BB-IND 11894) is the first Phase I study to administer the adenoviral vector vaccine, VRC-HIVADV014-00-VP, as a booster vaccination. In this blinded Phase I study a single boost at 10^{10} PU (or a placebo) is administered to participants who completed the injection regimen with VRC-HIVDNA009-00-VP or placebo in HVTN 052. The rAd boost is given at an interval of 6-9 months after the participant’s first injection in HVTN 052. The first participant was enrolled on November 22, 2004, and on April 20, 2005 the last enrollment and study injection was completed.

As of May 13, 2005 the still blinded reactogenicity results indicated local pain and/or tenderness reported by 55 (78.6%) participants, with maximal severity moderate, reported for 3 (4.2%) participants. Erythema and/or induration no greater than 25 cm^2 was reported for 10 (14.3%) participants, of whom 7 (10.0%) reported the erythema and/or induration as <10 cm^2. Systemic reactogenicity did not exceed moderate: 18 (25.7%) reported mild symptoms and 6 (8.5%) reported moderate symptoms. The most commonly reported mild symptoms were malaise and/or fatigue in 20 (28.5%) participants, myalgia in 15 (21.4%) participants, and

FOR REVIEW ONLY

headache in 10 (14.2%) participants. The most commonly reported moderate symptoms were headache in 7 (10.0%) participants, malaise and/or fatigue in 6 (8.5%) participants, and myalgia in 5 (7.5%) participants. Six (8.5%) participants reported grade 1 fever, none of which exceeded 38.5 ^{o}C.

4.3.2 Protocol VRC 009

VRC 009 is a Phase I study of rAd as a booster vaccine. It is an open label study designed to enroll subjects who completed three vaccinations with 4 mg or 8 mg of VRC-HIVDNA009-00-VP in VRC 004 (03-I-0022) to receive a 10^{10} PU rAd booster vaccination. The first enrollment into this study occurred January 28, 2005 and as of May 5, 2005 eight subjects were enrolled; four from the 4 mg group and four from the 8 mg group in VRC 004. The mean boost interval to date is 91 weeks [range 79-104 weeks] from first DNA prime vaccination. There have been no serious adverse events. All 8 subjects had mild pain at the injection site and 5 of 8 subjects had at least one mild or moderate symptom (malaise, myalgia, headache or chills). Seven of the 8 subjects have reached the first HIV ELISA testing time point and all have shown a vaccine-induced antibody by the commercial ELISA test method.

4.3.3 Protocol VRC 008

VRC 008 is a Phase I randomized study to examine safety and tolerability of and immune response to a prime-boost vaccination schedule. The schedule includes three doses of the 6-plasmid multiclade DNA-HIV vaccine, VRC-HIVDNA016-00-VP, at weeks 0, 4 and 8, followed by one dose of the multiclade HIV adenoviral vector vaccine, VRC-HIVADV014-00-VP, booster at week 24. Forty participants, half with high (>1:500) and half with low (≤1:500) adenovirus serotype 5 neutralizing antibody titers, will be randomized in a 1:1 ratio to receive the DNA vaccinations by either needle and syringe or by Biojector and also randomized in a 1:1 ratio to receive the booster vaccination with either 10^{10} PU or 10^{11} PU of the adenoviral vector vaccine. The boost dose is blinded, but there are no placebo injections. This study opened to accrual in May 2005.

4.3.4 Protocol VRC 010

VRC 008 and VRC 010 (for VRC 007 rollover subjects) together (BB-IND 12326) will provide the Phase I safety and immunogenicity data for the prime-boost regimen that uses the 6-plasmid DNA vaccine, VRC-HIVDNA016-00-VP, for the priming vaccinations and VRC-HIVADV014-00-VP for the booster vaccination. The first rAd booster injection (10^{10} PU) of a subject primed with the 6-plasmid DNA vaccine occurred on May 4, 2005 when the first subject was enrolled into VRC 010.

FOR REVIEW ONLY

5 Summary

5.1 Rationale for trial design

This Phase II study will investigate in a diverse population of study volunteers the safety and immunogenicity of a multiclade DNA plasmid prime followed by a multiclade, multivalent replication-incompetent recombinant adenoviral vector boost. Similar multi-clade, multivalent HIV inserts were used for these two vaccines to maximize the effect of priming and the breadth of the immune response, and to date studies show that the combination of vaccines similar to these are safe and well-tolerated. This protocol is on the critical path to qualify this vaccine combination for efficacy evaluation, and is intended to harmonize with two other studies currently in development, RV 172 and IAVI V001, which will be carried out in eastern Africa, respectively, by the US Military HIV Research Program (USMHRP) and the International AIDS Vaccine Initiative (IAVI).

HVTN 204, RV 172 and IAVI-V001 are designed to generate comparable safety and immunogenicity data from three distinct geographic regions, the Americas (HVTN 204), southern Africa (HVTN 204) and eastern Africa (RV172 and IAVI V001). As the Ad-5 seroprevalence in these regions is not yet precisely understood, the regional approach is being pursued to garner the safety and immunogenicity data that would be required to move the vaccination regimen forward in advanced phase clinical trials in one or more of the regions independently. In each region, 3 doses of 4 mg of the VRC DNA-HIV vaccine (VRC-HIVDNA016-00-VP) followed by a single 1 x 10^{10} PU dose of VRC Ad5-HIV vaccine (VRC-HIVADV014-00-VP) will be given to approximately 120 participants in identical vaccination schedules. In each region, an equivalent number (N≈120) of control vaccine recipients will be enrolled as well. In an attempt to assure that the variety of HIV transmission modes are represented among participants in HVTN 204, our goal is that at least 40% of participants will be male, in whom the predominant mode of transmission is assumed to be MSM contact in the Americas and heterosexual contact in southern Africa, and at least 40% of participants will be female, in whom the predominant mode of transmission is assumed to be heterosexual contact in both regions. Gender equity will also be the goal of the eastern Africa studies (RV 172 and IAVI V001).

HVTN 204 is powered to detect fairly small differences in safety and immunogenicity between the vaccine and placebo arms, and the three studies HVTN 204, RV 172, and IAVI V001 assessed together have excellent power to detect fairly small differences in safety and immunogenicity between the vaccine and placebo arms (see Section 9.4.3 for the minimum detectable effect sizes with high power). Taken together these studies should provide sufficient data to qualify the vaccine regimen for expanded studies of vaccine efficacy in these regions.

As pointed out earlier in this document, the relatively high prevalence of adenovirus type 5 immunity worldwide could attenuate the immune response to this adenoviral vector vaccine, and thus limit its usefulness as a global vaccine for adults. The current protocol is designed to evaluate the relationship between preexisting titers of neutralizing antibody to adenovirus 5 and immunogenicity of the combination vaccine regimen. In each region, the populations' existing background Ad5 seropositivity is being elucidated, and although in HVTN 204, RV 172, and IAVI V001 study participants will be enrolled without regard to preexisting Ad5 immunity (i.e., no Ad5 neutralizing antibody titer prescreening and stratification), the preexisting Ad5 neutralizing antibody titers will be determined and considered in the secondary analysis of the safety and immunogenicity data. Specifically, assessment of safety

FOR REVIEW ONLY

and vaccine take rate among subsets of participants with lower levels of Ad5 neutralizing antibody titer will be performed. In addition, data across regions will be pooled to estimate the overall probability of vaccine take as a function of preexisting Ad5 neutralizing antibody titer. Results from these secondary analyses of trial data will also help in determining the design and projected sample size of future efficacy trials.

HVTN 204 will be initiated after a review of all available safety data from the several ongoing clinical trials evaluating the same or similar VRC candidate HIV vaccines. Specifically, this includes protocols evaluating 3 doses of 4 mg of DNA vaccines alone (VRC 001, VRC 004, VRC 007, and HVTN 052), 1 dose of 1 x 10^{10} PU of the adenoviral vector alone (VRC 006) and the combination of DNA prime and adenoviral vector boost (HVTN 057 and VRC 009).

5.2 Plans for future product development and testing

This Phase II study is on the critical path to efficacy evaluation of this prime-boost approach. If found to be sufficiently safe, tolerable and immunogenic, the efficacy evaluation will compare prime-boost to placebo. This design could be altered if HVTN 204 does not demonstrate that the prime-boost is sufficiently safe or immunogenic.

FOR REVIEW ONLY

STUDY DESIGN

FOR REVIEW ONLY

6 Study objectives

6.1 Primary objectives

Safety

- Evaluate the safety and tolerability of 3 VRC HIV-1 DNA-6-plasmid vaccine doses at 4.0 mg/dose followed by VRC HIV-1 rAd5 vaccine boost at 10^{10} PU in HIV-1 uninfected adults.

Immunogenicity

- Evaluate the immunogenicity of 3 VRC HIV-1 DNA-6-plasmid vaccine doses at 4.0 mg/dose followed by VRC HIV-1 rAd5 vaccine boost at 10^{10} PU in HIV-1 uninfected adults.

6.2 Secondary objectives

Immunogenicity

- To evaluate the frequency of humoral immune responses to 3 doses of the HIV-1 DNA vaccine followed by the adenoviral vector vaccine. Humoral immunogenicity at 4 weeks post final vaccination will be assessed by neutralizing antibody and binding assays.
- To assess the relationship between prior Ad5 neutralizing antibody and immunogenicity as assessed by IFN-γ ELISpot, ICS, and neutralizing antibody assay.
- To evaluate the frequency and durability of cellular immunogenicity.
- To characterize the magnitude and breadth (defined as response to individual peptide pools) of the vaccine-induced HIV-specific T-cell responses as measured by the IFN-γ ELISpot and ICS assays.
- To evaluate the cross-clade cellular responses to HIV-1 Gag, Pol, and Nef peptides from clades A and C as assessed by the IFN-γ ELISpot and ICS.

Social impacts

- To evaluate the social impacts of trial participation.

FOR REVIEW ONLY

7 Study type, study population, and eligibility criteria

The study is a Phase II multicenter, randomized, placebo-controlled, double-blind trial to evaluate the safety and immunogenicity of VRC-HIVDNA016-00-VP followed by VRC-HIVADV014-00-VP. Participants will be healthy HIV-1-uninfected (seronegative) adults, 18-50 years of age, who comprehend the purpose of the study and have provided written informed consent. Participants will be recruited and screened; those determined to be eligible will be enrolled in the study and followed for a period of 12 months.

Participants will receive injections at 4 time points, administered at 0, 1, 2, and 6 months (see Overview). All injections will be administered by intramuscular injection in the outpatient setting.

See Table 7-1 for inclusion criteria and Table 7-2 for exclusion criteria. Final eligibility determination will depend on results of laboratory tests, medical history, physical examinations, and answers to the self-administered and/or interview questions.

See Section 12 for screening procedures.

FOR REVIEW ONLY

Table 7-1 Study inclusion criteria

Investigators should always use good clinical judgment in considering a volunteer's overall fitness for trial participation. Some volunteers might not be appropriate for enrollment even if they meet all inclusion /exclusion criteria because medical, psychiatric, or social conditions might make evaluation of safety and / or immunogenicity difficult.

General

Age: 18 to 50 years

Access to a participating HVTU and willingness to be followed for the planned duration of the study

Assessment of understanding: Complete a questionnaire prior to first vaccination; verbalize understanding of all questions answered incorrectly

Willingness to receive HIV test results

Informed consent: Be able and willing to provide informed consent

Health: Be in good general health as shown by medical history, physical exam, and screening laboratory tests performed within 56 days of enrollment.

Laboratory

Hemoglobin: ≥ sex-specific institutional lower limit of normal and at least 11.0 g/dL for women, 12.5 g/dL for men

WBC count = 3,300 to 12,000 cells/mm^3

Total lymphocyte count ≥ 800 cells/mm^3

Remaining differential either within institutional normal range or accompanied by site physician approval

Platelets = 125,000 to 550,000 cells/mm^3

ALT does not exceed 1.25 the upper limit of institutional normal ranges

Creatinine does not exceed institutional upper limit of normal

Negative HIV blood test: US participants must have a negative FDA-approved ELISA test. Non-US sites will use locally available and locally approved assays

Normal urine:

- Negative urine glucose and
- Negative or trace urine protein

Additional inclusion criteria for female participants

Negative serum or urine β-HCG pregnancy test performed on the day of initial vaccination prior to vaccination

Reproductive status: a female participant must:

- agree to consistently use contraception for at least 21 days prior to enrollment until 9 months post enrollment, for sexual activity that could lead to pregnancy. Contraception is defined as using any of the following methods:
    - condoms (male or female) with or without a spermicide
    - diaphragm or cervical cap with spermicide
    - intrauterine device (IUD)
    - hormonal contraception
    - successful vasectomy in the male partner (considered successful if a woman reports that a male partner has [1] microscopic documentation of azoospermia, or [2] a vasectomy more than 2 years ago with no resultant pregnancy despite sexual activity post-vasectomy)
- or not be of reproductive potential, such as having reached menopause (no menses for one year) or having undergone hysterectomy, bilateral oophorectomy, or tubal ligation.
- and agree not to seek pregnancy through alternative methods such as artificial insemination or in vitro fertilization until last protocol visit.

FOR REVIEW ONLY

Table 7-2 Study exclusion criteria

Participant has received any of the following substances:

HIV vaccine in a prior HIV vaccine trial. For potential participants who have received control/placebo in an HIV vaccine trial, documentation of the identity of the study control/placebo must be provided to the HVTN 204 Protocol Safety Review Team to determine eligibility on a case-by-case basis.

Immunosuppressive medications within 168 days before first vaccination, e.g., oral/parenteral corticosteroids, and/or cytotoxic medications. *Not excluded: (1)corticosteroid nasal spray for allergic rhinitis; (2) topical corticosteroids for mild, uncomplicated dermatitis; (3) a short course of rapidly tapered steroids (fewer than 10 days of treatment) more than 90 days before first vaccination*

Blood products within 120 days before first vaccination

Immunoglobulin within 60 days before first vaccination

Live attenuated vaccines within 30 days before first vaccination

Investigational research agents within 30 days before first vaccination

Medically indicated subunit or killed vaccines, e.g., influenza within 14 days, pneumococcal within 14 days, or allergy treatment with antigen injections within 30 days prior to initial study vaccine administration

Current anti-TB prophylaxis or therapy

Participant has a clinically significant medical condition, physical examination findings, clinically significant abnormal laboratory results, or past medical history with clinically significant implications for current health. A clinically significant condition or process includes but is not limited to:

- a process that would affect the immune response
- a process that would require medication that affects the immune response
- any contraindication to repeated injections or blood draws
- a condition that requires active medical intervention or monitoring to avert grave danger to the participant's health or well-being during the study period
- a condition or process in which signs or symptoms could be confused with reactions to vaccine
- any condition specifically listed among the exclusion criteria below

Any medical, psychiatric, or social condition, or occupational or other responsibility that, in the judgment of the investigator, would interfere with, or serve as a contraindication to, protocol adherence, assessment of safety or reactogenicity, or a participant's ability to give informed consent

Serious adverse reactions to vaccines including anaphylaxis and related symptoms such as hives, respiratory difficulty, angioedema, and/or abdominal pain. *Not excluded: A participant who had a nonanaphylactic adverse reaction to pertussis vaccine as a child.*

Autoimmune disease

Immunodeficiency

Active syphilis infection. *Not excluded: Syphilis fully treated over six months ago.*

Asthma that is greater than mild in severity. Specifically excluded are participants with any of the following within the past year:

- daily symptoms
- daily use of short acting beta2 agonists
- frequent exacerbations of symptoms that interfere with daily activity
- use of moderate to high dose inhaled corticosteroids (e.g. more than the equivalent of 250 mcg fluticasone; 400 mcg budesonide; 500 mcg beclomethasone; or 1000 mcg triamcinolone/flunisolide, as a daily dose) or theophylline
- emergent care, urgent care, hospitalization or intubation for asthma

Diabetes mellitus type I or type II, including cases controlled with diet alone. *Not excluded: Isolated gestational diabetes.*

Thyroid disease or thyroidectomy requiring medication during the last 12 months.

Angioedema within the last 3 years if episodes are considered serious or have required medication within the last 2 years

Hypertension that is not well controlled by medication, or blood pressure ≥ 150/100 (either or both values) at enrollment

Bleeding disorder diagnosed by a doctor, e.g., factor deficiency, coagulopathy, or platelet disorder requiring special precautions

Malignancy *Not excluded: A participant with a surgical excision and subsequent observation period that in the investigator's estimation has a reasonable assurance of sustained cure and/or is unlikely to recur during the period of the study*

Seizure disorder *Not excluded: A participant who had febrile seizures under the age of 3*

Asplenia: any condition resulting in the absence of a functional spleen

Psychiatric condition that precludes compliance with the protocol. Specifically excluded are persons with any of the following:

- psychoses within the past 3 years
- ongoing risk for suicide
- history of suicide attempt or gesture within the past 3 years

Additional exclusion criteria for female participants: Pregnancy, or breast feeding, or plan to become pregnant during the period of study participation

FOR REVIEW ONLY

8 Safety and immunogenicity evaluations

8.1 Considerations for trial start

Enrollment in this study will begin only after the combination of 4 mg of VRC-HIVDNA-009-00-VP DNA prime and 10^{10} PU of VRC-HIVADV014-00-VP adenoviral vector boost has been shown to be well tolerated in the HVTN 057 trial. The HVTN 204 Protocol Safety Review Team will review all of the data available 2 weeks after enrollment of at least 60 participants in HVTN 057. If necessary, an ad hoc unblinded review of the data will be performed by the HVTN Safety Monitoring Board (SMB).

8.2 Distinguishing intercurrent HIV infection from vaccine-induced positive serology

The study products may elicit an antibody response to HIV proteins. Therefore, vaccine-induced positive serology may occur in this study. Several precautionary measures will be taken to clarify this distinction:

- Participants will be counseled frequently during the trial on avoidance of HIV infection.
- Participants will be counseled on the risks of seeking HIV testing outside of the network during study participation, and discouraged from doing so.
- Participants will have clinical evaluations at visits specified in Appendix B. Signs or symptoms of an acute HIV infection syndrome, an intercurrent illness consistent with HIV-1 infection, or probable HIV exposure would prompt a diagnostic work-up per the standard HVTN algorithm to determine HIV infection.
  - Diagnostic HIV-1 ELISAs will be performed from blood draws at multiple time points throughout the trial (see Appendix A).
  - For US sites the HIV diagnostic Laboratory is Richmond. For Non-US sites, local labs may perform HIV diagnostic algorithms (following HVTN SOP) with pre-approval from the HVTN Laboratory Operations Division.
  - If intercurrent HIV-1 infection is suspected or positive test results are observed post-vaccination, the Laboratory Program or approved diagnostic laboratory will proceed with the HVTN algorithm to distinguish vaccine-induced antibody responses from actual HIV infection.
- Continued follow-up will identify subsequent HIV infections or address concerns in participants whose HIV-1 ELISA is positive or indeterminate at the end of the study. All participants who have positive or indeterminate HIV-1 serology at the last study visit (as measured by the Abbott HIV 1, 2 kit or other standard anti-HIV antibody screening test used by blood banks) will be offered follow-up HIV-1 diagnostic testing (HIV-1 ELISA, Western blot, PCR) periodically and free of charge as medically/socially indicated (approximately every 6 months). This follow-up will be available until the ELISA/Western blot pattern no longer yields positive or indeterminate results or until HIV infection is confirmed.
- Potential participants identified as being HIV infected during screening and participants who become HIV infected during the study will be referred for medical

FOR REVIEW ONLY

treatment and management of the HIV infection. These individuals will also be referred to appropriate ongoing clinical trials or observational studies.

8.3 Immunogenicity evaluation

The ability of the vaccine to induce humoral responses and/or epitope-specific CD8+ and CD4+ T cell responses will be evaluated by the methods described below. For all assays, cryopreserved specimens from additional time points of immunological interest, as indicated in Appendix A, may be tested if positive responses are detected at the primary immunogenicity endpoints. All assays listed as primary immunogenicity endpoints must have undergone documented validation. Validation packages must be available for HVTN Laboratory Program and VRC review.

8.3.1 Humoral immunogenicity studies

8.3.1.1 Binding antibodies by ELISA (HVTN)

Binding antibodies to commercially available Env will be assessed at the HVTN-CL by ELISA using single serum dilutions (1/50 or 1/100) on samples from all study participants taken at the baseline and at the four week post last vaccination visit. Any of the time points that yield positive results, defined as an OD of ≥ 0.2, in the initial ELISA may be subject to endpoint titration ELISA employing 6 (2-7-fold) serial dilutions of serum beginning at a 1/50 or 1/100.

8.3.1.2 Neutralizing antibody assay (HVTN)

HIV-1 specific neutralizing Ab assays will be performed on serum samples from all study participants taken at baseline and at the four week post last vaccination visit (see Appendix A). The assays will test neutralization of HIV-1 MN and the HIV-1 strains represented in the vaccine constructs (HXB2, Bal, 92rw020, and 97ZA012). The serum samples with the highest antibody response (as judged by either endpoint ELISA titer >100 or neutralization) may be further assayed against a panel of heterologous primary isolates and analyzed separately.

As an exploratory assay, the HVTN may examine neutralizing antibody responses against a panel of heterologous primary isolates using cross-sectional serum samples. If neutralizing antibodies are detected, a subset of samples with the best neutralizing activity will be screened at a single serum dilution for neutralization activity against a panel of heterologous strains.

8.3.1.3 Binding and neutralizing antibody assays (VRC)

Binding antibody to clades A, B, and C gp140 proteins will be performed by the VRC at the time points specified in Appendix A. Depending on the results of these assays, the VRC may perform Env-pseudovirus luciferase neutralization assays on panels of clades A, B, C and D viruses.

8.3.1.4 Neutralizing antibody against viral vector (Ad5)

The VRC laboratory will evaluate preexisting adenovirus neutralizing antibodies at screening and neutralizing antibodies produced against the adenoviral vector at four weeks post final vaccination.

FOR REVIEW ONLY

8.3.2 Cellular immunogenicity studies

8.3.2.1 IFN-γ ELISpot (HVTN and VRC)

Ex vivo T cell responses will be assessed by IFN-γ ELISpot using cryopreserved peripheral blood mononuclear cells (PBMC) stimulated overnight with synthetic peptide pools that span the proteins encoded by the vaccine constructs. ELISpot assays will be performed at the baseline and at the four week post last vaccination visit by the VRC. ELISpot assays will be performed at the baseline and at the six week post last vaccination visit by the HVTN (see Appendix A). Responses will be reported as number of spot forming cells (SFC) per 10^6 cells/well recognizing any specific peptide pool.

8.3.2.2 Intracellular cytokine staining (HVTN and VRC)

HVTN

Flow cytometry will be used to examine HIV-specific CD4+ and CD8+ T cell responses using intracellular cytokine staining (ICS) following stimulation with synthetic HIV peptides that span the proteins encoded by the vaccine construct. ICS assays will be performed at the baseline and at the six week post last vaccination visit (see Appendix A). Responses will be reported as percentages of CD4+ or CD8+ T cells expressing IFN-γ and/or IL-2 in response to any specific peptide pool.

VRC

A flow cytometric assay for the detection of ICS will be performed at the VRC Lab after stimulation with synthetic peptides that span the specific multiclade HIV-1 proteins encoded by the vaccine construct at baseline and at the four week post last vaccination visit (see Appendix A). Responses will be reported as percentages of T cells (CD4+, CD8+, both) expressing IFN-γ and/or IL-2 in response to any specific peptide pool.

8.4 HLA typing

Molecular HLA typing may be performed on enrolled participants using cryopreserved PBMC collected at baseline, initially in participants who demonstrate vaccine-induced T-cell responses at post-vaccination time points. Other participants (including placebo recipients) may be HLA-typed to support future studies of immunological interest at the discretion of the protocol chair and the Laboratory Program. These assays may include, but are not limited to, fine epitope mapping by flow cytometry or ELISpot, or flow cytometric tetramer analysis.

8.5 Ancillary studies

Cryopreserved samples may be used to perform additional ELISpot and ICS assays to support the standardization and validation of these assays, and in other immunological assays of interest to guide future research. These assays may include, but are not limited to, fine epitope mapping by flow cytometry or ELISpot, flow cytometric tetramer analysis, or genetic marker analysis.

FOR REVIEW ONLY

FOR REVIEW ONLY

9 Statistical considerations

9.1 Overview

The data analysis will characterize safety and immunogenicity data of the study products, a multiclade HIV DNA plasmid vaccine followed by a recombinant adenoviral vector vaccine boost.

9.2 Objectives

The primary objectives of this trial concern safety and immunogenicity of the study vaccines. The secondary objectives concern secondary immunogenicity endpoints and social impacts. See Section 6 for details.

9.3 Endpoints

9.3.1 Safety

Assessment of product safety will include clinical observation and monitoring of hematological and chemical parameters. Safety will be evaluated by monitoring participants for local and systemic adverse reactions after each injection and for 12 months after the first injection. Section 14 describes the safety monitoring plan and reports.

The following parameters will be assessed:

- Local reactogenicity signs and symptoms
- Systemic reactogenicity signs and symptoms
- Laboratory measures of safety
- Adverse and serious adverse experiences

9.3.2 Immunogenicity

The primary immunogenicity endpoints are:

- unfractionated IFN-γ ELISpot responses to HIV-1 peptide pools as performed by the VRC laboratory at Day 196. The data generated will contribute to the data package that the VRC/DAIDS will use to determine whether the products qualify for further evaluation in an efficacy trial.
- unfractionated IFN-γ ELISpot responses to HIV-1 peptide pools as performed by the FHCRC laboratory at Day 210. The data generated will be used by the HVTN for independent evaluation of the products.
- CD4+ and CD8+ T cell responses to HIV-1 peptide pools as measured by flow cytometry-based intracellular cytokine staining (ICS) assay as performed by the VRC laboratory at Day 196. The data generated will contribute to the data package to be used by the VRC/DAIDS to determine whether the products qualify for further evaluation in an efficacy trial.
- CD4+ and CD8+ T cell responses to HIV-1 peptide pools as measured by flow cytometry-based ICS assay as performed by the FHCRC laboratory at Day 210. The data generated will be used by the HVTN for independent evaluation of the products.

Secondary immunogenicity endpoints are:

- humoral immune response to HIV-1 as measured by neutralizing antibody and binding assays
- unfractionated IFN-γ ELISpot responses to HIV-1 as performed by the FHCRC or the VRC laboratories at Days 70, 210, and 364
- CD4+ and CD8+ T cell responses to HIV-1 as measured by flow cytometry-based ICS assay as performed by the FHCRC or the VRC laboratories at Days 70, 210, and 364
- Vaccine-induced HIV-specific T cell responses to individual peptide pools as measured by IFN-γ ELISpot and ICS as performed by the FHCRC or the VRC laboratories
- cross-clade cellular immune responses to HIV-1 Gag-Pol-Nef peptides from clades A and C as assessed by IFN-γ ELISpot and ICS assays as performed by the FHCRC or the VRC laboratories.

Other endpoints used in the immunogenicity assessment

- prior Ad5 antibody responses as measured by neutralizing antibody assay as performed by the VRC laboratory at baseline

9.3.3 Social impacts

Social impact variables include any negative experiences or problems the participant experienced due to his/her participation in this study. The following social impacts will be followed during the course of the study: social, travel, work, school, health care, life insurance, health insurance, housing, military and any additional impacts identified by a participant.

9.4 Accrual and sample size

Recruitment will target 480 healthy, HIV-uninfected adults. Within each of two regions (U.S./Caribbean and Southern Africa), 120 volunteers will be enrolled into the vaccine group and 120 volunteers will be enrolled into the placebo group. The total number of volunteers is 240 in the vaccine group and 240 in the placebo group. A large number of placebo recipients are included to permit robust characterization of safety and tolerability, and so that specimens from a subset of placebo recipients can be used to validate laboratory assays. Participants are enrolled without use of the data on preexisting Ad5 titers; that is these titers are not used as enrollment criteria. The study aims to enroll approximately equal numbers of men and women, such that at least 40% of the study population is male and at least 40% of the study population is female. If an interim assessment of the proportion of males and females enrolled reveals a large imbalance of one gender, future enrollments will seek to redress the imbalance.

Since enrollment is concurrent with receiving the first study vaccination, all participants will provide some safety data hence sample size calculations for safety in Section 9.4.1 are based on the target sample sizes. However this is not true of the immunogenicity data, therefore the sample size calculations in Section 9.4.2 account for 10% of enrolled participants having missing data for the primary endpoints, based on previous HVTN and AVEG studies.

FOR REVIEW ONLY

9.4.1 Sample size calculations for safety

The goal of the safety evaluation for this study is to identify safety concerns associated with injection. Sample size calculations are expressed in terms of the ability to detect serious adverse experiences.

The sensitivity of the study to identify serious adverse experiences is best expressed by the maximum true rate of events that would be unlikely to be observed and the minimum true rate of events that would very likely be observed. Specifically, for the vaccine group where n=240, there is a 90% chance of observing at least one serious adverse experience if the true rate of such an event is at least 1.0%; there is a 90% chance that we would not observe at least one serious adverse experience if the true rate was no more than 0.05% (that is, 5/10,000 or less in the population). Probabilities of observing zero or two or more serious adverse experiences among a group of size 240 are presented in Table 9-1 for a range of possible true event rates. These calculations provide a more complete picture of the sensitivity of this study design to identify potential safety problems with the vaccine.

If none of the 240 participants receiving the vaccine experience life-threatening (grade 4) reactions to the given vaccine, the 95% exact two-sided upper confidence bound for the rate of such reactions in the population is 2%. If 10 of the 240 vaccinees experience such reactions, the upper bound for the rate is 9%. Suppose we assume based on data from previous AVEG studies that the rate of serious adverse events (SAEs) in controls is approximately 3.5%. Then for a two-sided exact test, type I error of 0.05, and power of 90%, the difference between a 3.5% SAE rate in the controls (n=240) and a 11.4% SAE rate in the vaccinees (n=240) is detectable.

Table 9-1 Probability of response for different safety scenarios for vaccinees (n=240)

Event rate	Pr(0/240 events)	Pr(1+/240 events)	Pr(2+/240 events)
0.05%	0.89	0.11	0.007
0.10%	0.79	0.21	0.025
0.50%	0.30	0.70	0.34
1%	0.09	0.91	0.69

9.4.2 Sample size calculations for immunogenicity

A primary objective is to characterize the immunogenicity of the two vaccine regimens. After accounting for a 10% dropout rate, the sample size of the vaccine group (n=216) allows a reasonable bound on the true response rates for the immunogenicity endpoints. For example, if the observed CD8+ T cell response rate is 70%, the exact 95% confidence interval is (63%, 76%). Confidence intervals of other response rates can be found in Table 9-2.

As sample sizes are large in this trial, there is sufficient power for a comparison of immune response rates between the control group (n=216) and the vaccine group (n=216). For example, a difference between a 3.5% control group (n=216) CD8+ T cell response rate and a 10.7% vaccine regimen response rate (n=216) is detectable with 80% power for a two-sided exact test with a Type I error rate of 0.05.

FOR REVIEW ONLY

Table 9-2 Exact two-sided confidence intervals (CI) for the CD8+ T-cell response rate based on observing a particular rate of responses in vaccinees (n=216). CI's for larger loss-to-follow-up (LTFU) rates are also shown, n=204 with 15% LTFU, n=192 with 20% LTFU.

Observed response rate	95% CI (n=216)	95% CI (n=204)	95% CI (n=192)
50%	(43%,57%)	(43%,57%)	(43%,57%)
60%	(53%,67%)	(53%,67%)	(53%,67%)
70%	(63%,76%)	(63%,76%)	(63%,76%)
80%	(74%,85%)	(74%,85%)	(74%,85%)
90%	(85%,94%)	(85%,94%)	(85%,94%)

A secondary immunogenicity objective is to characterize the immune response rates of the vaccine regimen as a function of preexisting Ad5 antibody titer. Particular attention will be paid to the right tail of the function, where Ad5 titers exceed 1:500, as we wish to determine whether there exists a threshold Ad5 titer above which response rates are very low. The power calculations for this objective consider a series of 3 Fisher's Exact tests with Bonferroni adjustment for multiplicity to identify the presence of a threshold. Each tests for a difference in the number of positive responders above and below one of 3 fixed quantiles: 0.25, 0.50, 0.75. Response probabilities are simulated from a logistic model with pmax, pmin, and inflection point variables listed in Table 9-3. The slope of the model was set at –15 to give a sharp decline in response to mimic a threshold. The calculations in Table 9-3 assume n=100 vaccinees have pre-existing Ad5 titers > 1:500, after accounting for a 10% loss to follow-up rate. Note, there is over 80% power to detect a difference in response rates with at least one of the Fisher's Exact tests when pmax=40%, pmin=5% and the inflection point occurs at the 0.30 quantile. Power decreases when the inflection quantile equals 0.8 although arguably we are less concerned with such situations as they imply response rates are close to pmax for 80% of observed Ad5 titers > 1:500.

Table 9-3: Power to detect a difference in at least one of the 3 Fisher's Exact tests.

pmax	pmin	Quantile of inflection	Power (n=100)
50%	10%	0.3	0.79
50%	10%	0.6	0.88
50%	10%	0.8	0.56
40%	10%	0.3	0.56
40%	10%	0.6	0.64
40%	10%	0.8	0.35
40%	5%	0.3	0.81
40%	5%	0.6	0.84
40%	5%	0.8	0.50

9.4.3 Sample size calculations across HVTN 204, RV172, and IAVI-V001

Taken together, the three studies, HVTN 204, RV172, and IAVI-V001, are well-powered to qualify the vaccine regimen for expanded studies of vaccine efficacy in the geographic regions of the trials (see Section 5.1). For each of the geographic regions, these studies will develop data that will enable: 1) bounding (by a 95% confidence limit) crude rates of serious adverse events related to vaccination to less than 3-6% if 0-2 such events are observed among the 120 vaccinees, and 2) detecting net rates of vaccine take to be greater than 30% if the true

FOR REVIEW ONLY

rates of vaccine take are at least 47%. For example, HVTN 204 is able to detect differences in adverse event rates of 3.5% in the placebo arm versus 11% in the vaccine arm (or 3.5% versus 15% for immune response rates after accounting for 10% loss to follow-up). The immune response rate in the vaccine arm can be estimated with precision plus or minus 7%; for example, if the observed immune response rate is 50% in a given geographic region, then the 95% confidence interval about this rate is 40% to 60%. Power and precision are greater when the data from RV172 and IAVI-V001 are included in the analysis.

9.5 Statistical analysis

All data from enrolled participants will be analyzed according to the initial randomization assignment regardless of how many vaccinations they received. Since enrollment is concurrent with receiving the first study vaccination, all participants will have received 1 vaccination and therefore will provide some safety data. The analysis is intent-to-treat; however, individuals who are randomized but not enrolled do not contribute data and hence are excluded. Because of blinding and the brief length of time between randomization and enrollment—typically no more than 4 working days, according to the *HVTN Manual of Operations* (HVTN MOP) (Study Operations >Enrollment >Randomization)—very few such individuals are expected.

All descriptive and inferential statistical analyses will be performed using SAS and S-Plus statistical software. When the use of descriptive statistics to assess group characteristics is required, the following statistics will be reported: for categorical variables, the number and percent in each category; for continuous variables, the mean, median, standard deviation, quartiles, and range (minimum, maximum).

No formal multiple comparison adjustments will be employed for safety endpoints, primary immunogenicity endpoints that address separate scientific questions (e.g., humoral- and cellular-based endpoints), or secondary endpoints. Multiple measurements of a specific type of immune response will be treated as a collection of hypotheses that requires a multiplicity adjustment. For example, determination of cellular immune responses to several different HIV-1 peptide pools as measured by the IFN-γ ELISpot assay will entail a multiplicity adjustment to account for the multiple peptide pools considered.

9.5.1 Analysis variables

The analysis variables consist of baseline variables, safety variables, immunogenicity variables, and social impact variables for primary and secondary objective analysis.

9.5.2 Baseline comparability

Groups will be compared for baseline characteristics including demographics and laboratory measurements, using descriptive statistics.

9.5.3 Safety analysis

Reactogenicity

The number and percentage of participants experiencing each type of reactogenicity sign or symptom will be tabulated by severity and vaccine regimen. For a given sign or symptom, each participant's reactogenicity will be counted once under the maximum severity for all injection visits.

FOR REVIEW ONLY

Adverse experiences

Adverse experiences are coded into MedDRA preferred terms. The number and percentage of participants experiencing each specific adverse experience will be tabulated by severity and by relationship to treatment. For the calculations in these tables, each participant's adverse experience will be counted once under the maximum severity or the strongest recorded causal relationship to treatment.

A complete listing of serious adverse experiences for each participant will provide details including severity, relationship to treatment, onset, duration and outcome.

Local laboratory values

Boxplots of local laboratory values by treatment will be generated for baseline values and for values measured during the course of the study. Each boxplot will show the first quartile, the median, and the third quartile. Outliers or values outside the boxplot will also be plotted. Horizontal lines representing boundaries for abnormal values will be plotted, if applicable.

9.5.4 Immunogenicity analysis

The statistical analysis for immunogenicity will employ the intent-to-treat principle, i.e., all data from enrolled participants will be used according to the initial randomization assignment regardless of how many injections they received. The only exception will be to exclude data from HIV-infected participants at or post infection. If the HIV positivity status of an infected participant is unknown at the time that the first sample for immunogenicity assessments is drawn, then all data from that participant will be excluded from the analysis.

If assay data are qualitative (i.e., positive or negative), then analyses will be performed by tabulating the frequency of positive response for each assay by vaccination group at each time point that an assessment is performed. All response rates mentioned here are net responses; there will be sufficient data to adjust for false positive responses from the control group in this trial. Binomial response rates will be presented with their corresponding exact two-sided 95% confidence interval estimates. A significant difference in crude response rates between the active and control group will be declared if the two-sided 95% confidence interval for the difference excludes zero. In addition to reporting the point prevalence response rates, cumulative and cumulative repeat probabilities of cellular immune responses will be estimated with corresponding confidence intervals using maximum likelihood based methods [89]. Missing responses will be assumed to be missing at random, i.e. conditional on the observed data, the missingness is independent of the unobserved responses.

For continuous assay variables, overall differences between groups at a specific time point will be tested by a two-sample t-test if the data appear to be normally distributed. If not, the nonparametric Wilcoxon rank sum test will be used. If a portion of the measurements is censored below the assay quantification limit, the Gehan-Wilcoxon test will be employed. More sophisticated analyses employing repeated measures methodology (for example, repeated measures ANOVA or generalized estimating equations) may be used to incorporate immune responses over several time points. All statistical tests will be two-sided and will be considered statistically significant if $p \leq 0.05$. Graphical descriptions of the longitudinal immune responses will also be given.

Regression methods will be used to model immunogenicity as a function of vaccine regimen and preexisting Ad5 neutralizing antibody titer. Moulton and Halsey's mixed gamma model will be considered.

Some immunologic assays have underlying continuous or count-type readout that is often dichotomized into responder/nonresponder categories. For these assays, graphical and tabular

FOR REVIEW ONLY

summaries of the underlying distributions will be made. These summaries may be performed on transformed data (e.g., log transformation) to better satisfy assumptions of symmetry and homoscedasticity. If group comparisons in these underlying distributions reveal that differences are best summarized as a shift in the location of the distribution, then results will be presented in the form of group means (or medians) with associated confidence intervals and statistical tests for differences between groups as described above. If group comparisons in these underlying distributions reveal that differences are best summarized by a mixture model (i.e., responder and nonresponder subgroups are clearly identifiable) then results will be presented in the form of response rates with associated confidence intervals and statistical tests as described above.

Missing data

If the probability of missing immunogenicity measurements depends on either covariates or on the immunogenicity outcomes of participants, then the methods described above may give biased inferences and point estimates. If a substantial amount of immunogenicity data is missing (at least 1 value missing from more than 20% of participants), then secondary analyses of the immunogenicity endpoints will be conducted using methods that relax the missing completely at random assumption to a missing at random assumption. For a univariate binary and quantitative outcome, respectively, a generalized linear model with a binomial or normal error distribution will be used for estimation and testing. For assessing repeated immunogenicity measurements, linear mixed effects models will be used. The models will be fit using maximum likelihood methods, and will include as covariates all available baseline predictors of the missing outcomes. The longitudinal models will also include all observed immunogenicity data.

9.5.5 Social impact analysis

Social impacts are assessed at the Day 70, 168, and 364 visits.

Social impacts will be tabulated by type of event and impact on quality of life. The number and percentage of participants experiencing each type of social impact will also be tabulated by impact on quality of life. For this calculation multiple events of the same type for a participant will be counted once under the maximum impact for all post-vaccination visits.

In addition, for each type of social impact a longitudinal listing will be generated for all participants who experienced a major disturbance starting with the first major disturbance followed by all subsequent impacts. Included in this listing will be descriptions of the impact, duration, impact on quality of life, actions taken by the participant and staff, and whether or not there was a resolution.

9.5.6 Interim analyses

Unblinded interim analyses of safety data are prepared at regular intervals for the Safety Monitoring Board (SMB).

An interim analysis of unblinded prior Ad5 antibody responses will be performed after at least one-half of the participants have been enrolled to ensure an approximate balance of low and high titer participants in the trial (see Section 9.6).

In order to efficiently promote the development of the vaccine candidate, immunogenicity data taken one month after the Ad5 boost time point will be analyzed along with the cumulative safety data and adenovirus serologies for all volunteers, by study group (vaccine vs. placebo). Analysis will be performed according to an official Statistical Analysis Plan (SAP) to be developed by NIAID in collaboration with HVTN and its principal investigators.

FOR REVIEW ONLY

Should the Sponsor and collaborators determine that analysis of the immunogenicity data needs to be performed prior to availability of data for the entire targeted enrollment, that analysis will be performed according to a modified SAP developed by NIAID and the HVTN.

In addition, an official SAP for the analysis of the combined data from the three studies (HVTN 204, RV 172 and IAVI V001) will be developed by NIAID in collaboration with the three networks (HVTN, USMHRP and IAVI).

9.6 Randomization of treatment assignments

The randomization sequence will be obtained by computer-generated random numbers and provided to each HVTU by the SDMC using the procedures described in the HVTN MOP. At each institution, the pharmacist with primary responsibility for drug dispensing is charged with maintaining security of the randomization list. The randomization will be stratified by geographic region and done in blocks to ensure balance across groups. Following an interim assessment of the distribution of pre-existing Ad5 antibody titers among participants enrolled in the trial, future enrollment may be redirected to certain sites in order to ensure that an approximate balance of low and high titer participants are enrolled. An approximate balance is defined as between 40% and 60% of participants with prior Ad5 titers >1:500. The interim assessment will also be used to verify that there is an adequate representation of men and women in the trial (e.g., the proportion of either cohort is at least 0.40).

FOR REVIEW ONLY

STUDY OPERATIONS

FOR REVIEW ONLY

10 Protocol conduct

The protocol will be conducted according to standard HVTN policies and procedures specified in the HVTN MOP (Study Operations), including procedures for the following:

- Protocol registration, activation and implementation
- Informed consent, screening, enrollment
- Clinical and safety assessments
- Safety monitoring and reporting
- Data collection and documentation
- Study follow-up and close-out
- Unblinding of staff and participants
- Quality control
- Protocol monitoring and compliance
- Advocacy and assistance through local and governmental activities to participants regarding social harms associated with the vaccine trial
- Risk reduction counseling
- Outside testing and belief questionnaire

Any policies or procedures that vary from HVTN standards or require additional instructions will be described in the *HVTN 204 Study Specific Procedures* (e.g., instructions for randomization specific to this study).

FOR REVIEW ONLY

11 Informed consent

Informed consent is the essential process of ensuring that participants fully understand what will and may happen to them while participating in a research study. The HVTN informed consent form documents that a participant (1) understands the potential risks, benefits, and alternatives to participation, and (2) is willing to participate in an HVTN study. Informed consent is not confined to the signing of the consent form; it also includes all written or verbal study information HVTU staff discuss with the participant, before and during the trial. HVTU staff will obtain informed consent of participants according to the HVTN policies and procedures specified in the HVTN MOP (Study Operations>Informed Consent).

An HVTU may employ recruitment efforts prior to the participant consenting. Some HVTUs use a telephone script to pre-screen people before they come to the clinic for a full screening visit. Participants must sign a screening or protocol-specific consent before any procedures to determine eligibility are performed. HVTUs must submit recruitment and pre-screening materials to IRBs/IECs for human subjects review.

11.1 Screening consent form

Some HVTUs have approval from their local Institutional Review Board (IRB) and/or Independent Ethics Committee (IEC) to use a general screening consent form that allows screening for an unspecified HIV vaccine trial. In this way, HVTU staff can continually screen potential participants, and when needed, proceed quickly to obtain protocol-specific enrollment consent. Sites conducting IRB/IEC–approved general screening or pre-screening may use the results from this screening for determining eligibility in this protocol, provided the tests are conducted within the time period specified in the eligibility criteria.

11.2 Protocol-specific consent form

The protocol-specific consent form describes the study products to be used and all aspects of protocol participation, including screening and enrollment procedures.

Each HVTU is responsible for developing a protocol-specific consent form for local use, based on the sample protocol-specific consent form. The consent form must be developed in accordance with local IRB/IEC requirements and the principles of informed consent as described in Title 45, Code of Federal Regulations (CFR) Part 46 and Title 21 CFR, Part 50, and in the International Conference on Harmonisation (ICH) Guideline 4.8.10. It must be approved by all responsible ethical review bodies before any participants are consented for the study.

The DAIDS Regulatory Compliance Center (RCC) Protocol Registration Office will review all site-specific informed consent forms and approve them for use according to DAIDS policies. The study cannot be initiated at a site until the site is fully registered with the DAIDS RCC Protocol Registration Office and has received written notification of protocol activation.

11.3 Assessment of understanding

Study staff should ensure that participants fully understand the study before enrolling them. This involves reviewing the informed consent form with the participant, allowing time to reflect on the procedures and issues presented, and answering all questions completely.

FOR REVIEW ONLY

An Assessment of Understanding is used to document the participant's understanding of key concepts in an HIV vaccine trial.

The Assessment of Understanding should not be administered until the participant has signed a consent form. This can be a screening consent form, if the HVTU uses one. If not, the participant must sign the protocol-specific consent prior to administration of the Assessment of Understanding.

The participant must complete the Assessment of Understanding before first injection, and verbalize understanding of all questions answered incorrectly. This process, and the participant's understanding of the key concepts, should be documented in source documentation at the site.

FOR REVIEW ONLY

12 Procedures

Participants are considered to be enrolled only upon receipt of the first study vaccination at Day 0.

HVTU and HVTN Laboratory Program staff will conduct screening and post-enrollment study procedures according to HVTN procedures as specified in the HVTN MOP (Study Operations). Any procedures which vary from the HVTN standard will be defined in the *HVTN 204 Study Specific Procedures*.

Pre-enrollment and post-enrollment procedures are performed on all participants (unless otherwise noted) at the time points indicated in Appendix A and B, using the blood draw volumes specified.

12.1 Pre-enrollment procedures

An HVTU may employ recruitment efforts prior to the participant consenting. But before any protocol-specific questions are asked or procedures to determine protocol eligibility performed, the screening consent form or protocol-specific consent form (see Section 11) must be signed. Participants will be provided with a copy of all consent forms that they sign.

Screening assessments and other pre-enrollment procedures are listed in Table 12-1. Time points are specified in Appendix A and B.

The time interval between randomization and enrollment is typically 4 working days, as defined in the HVTN MOP (Study Operations). Subsequently, the HVTU registers the participant by scheduling the Day 0 visit (enrollment) via the web-based randomization system, and requests the randomization assignment.

Table 12-1 Pre-enrollment procedures

Screening assessments			
Clinical assessments	Local lab assessments		HIV infection assessments
Medical history	Pregnancy test (females)	Platelet count	HIV screening test
Complete physical exam	Urine dipstick/urinalysis	Creatinine	
Concomitant medications	CBC with differential	ALT	
Abbreviated physical exam	Syphilis test		

Other pre-enrollment procedures	
Screening informed consent (if applicable)	Behavioral risk assessment
Protocol informed consent	Risk reduction counseling
Assessment of understanding	Pregnancy prevention counseling
Specimen collection	HIV pre- and post-test counseling
Obtain demographics	Participant randomization
Confirm eligibility	

12.2 Post-enrollment procedures

Safety assessments, immunogenicity determinations, and other post-enrollment procedures are listed in Table 12-2.

FOR REVIEW ONLY

Table 12-2 Post-enrollment procedures

Safety assessments			
Clinical assessments	Local lab assessments		HIV infection assessments
Abbreviated physical exam	Pregnancy test (females)	Platelet count	HIV ELISA
Complete physical exam	Urine dipstick/urinalysis	Creatinine	HIV Western blot (if applicable)
Concomitant medications	CBC with differential	ALT	HIV RNA PCR
Intercurrent illness/AE			HIV DNA PCR
Reactogenicity			

Immunogenicity determinations		
Humoral assays (HVTN)	Cellular assay (HVTN)	Other assays (VRC)
HIV-1 neutralizing antibodies	IFN-γ ELISpot	Humoral studies
HIV-1 binding ELISA	ICS	IFN-γ ELISpot
		ICS
		Adenovirus neutralizing antibodies

Other post-enrollment procedures		
Vaccination administration	Risk reduction counseling	Outside testing/belief assessment
Specimen collection and shipping	Pregnancy prevention counseling	Cryopreservation/storage of specimens
Social impact assessment	HLA typing	Participant unblinding
HIV pre- and post-test counseling		

12.3 Total blood volumes

Required blood volumes are shown in Appendix A. Not shown is any additional blood volume that would be required if a safety lab needs to be repeated, or if a serum pregnancy test needs to be performed. The additional blood volume would likely be minimal. The total blood volume drawn for each participant will not exceed 500 mL in any 8-week period.

12.4 Laboratory procedures

A *Laboratory Procedures* manual will be available that provides further guidelines for operational issues concerning the clinical laboratories and phlebotomy. The procedures include general specimen collection guidelines, special considerations for blood collection, HIV testing guidelines, suggested tube types with catalog numbers, guidelines for processing whole blood, and labeling guidelines.

In specific situations the blood collection tubes will be redirected to another laboratory for special screening criteria or safety issues. In these cases special shipping instructions will be provided in Special Instructions posted on the HVTN website.

FOR REVIEW ONLY

13 Study product preparation and administration

HVTU pharmacists should consult the *Pharmacy Guidelines and Instructions for DAIDS Clinical Trials Networks* manual for standard pharmacy operations procedures. The protocol schema and vaccine regimen are shown in Section 13.1. See the Investigator's Brochure for further information about study products.

13.1 Schema and vaccine regimen

DNA: VRC-HIVDNA016-00-VP (Multiclade HIV-1 DNA Plasmid Vaccine, Multiclade HIV DNA Plasmid Vaccine, DNA)

DNA Placebo: Phosphate buffered saline (PBS)

rAD: VRC-HIVADV014-00-VP (clade B Gag and Pol; clades A, B, C Env, rAD)

rAD Placebo: VRC-DILUENT013-DIL-VP

		Injection schedule in months (days)			
Study arm	Treatment	0 (0)	1 (28)	2 (56)	6 (168)
Group 1	T1	DNA 4 mg IM*	DNA 4 mg IM*	DNA 4mg IM*	rAD $1x10^{10}$ PU IM**
Group 2	C1	DNA Placebo 1 ml IM*	DNA Placebo 1 ml IM*	DNA Placebo 1 ml IM*	rAD Placebo 1 ml IM**

*Administered by Biojector® as one 1 mL injection in either deltoid
**Administered with a needle and syringe as one 1 mL injection in either deltoid

Group 1

T1: VRC-HIVDNA016-00-VP 4 mg administered as 1 mL IM (via Biojector®) in either deltoid at Months 0, 1, and 2

AND

VRC-HIVADV014-00-VP $1x10^{10}$ PU administered as 1 mL IM in either deltoid at Month 6

Group 2

C1: Placebo for VRC-HIVDNA016-00-VP (PBS) 1 mL IM (via Biojector®) in either deltoid at Months 0, 1 and 2

AND

Placebo for VRC-HIVADV014-00-VP (labeled as VRC-DILUENT013-DIL-VP) 1 mL IM in either deltoid at Month 6

13.2 Study product formulation and preparation

See the Investigator's Brochure for further information about study products.

13.2.1 VRC-HIVDNA016-00-VP (DNA)

VRC-HIVDNA016-00-VP is manufactured by Vical Incorporated (San Diego, CA). The product is formulated in phosphate buffered saline (PBS), pH 7.2. The vaccine is provided as a 4 mg/mL solution in 2 mL single use glass vials containing 1.2 mL of a clear, colorless,

FOR REVIEW ONLY

sterile, isotonic solution. The product must be stored frozen (at -20° C or colder). Vials should not be refrozen after thawing.

To prepare, the pharmacist will remove 1 vial of DNA 4 mg/mL from the freezer and allow to equilibrate to room temperature. Swirl the contents gently. Using aseptic technique, aseptically withdraw 1 mL of the DNA (4mg/ml) from the vial into the Biojector® syringe and cap the syringe. Each syringe should be labeled as directed in the Study Specific Procedures. The injection should be given as soon as possible after preparation.

Any unused portion of entered vials and expired pre-filled syringes should be disposed of in a biohazard container and incinerated or autoclaved.

13.2.2 DNA Placebo (phosphate buffered saline, PBS)

Phosphate buffered saline (PBS) is manufactured by Bell-More Labs, Inc. (Hampstead, MD) for use as the placebo. The placebo is provided as a 2.4 mL of a clear, colorless, isotonic solution with a pH of 7.2. The product must be stored at controlled room temperature (15° to 30° C).

To prepare, the pharmacist will remove 1 vial of Placebo (PBS) and swirl the contents gently. Using aseptic technique, aseptically withdraw 1 mL of the PBS from the vial into the Biojector® syringe and cap the syringe. Each syringe should be labeled as directed in the Study Specific Procedures. The injection should be given as soon as possible after preparation.

Any unused portion of entered vials and expired pre-filled syringes should be disposed of in a biohazard container and incinerated or autoclaved.

13.2.3 VRC-HIVADV014-00-VP (multiclade HIV-1 recombinant adenoviral vector vaccine, rAD)

VRC-HIVADV014-00-VP is manufactured by GenVec Incorporated (Gaithersburg, MD) at a contract maufacturer, Molecular Medicine (San Diego, CA). The vaccine is supplied as a $1x10^{10}$ PU/mL solution in a 3 mL sterile glass vial containing 1.2 mls of a clear, colorless, sterile, isotonic solution. Although the vial label notes a storage temperature of -10° to -25° C, the product may be stored at temperatures as low as -30° C.

To prepare, the pharmacist will remove one vial of VRC-HIVADV014-00-VP $1x10^{10}$ PU/mL from the freezer and allow to equilibrate to room temperature. Using aseptic technique, withdraw 1 mL of the study product into a syringe. The study agent must be administered within 4 hours of removal from the freezer.

Any unused portion of entered vials and expired pre-filled syringes should be disposed of in a biohazard container and incinerated or autoclaved.

13.2.4 VRC-DILUENT013-DIL-VP (final formulation buffer, FFB, placebo)

Final Formulation Buffer (FFB) provided by GenVec Incorporated for use as the placebo is manufactured by BioWhittaker (Frederick, MD) and filled by Molecular Medicine (San Diego, CA). It is composed of sodium chloride, Tris buffer, trehalose•$2H_20$ (low endotoxin), magnesium chloride•$6H_20$, monoleate (Tween 80) and water for injection (WF1). It is provided as a 1.2 mL clear, colorless, isotonic solution with a pH of 7.2. It should be stored refrigerated (2-8 ° C).

To prepare, the pharmacist will remove one vial of VRC-DILUENT013-DIL-VP (placebo) from the refrigerator and allow to equilibrate to room temperature. Using aseptic technique,

FOR REVIEW ONLY

withdraw 1 mL of the study product into a syringe. The study agent must be administered within 4 hours of removal from the refrigerator.

Any unused portion of entered vials and expired pre-filled syringes should be disposed of in a biohazard container and incinerated or autoclaved..

13.2.5 Labeling procedures to preserve blinding

The pharmacist will prepare all doses for administration and dispense to the clinic. In order to preserve blinding, the pharmacist will place a yellow overlay on the syringes containing VRC-HIVADV014-00-VP or placebo.

13.3 Study product administration

All injections should be administered in either deltoid. It is not necessary to use the same deltoid at each visit. At sites where registered pharmacists are legally authorized to administer drug, the HVTU may choose to have the HVTU pharmacist administer the injections.

13.3.1 VRC-HIVDNA016-00-VP (DNA) vaccine or placebo (PBS)

A 1 mL injection of DNA vaccine or DNA placebo will be administered IM using the Biojector® at Months 0, 1, and 2.

The Biojector® 2000 Needle-Free Injection Management System™ will be used as directed by the Biojector® company. Neither the material being injected nor injection site skin preparation require deviation from standard procedures. The injection site is disinfected and the area allowed to dry completely. The skin around the injection site is held firmly while the syringe is placed against the injection site at a 90° angle. The actuator is pressed and the material is released into the muscle and held firmly for 3 seconds. After the injection, the site is covered with a sterile covering and pressure applied with 3 fingers for 1 minute. Biojector® utilizes sterile, single-use syringes for variable dose, up to 1 mL, medication administration. The study agent is delivered under pressure by a compressed CO_2 gas cartridge that is stored inside the Biojector®. When the Biojector's actuator is depressed, CO_2 is released, causing the plunger to push the study agent out of the sterile syringe through the skin and into the underlying tissue. The study agent is expelled through a micro-orifice at high velocity in a fraction of a second to pierce the skin. The CO_2 does not come in contact with the injectate and the syringe design prevents any back splatter or contamination of the device by tissue from the subject.

13.3.2 VRC-HIVADV014-00-VP (rAD vector) or placebo (labeled as VRC-DILUENT013-DIL-VP)

All injections are to be given intramuscularly into either deltoid muscle as a volume of 1 mL using a 21 gauge needle with a length of 1 or 1-1/2 inch (depending on subject arm size).

When preparing a dose in a syringe and administering the dose, consideration should be given to the volume of solution that may remain in the needle after the dose is administered. The pharmacy and clinic staff are encouraged to work together to administer the dose specified in the protocol.

FOR REVIEW ONLY

13.4 Study product acquisition

Study products will be provided by the Vaccine Research Center, National Institutes of Allergy and Infectious Diseases, NIH. The Biojector® and the Biojector® syringes will be provided by Bioject Inc.

At US-HVTUs the pharmacist can obtain study products from the DAIDS Clinical Research Products Management Center (CRPMC) by following the ordering procedures given in the section on Study Product Control in *Pharmacy Guidelines and Instructions for DAIDS Clinical Trials Networks* manual.

At non-US HVTUs the pharmacist can obtain study products from the DAIDS Clinical Research Products Management Center (CRPMC). Once a non-US HVTU is registered for the study and all required documents have been received by DAIDS Pharmaceutical Affairs Branch, the Pharmacist can order product by following the procedures given in the *HVTN 204 Study Specific Procedures (SSP).*

13.5 Pharmacy records

The HVTU pharmacist is required to maintain complete records of all study products received from the CRPMC and subsequently dispensed. All unused study products must be returned to the CRPMC after the study is completed or terminated unless otherwise instructed by the CRPMC. The procedures are included in the sections on Study Product Control and Drug Dispensing in *Pharmacy Guidelines and Instructions for DAIDS Clinical Trials Networks*.

The pharmacist of record is responsible for maintaining randomization codes and randomization confirmation notices for each participant in a secure manner.

FOR REVIEW ONLY

14 Safety monitoring and review

14.1 Assessing reactogenicity

Reactogenicity assessments are performed for all participants following each injection. HVTU staff will assess reactogenicity according to standard HVTN procedures as specified in the HVTN MOP (Study Operations>Safety Assessment>Reactogenicity). Any procedures which vary from the HVTN standard will be defined in *HVTN 204 Study Specific Procedures*.

The reactogenicity assessment period is for 3 days following the injection. Participants are instructed to record symptoms using a post-vaccination symptom log and contact the site daily during this reactogenicity assessment period. Clinic staff will follow new or unresolved reactogenicity symptoms present at Day 3 to resolution. The schedule is shown in Table 14-1.

Assessments to be performed:

- Systemic symptoms: body temperature, malaise and/or fatigue, myalgia, headache, chills, arthralgia, nausea, vomiting
- Local symptoms (proximal to injection site): pain, tenderness
- Vaccine-related lesions: erythema, induration
- Axillary lymph nodes (required only when reactogenicity assessments are performed by HVTU staff): lymph node tenderness, enlargement

Table 14-1 Schedule of reactogenicity assessments

Day	Time	Performed by
0[a]	Baseline: before injection	HVTU staff
	Early: 25 to 45 minutes after injection	HVTU staff
	Between early assessment and 11:59pm Day 0	HVTU staff or participant
1	Between 12:00am and 11:59pm Day 1	HVTU staff or participant
2	Between 12:00am and 11:59pm Day 2	HVTU staff or participant
3[b]	Between 12:00am and 11:59pm Day 3	HVTU staff or participant

[a]Day of injection
[b]New or unresolved reactogenicity symptoms present on Day 3 are followed until resolution

Participants are encouraged to contact the clinic for events that arise during the period between vaccination and the scheduled visit 14 days following vaccination.

14.2 Grading of adverse events

Local and systemic signs and symptoms are assessed and graded based on the *Division of AIDS Table for Grading the Severity of Adult and Pediatric Adverse Events,* Version 1, December, 2004.

14.3 Adverse event reporting and safety pause/AE review rules

All adverse events are reported to the SDMC on the appropriate case report form (CRF) according to procedures in the HVTN MOP (Study Operations>Safety Assessments>Adverse Experiences). The mechanism of reporting certain Grade 3 and higher grade vaccine-related

FOR REVIEW ONLY

symptoms and adverse events to the SDMC clinical affairs staff is depicted in Table 14-2. The mechanism of reporting SAEs and other events meeting expedited adverse event (EAE) reporting requirements to DAIDS is specified in Section 14.4.

14.3.1 Adverse events to which safety pause/AE review rules apply

The adverse events applying toward a safety pause or Protocol Safety Review Team (PSRT) AE review are shown in Table 14-2. In order to be counted toward a safety pause or PSRT AE review, adverse events must be vaccine related. Symptoms reported on a reactogenicity CRF are assumed by the SDMC to be vaccine related.

14.3.2 PSRT AE review and safety pause

For all safety pauses, the SDMC Clinical Affairs staff notifies the HVTN 204 PSRT, DAIDS Pharmaceutical Affairs Branch (PAB), Regulatory Compliance Center (RCC)/Regulatory Affairs Branch (RAB), and participating HVTUs that all study vaccinations are held until further notice; DAIDS notifies the US FDA. When an immediate safety pause is triggered, the SDMC Clinical Affairs staff also notifies the HVTN SMB.

Vaccinations may be suspended for safety concerns other than those described in Table 14-2, or before pause rules are met, if in the judgment of the PSRT, participant safety may be threatened.

Adverse events that do not prompt a safety pause of a PSRT AE review are routinely reviewed by the PSRT (Section 14.7.2)

Table 14-2 Adverse event notification and safety pause/AE review rules

Rule	Toxicity	Symptom/AE	Vaccine relatedness	HVTU action	SDMC action	Criterion for SDMC action	Criterion for each subsequent SDMC action
1	Grade 4	Any lab abnormality, adverse event, local or systemic reactogenicity symptom	Probably or definitely	Immediate notification	Immediate pause	1 ppt with AE/symptom at specified grade	1 additional ppt with the same AE/symptom at specified grade
2	Grade 4	Any lab abnormality, fever, vomiting, localized injection site reaction, or other clinical adverse event	Possibly	Expedited notification	Expedited PSRT AE review	1 ppt with AE/symptom at specified grade	1 additional ppt with the same AE/symptom at specified grade
3	Grade 3	Any lab abnormality, fever, vomiting, localized injection site reaction, or other clinical adverse event (other than subjective reactogenicity symptom)	Possibly, probably, or definitively	Expedited notification	Expedited PSRT AE review	1 ppt with AE/symptom at specified grade	1 additional ppt with the same AE/symptom at specified grade

For AE descriptions and grading, see *The Division of AIDS Table for Grading the Severity of Adult and Pediatric Adverse Events* (DAIDS AE Grading Table), Version 1.0, December, 2004.

Immediate: HVTU notifies SDMC Clinical Affairs staff immediately by pager or live phone (not voice mail) and email, and faxes completed forms as soon as possible. SDMC Clinical Affairs staff notifies PSRT, PAB, RCC/RAB, HVTN SMB, and participating HVTUs as soon as possible that vaccinations are held until further notice.

Expedited: HVTU faxes completed forms within 24 hours of receiving notice of event. SDMC Clinical Affairs staff notifies the PSRT that a PSRT AE review is needed. If a PSRT AE review cannot be completed within 48 hours from the time SDMC receives notification, an automatic safety pause occurs.

Phone/pager number and email address listed in *HVTN 204 Study Specific Procedures.*

FOR REVIEW ONLY

14.3.3 Review and notification following safety pause

The PSRT reviews safety data and decides whether the pause can be lifted or permanent discontinuation of vaccination is appropriate, consulting the SMB and the US FDA if necessary. DAIDS will consult with the US FDA for all immediate safety pauses. SDMC Clinical Affairs staff notifies participating HVTUs, PAB, and RCC/RAB of the decision regarding resumption or discontinuation of study vaccinations. SDMC Clinical Affairs staff also notifies the HVTN SMB, and DAIDS notifies the FDA, if these groups have not been informed earlier.

Each HVTU is responsible for submitting to its IRB/IEC and any local regulatory authority protocol-related safety information (such as IND safety reports, notification of vaccine holds due to the pause rules, etc.) as required.

FOR REVIEW ONLY

14.4 Expedited adverse event reporting to DAIDS

The expedited adverse event (EAE) reporting requirements and definitions for this study and the methods for expedited reporting of adverse events (AEs) to the DAIDS Regulatory Compliance Center (RCC) Safety Office are defined in "The Manual for Expedited Reporting of Adverse Events to DAIDS" (DAIDS EAE Manual), **dated May 6, 2004**. The DAIDS EAE Manual is available on the RCC website: http://rcc.tech-res-intl.com/.

AEs reported on an expedited basis must be documented on the DAIDS Expedited Adverse Event Reporting Form (EAE Reporting Form) available on the RCC website: http://rcc.tech-res-intl.com.

HVTUs submit the form to DAIDS through the RCC and simultaneously send a copy to SDMC Clinical Affairs staff.

14.4.1 EAE reporting level

This study uses the Standard Level of expedited AE reporting as defined in the DAIDS EAE Manual.

14.4.2 Study agents for expedited reporting to DAIDS

The study agents that must be considered in determining relationships of AEs requiring expedited reporting to DAIDS are: VRC-HIVDNA-016-00-VP/placebo and VRC-HIVADV014-00-VP/placebo.

14.4.3 Grading severity of events

The *Division of AIDS Table for Grading the Severity of Adult and Pediatric Adverse Events* (DAIDS AE Grading Table), Version 1, December, 2004 must be used and is available on the RCC website at http://rcc.tech-res-intl.com/.

14.4.4 EAE reporting periods

AEs must be reported on an expedited basis at the Standard Level during the Protocol-defined EAE Reporting Period, which is the entire study duration for an individual subject (from study enrollment until study completion or discontinuation of the subject from study participation for any reason).

After the end of the Protocol-defined EAE Reporting Period stated above, sites must report serious, unexpected, clinical suspected adverse drug reactions if the study site staff becomes aware of the event on a passive basis, i.e., from publicly available information.

FOR REVIEW ONLY

14.5 Participant departure from schedule of vaccinations

14.5.1 Delaying vaccinations for a participant

Under certain circumstances a participant's scheduled vaccination may need to be held. These include but are not limited to the following:

- Receipt of live attenuated vaccines within 30 days prior to vaccination
- Receipt of medically indicated subunit or killed vaccines (e.g., influenza, pneumococcal) within 14 days prior to vaccination
- Use of other investigational research agents within 30 days prior to vaccination
- Receipt of blood products or immunoglobulin within 45 days prior to vaccination
- Prevaccination abnormal vital signs or clinical symptoms that may mask assessment of vaccine reaction

Vaccinations cannot be administered outside the window period specified in the *HVTN 204 Study Specific Procedures*.

14.5.2 Stopping vaccinations for a participant

Under certain circumstances, an individual participant's vaccinations will be stopped. Such participants should be encouraged to participate in follow-up visits and all protocol-related procedures (unless medically contraindicated) per the protocol for the remainder of the trial. Specific events that will result in stopping a participant's vaccination schedule include the following:

- Clinically significant condition (i.e., a condition that affects the immune system or for which continued vaccinations and/or blood draws may pose additional risk), including but not limited to the following:
  - HIV infection (requires termination from the study)
  - Pregnancy (regardless of outcome)
  - Any Grade 4 local or systemic symptom, lab abnormality, or adverse experience, that is subsequently confirmed to be *possibly*, *probably*, or *definitely* related to vaccination
  - Any Grade 3 lab abnormality or other clinical adverse experience (exception: fever or vomiting and subjective local and systemic symptoms) that is subsequently confirmed to be *possibly*, *probably*, or *definitely* related to vaccination
  - Type 1 hypersensitivity associated with vaccination
- Inability to receive vaccination within the specified period for the designated study visit (see *HVTN 204 Study Specific Procedures*)
- Investigator determination in consultation with the study chair and statistician, e.g., for repeated nonadherence to protocol requirements

14.5.3 Participant termination from the study

Under certain circumstances, an individual participant may be terminated from participation in this study. Specific events that will result in early termination include:

- Participant refused to participate further
- Participant relocated to an area without a nearby HVTU and remote follow-up is not possible

- HVTU determined that the participant is lost to follow-up
- Participant becomes HIV-infected

14.6 Study termination (for all participants)

This study may be terminated by the determination of the HVTN 204 Protocol Safety Review Team, HVTN Safety Monitoring Board, US FDA, US NIH, vaccine developer, or regulatory authority (e.g., IRB or IEC), as well as local regulatory authority for non-US sites. See Section 14.7 for discussion of the safety review process.

14.7 HVTN review of cumulative safety data

Routine safety reviews proceed from a standardized set of protocol-specific safety data reports. These reports are produced by SDMC and annotated with queries to the HVTU and additional notes. Events are tracked by the internal reports until resolution. Other reports, containing the queries and notes, are distributed to the HVTN 204 Protocol Safety Review Team. The following reports are produced:

- Clinical quality control
- Safety review
- Preexisting conditions
- Adverse events (AEs) requiring review
- Adverse event/concomitant medication
- WBC/differential
- Safety summary

More detailed information regarding the contents and distribution of these reports can be found in the HVTN MOP.

14.7.1 Daily review

Blinded daily safety reviews are routinely conducted by the SDMC clinical affairs staff for SAEs and events that meet safety pause criteria.

14.7.2 Weekly review

During the injection phase of the trial, the SDMC clinical affairs staff and the HVTN 204 Protocol Safety Review Team (PSRT) review clinical safety reports on a weekly basis and conduct calls to review the data as appropriate. After the injections and the final 2-week safety visits are completed, less frequent safety reviews may be conducted at the discretion of the HVTN 204 PSRT. The SDMC clinical affairs staff reviews reports of all clinical values that fall outside of the standard HVTN safety parameters (see HVTN MOP [Study Operations>Standard Reports>Clinical Safety Review>Weekly Safety Review Reports]). Values identified during the review that are considered questionable, inconsistent, or unexplained are referred to the HVTU clinic coordinator for verification.

The HVTN 204 PSRT is composed of the following required members:

- Protocol chair and co-chair
- Protocol team leader

FOR REVIEW ONLY

- Core medical monitor
- SDMC clinical affairs staff member
- DAIDS medical officer

Additional members include a vaccine developer representative and a representative from the RV 172 and IAVI V001 teams. The HVTN 204 Protocol Team clinic coordinator and protocol specialist may also be included at the request of the HVTN 204 PSRT.

14.7.3 Quarterly review

In addition to the detailed clinical monitoring reports discussed above, protocol-specific summary reports of reactogenicity and AE data are provided to the HVTN Phase I/II Committee in a blinded fashion approximately once per quarter.

14.7.4 Safety Monitoring Board review

The HVTN safety monitoring board is composed of the following individuals:

- SMB Chair
- DAIDS Medical Officer representative
- Non-US representative
- US representative
- Statistician
- Clinician
- HVTN director

Members of the HVTN Safety Monitoring Board are not directly affiliated with the protocols under review. The safety monitoring board will review unblinded safety data approximately every 4 months. This review is designed to provide confirmation with respect to ad hoc review requests as well as increase overall sensitivity for detecting potential safety problems by looking across multiple protocols that use the same or similar vaccine candidates. The review consists of evaluation of unblinded safety data, including comparisons of adverse experiences in vaccine and placebo recipients in aggregate, as well as review of individual SAE reports. The Safety Monitoring Board will conduct additional special reviews at the request of the HVTN 204 Protocol Safety Review Team.

FOR REVIEW ONLY

References

1. UNAIDS, WHO. AIDS epidemic update. **2002**;UNAIDS/02.46E:1-36.

2. Centers for Disease Control and Prevention. HIV/AIDS surveillance report. **2001**;1-48.

3. UNAIDS. Report on the global HIV/AIDS epidemic. **2002**;UNAIDS/02.26E.

4. Fauci AS. The AIDS epidemic--considerations for the 21st century. N Engl J Med **1999**;341:1046-50.

5. Varmus H, Nathanson N. Science and the control of AIDS. Science **1998**;280:1815.

6. McCutchan FE. Understanding the genetic diversity of HIV-1. AIDS **2000**;14 Suppl 3:S31-S44.

7. Kuiken CL, Lukashov VV, Baan E, Dekker J, Leunissen JA, Goudsmit J. Evidence for limited within-person evolution of the V3 domain of the HIV-1 envelope in the amsterdam population. AIDS **1996**;10:31-7.

8. Moore JP, Parren PW, Burton DR. Genetic subtypes, humoral immunity, and human immunodeficiency virus type 1 vaccine development. J Virol **2001**;75:5721-9.

9. Shankarappa R, Margolick JB, Gange SJ, et al. Consistent viral evolutionary changes associated with the progression of human immunodeficiency virus type 1 infection. J Virol **1999**;73:10489-502.

10. Nixon DF, Townsend AR, Elvin JG, Rizza CR, Gallwey J, McMichael AJ. HIV-1 gag-specific cytotoxic T lymphocytes defined with recombinant vaccinia virus and synthetic peptides. Nature **1988**;336:484-7.

11. Emini, E. A. A potential HIV-1 vaccine using a replication-defective adenoviral vaccine vector. 9th conference of retroviruses and opportunistic infections. Seattle, **2002**.

12. Korber B, Brander C, Haynes B, Koup R, Kuiken C, Moore JP, Watkins DI. HIV molecular immunology 2000. Los Alamos, NM: Los Alamos National Laboratory, Theoretical Biology and Biophysics, **2000**.

13. Gaschen B, Taylor J, Yusim K, et al. Diversity considerations in HIV-1 vaccine selection. Science **2002**;296:2354-60.

14. Approaches to the development of broadly protective HIV vaccines: challenges posed by the genetic, biological and antigenic variability of HIV-1: Report from a meeting of the WHO-UNAIDS Vaccine Advisory Committee Geneva, 21-23 February 2000. AIDS **2001**;15:W1-W25.

15. Osmanov S, Pattou C, Walker N, Schwardlander B, Esparza J. Estimated global distribution and regional spread of HIV-1 genetic subtypes in the year 2000. J Acquir Immune Defic Syndr **2002**;29:184-90.

FOR REVIEW ONLY

16. Charo J, Lindencrona JA, Carlson LM, Hinkula J, Kiessling R. Protective efficacy of a DNA influenza virus vaccine is markedly increased by the coadministration of a Schiff base-forming drug. J Virol **2004**;78:11321-6.

17. Wang R, Doolan DL, Le TP, et al. Induction of antigen-specific cytotoxic T lymphocytes in humans by a malaria DNA vaccine. Science **1998**;282:476-80.

18. Le TP, Coonan KM, Hedstrom RC, et al. Safety, tolerability and humoral immune responses after intramuscular administration of a malaria DNA vaccine to healthy adult volunteers. Vaccine **2000**;18:1893-901.

19. Tacket CO, Roy MJ, Widera G, Swain WF, Broome S, Edelman R. Phase 1 safety and immune response studies of a DNA vaccine encoding hepatitis B surface antigen delivered by a gene delivery device. Vaccine **1999**;17:2826-9.

20. Kotsopoulou E, Kim VN, Kingsman AJ, Kingsman SM, Mitrophanous KA. A Rev-independent human immunodeficiency virus type 1 (HIV-1)-based vector that exploits a codon-optimized HIV-1 gag-pol gene. J Virol **2000**;74:4839-52.

21. zur Megede J, Chen MC, Doe B, et al. Increased expression and immunogenicity of sequence-modified human immunodeficiency virus type 1 gag gene. J Virol **2000**;74:2628-35.

22. Kim JJ, Yang JS, VanCott TC, et al. Modulation of antigen-specific humoral responses in rhesus macaques by using cytokine cDNAs as DNA vaccine adjuvants. J Virol **2000**;74:3427-9.

23. Sedegah M, Weiss W, Sacci JB, Jr., et al. Improving protective immunity induced by DNA-based immunization: priming with antigen and GM-CSF-encoding plasmid DNA and boosting with antigen-expressing recombinant poxvirus. J Immunol **2000**;164:5905-12.

24. Boyer JD, Kim J, Ugen K, et al. HIV-1 DNA vaccines and chemokines. Vaccine **1999**;17 Suppl 2:S53-S64.

25. Mascola JR, Nabel GJ. Vaccines for the prevention of HIV-1 disease. Curr Opin Immunol **2001**;13:489-95.

26. Barouch DH, Santra S, Schmitz JE, et al. Control of viremia and prevention of clinical AIDS in rhesus monkeys by cytokine-augmented DNA vaccination. Science **2000**;290:486-92.

27. MacGregor RR, Boyer JD, Ugen KE, et al. First human trial of a DNA-based vaccine for treatment of human immunodeficiency virus type 1 infection: safety and host response. J Infect Dis **1998**;178:92-100.

28. MacGregor RR, Boyer JD, Ciccarelli RB, Ginsberg RS, Weiner DB. Safety and immune responses to a DNA-based human immunodeficiency virus (HIV) type I env/rev vaccine in HIV-infected recipients: follow-up data. J Infect Dis **2000**;181:406.

29. Boyer JD, Cohen AD, Vogt S, et al. Vaccination of seronegative volunteers with a human immunodeficiency virus type 1 env/rev DNA vaccine induces antigen-specific proliferation and lymphocyte production of beta-chemokines. J Infect Dis **2000**;181:476-83.

FOR REVIEW ONLY

30. Mwau M, Cebere I, Sutton J, et al. A human immunodeficiency virus 1 (HIV-1) clade A vaccine in clinical trials: stimulation of HIV-specific T-cell responses by DNA and recombinant modified vaccinia virus Ankara (MVA) vaccines in humans. J Gen Virol **2004**;85:911-9.

31. Isaacs, R. Impact of pre-existing immunity on the immunogenicity of adenovirus serotype 5-based vaccines. AIDS Vaccine 2004. Lausanne, Switzerland, **2004**.

32. Shiver, J. Development of an anti-HIV-1 vaccine based on replication defective adenovirus. Keystone Symposium on HIV Vaccine Development. Whistler, British Columbia, Canada, **2004**.

33. DeRose, R., Dale, C. J., Stratov, I., Chea, S., Purcell, D., Ramshaw, I. A., Thomson, S., Boyle, D. B., Coupar, B., Ramsay, A. J., French, R., Law, M., Emery, S., Cooper, D. A., Kent, S. J. Efficacy of DNA and fowlpox prime/boost vaccines for HIV-1 and SHIV. World AIDS Conference. Bangkok, Thailand, **2004**.

34. Plotkin S, Orenstein WA. Vaccines. Philadelphia: W.B. Saunders Co., **1999**;1230.

35. Schwartz AR, Togo Y, Hornick RB. Clinical evaluation of live, oral types 1, 2, and 5 adenovirus vaccines. Am Rev Respir Dis **1974**;109:233-9.

36. Rosenbaum MJ, Poudstone JW, Edwards EA. Acceptability and antigenicity of adjuvant soluble sub-unit adenovirus vaccines in navy recruits. Mil Med **1976**;141:383-8.

37. Rosenbaum MJ, Edwards EA, Hoeffler DF CAPT. Recent experiences with live adenovirus vaccines in navy recruits. Mil Med **1975**;140:251-7.

38. Gaydos CA, Gaydos JC. Adenovirus vaccines in the U.S. military. Mil Med **1995**;160:300-4.

39. Harvey BG, Leopold PL, Hackett NR, et al. Airway epithelial CFTR mRNA expression in cystic fibrosis patients after repetitive administration of a recombinant adenovirus. J Clin Invest **1999**;104:1245-55.

40. Bellon G, Michel-Calemard L, Thouvenot D, et al. Aerosol administration of a recombinant adenovirus expressing CFTR to cystic fibrosis patients: a phase I clinical trial. Hum Gene Ther **1997**;8:15-25.

41. Hay JG, McElvaney NG, Herena J, Crystal RG. Modification of nasal epithelial potential differences of individuals with cystic fibrosis consequent to local administration of a normal CFTR cDNA adenovirus gene transfer vector. Hum Gene Ther **1995**;6:1487-96.

42. Harvey BG, Worgall S, Ely S, Leopold PL, Crystal RG. Cellular immune responses of healthy individuals to intradermal administration of an E1-E3- adenovirus gene transfer vector. Hum Gene Ther **1999**;10:2823-37.

43. Rosengart TK, Lee LY, Patel SR, et al. Six-month assessment of a phase I trial of angiogenic gene therapy for the treatment of coronary artery disease using direct intramyocardial administration of an adenovirus vector expressing the VEGF121 cDNA. Ann Surg **1999**;230:466-70.

44. Rosengart TK, Lee LY, Patel SR, et al. Angiogenesis gene therapy: phase I assessment of direct intramyocardial administration of an adenovirus vector expressing VEGF121 cDNA to

FOR REVIEW ONLY

individuals with clinically significant severe coronary artery disease. Circulation **1999**;100:468-74.

45. Sterman DH, Treat J, Litzky LA, et al. Adenovirus-mediated herpes simplex virus thymidine kinase/ganciclovir gene therapy in patients with localized malignancy: results of a phase I clinical trial in malignant mesothelioma. Hum Gene Ther **1998**;9:1083-92.

46. Clayman GL, Frank DK, Bruso PA, Goepfert H. Adenovirus-mediated wild-type p53 gene transfer as a surgical adjuvant in advanced head and neck cancers. Clin Cancer Res **1999**;5:1715-22.

47. Swisher SG, Roth JA, Nemunaitis J, et al. Adenovirus-mediated p53 gene transfer in advanced non-small-cell lung cancer. J Natl Cancer Inst **1999**;91:763-71.

48. Gahery-Segard H, Molinier-Frenkel V, Le Boulaire C, et al. Phase I trial of recombinant adenovirus gene transfer in lung cancer. Longitudinal study of the immune responses to transgene and viral products. J Clin Invest **1997**;100:2218-26.

49. Tursz T, Cesne AL, Baldeyrou P, et al. Phase I study of a recombinant adenovirus-mediated gene transfer in lung cancer patients. J Natl Cancer Inst **1996**;88:1857-63.

50. Harvey BG, Maroni J, O'Donoghue KA, et al. Safety of local delivery of low- and intermediate-dose adenovirus gene transfer vectors to individuals with a spectrum of morbid conditions. Hum Gene Ther **2002**;13:15-63.

51. Harvey BG, Hackett NR, El Sawy T, et al. Variability of human systemic humoral immune responses to adenovirus gene transfer vectors administered to different organs. J Virol **1999**;73:6729-42.

52. Rajagopalan S, Trachtenberg J, Mohler E, et al. Phase I study of direct administration of a replication deficient adenovirus vector containing the vascular endothelial growth factor cDNA (CI-1023) to patients with claudication. Am J Cardiol **2002**;90:512-6.

53. Crystal RG, Harvey BG, Wisnivesky JP, et al. Analysis of risk factors for local delivery of low- and intermediate-dose adenovirus gene transfer vectors to individuals with a spectrum of comorbid conditions. Hum Gene Ther **2002**;13:65-100.

54. Raper SE, Yudkoff M, Chirmule N, et al. A pilot study of in vivo liver-directed gene transfer with an adenoviral vector in partial ornithine transcarbamylase deficiency. Hum Gene Ther **2002**;13:163-75.

55. Assessment of adenoviral vector safety and toxicity: report of the National Institutes of Health Recombinant DNA Advisory Committee. Hum Gene Ther **2002**;13:3-13.

56. Varnavski AN, Zhang Y, Schnell M, et al. Preexisting immunity to adenovirus in rhesus monkeys fails to prevent vector-induced toxicity. J Virol **2002**;76:5711-9.

57. Yang Y, Wilson JM. Clearance of adenovirus-infected hepatocytes by MHC class I-restricted CD4+ CTLs in vivo. J Immunol **1995**;155:2564-70.

FOR REVIEW ONLY

58. Yang Y, Nunes FA, Berencsi K, Furth EE, Gonczol E, Wilson JM. Cellular immunity to viral antigens limits E1-deleted adenoviruses for gene therapy. Proc Natl Acad Sci U S A **1994**;91:4407-11.

59. Nunes FA, Furth EE, Wilson JM, Raper SE. Gene transfer into the liver of nonhuman primates with E1-deleted recombinant adenoviral vectors: safety of readministration. Hum Gene Ther **1999**;10:2515-26.

60. Lozier JN, Csako G, Mondoro TH, et al. Toxicity of a first-generation adenoviral vector in rhesus macaques. Hum Gene Ther **2002**;13:113-24.

61. Morral N, O'Neal WK, Rice K, et al. Lethal toxicity, severe endothelial injury, and a threshold effect with high doses of an adenoviral vector in baboons. Hum Gene Ther **2002**;13:143-54.

62. Fu TM, et al. Replication-incompetent recombinant adenovirus vector expressing SIV Gag elicits robust and effective cellular immune responses in rhesus macaques. AIDS Vaccine 2001, **2001**.

63. Tang A, et al. Evaluation of the immunogenicity of HIV-1 vaccine in baboons. AIDS Vaccine 2001, **2001**.

64. Casimiro D, et al. Preclinical evaluation of gene-delivery-based HIV-1 vaccine in nonhuman primates. AIDS Vaccine 2001, **2001**.

65. Shiver JW, Fu TM, Chen L, et al. Replication-incompetent adenoviral vaccine vector elicits effective anti-immunodeficiency-virus immunity. Nature **2002**;415:331-5.

66. Sullivan NJ, Sanchez A, Rollin PE, Yang ZY, Nabel GJ. Development of a preventive vaccine for Ebola virus infection in primates. Nature **2000**;408:605-9.

67. Yang ZY, Wyatt LS, Kong WP, Moodie Z, Moss B, Nabel GJ. Overcoming immunity to a viral vaccine by DNA priming before vector boosting. J Virol **2003**;77:799-803.

68. Emini, E. A. Ongoing Development and Evaluation of a Potential HIV-1 Vaccine Using a Replication-Defective Adenoviral Vector. Keystone Conference on HIV Vaccines. Banff, **2003**.

69. Chirmule N, Propert K, Magosin S, Qian Y, Qian R, Wilson J. Immune responses to adenovirus and adeno-associated virus in humans. Gene Ther **1999**;6:1574-83.

70. Hofmann C, Loser P, Cichon G, Arnold W, Both GW, Strauss M. Ovine adenovirus vectors overcome preexisting humoral immunity against human adenoviruses in vivo. J Virol **1999**;73:6930-6.

71. Kostense, S, Vogel, S., Lemckert, A., Ophorst, O., Koudstaal, W., Sprangers, M., Goudsmit, J., Havenga, M. Adenovirus serotype 35-based vaccination in the absence or presence of serotype 5 specific immunity. Keystone Conference on HIV Vaccines. Banff, **2003**.

72. Vogels R, Zuijdgeest D, van Rijnsoever R, et al. Replication-deficient human adenovirus type 35 vectors for gene transfer and vaccination: efficient human cell infection and bypass of preexisting adenovirus immunity. J Virol **2003**;77:8263-71.

FOR REVIEW ONLY

73. Seshidhar RP, Ganesh S, Limbach MP, et al. Development of adenovirus serotype 35 as a gene transfer vector. Virology **2003**;311:384-93.

74. Schmitz H, Wigand R, Heinrich W. Worldwide epidemiology of human adenovirus infections. Am J Epidemiol **1983**;117:455-66.

75. Nwanegbo E, Gao W, Gambotto A, Rowe D, Robbins P, Sun H, Whittle H, Vardas E. Prevalence of neutralizing antibody to adenoviral serotype 5 and 35 in the Gambia, South Africa, and United States adult populations. AIDS Vaccine 2003. New York, **2003**.

76. Mast TC, Gupta S, Nikas A, Kierstead L, Kallas EG, Mbewe B, Pitisuttithum P, Schecter M, Vardas E, Wolfe N, Coplan P, Aste-Amezaga M, Shiver J, Emini EA, Straus WL. International epidemiology of human preexisting anti-adenovirus type-5 (Ad5) antibodies in areas representative of potential HIV vaccine trial sites. AIDS Vaccine 2003. Bangkok, Thailand, **2004**.

77. Amara RR, Villinger F, Altman JD, et al. Control of a mucosal challenge and prevention of AIDS by a multiprotein DNA/MVA vaccine. Science **2001**;292:69-74.

78. Casimiro DR, Chen L, Fu TM, et al. Comparative immunogenicity in rhesus monkeys of DNA plasmid, recombinant vaccinia virus, and replication-defective adenovirus vectors expressing a human immunodeficiency virus type 1 gag gene. J Virol **2003**;77:6305-13.

79. Chakrabarti BK, Kong WP, Wu BY, et al. Modifications of the human immunodeficiency virus envelope glycoprotein enhance immunogenicity for genetic immunization. J Virol **2002**;76:5357-68.

80. Williams J, Fox-Leyva L, Christensen C, et al. Hepatitis A vaccine administration: comparison between jet-injector and needle injection. Vaccine **2000**;18:1939-43.

81. Hoke CH, Jr., Egan JE, Sjogren MH, et al. Administration of hepatitis A vaccine to a military population by needle and jet injector and with hepatitis B vaccine. J Infect Dis **1995**;171 Suppl 1:S53-S60.

82. Manam S, Ledwith BJ, Barnum AB, et al. Plasmid DNA vaccines: tissue distribution and effects of DNA sequence, adjuvants and delivery method on integration into host DNA. Intervirology **2000**;43:273-81.

83. Wang R, Epstein J, Baraceros FM, et al. Induction of CD4(+) T cell-dependent CD8(+) type 1 responses in humans by a malaria DNA vaccine. Proc Natl Acad Sci U S A **2001**;98:10817-22.

84. Rogers WO, Baird JK, Kumar A, et al. Multistage multiantigen heterologous prime boost vaccine for Plasmodium knowlesi malaria provides partial protection in rhesus macaques. Infect Immun **2001**;69:5565-72.

85. Epstein JE, Gorak EJ, Charoenvit Y, et al. Safety, tolerability, and lack of antibody responses after administration of a PfCSP DNA malaria vaccine via needle or needle-free jet injection, and comparison of intramuscular and combination intramuscular/intradermal routes. Hum Gene Ther **2002**;13:1551-60.

FOR REVIEW ONLY

86. Rogers WO, Weiss WR, Kumar A, et al. Protection of rhesus macaques against lethal Plasmodium knowlesi malaria by a heterologous DNA priming and poxvirus boosting immunization regimen. Infect Immun **2002**;70:4329-35.

87. Seth P. Adenoviruses: basic biology to gene therapy. Austin, TX: RG Landes Co., **1999**.

88. Brough DE, Lizonova A, Hsu C, Kulesa VA, Kovesdi I. A gene transfer vector-cell line system for complete functional complementation of adenovirus early regions E1 and E4. J Virol **1996**;70:6497-501.

89. Hudgens MG. Estimating cumulative probabilities from incomplete longitudinal binary responses with application to HIV vaccine trials. Statistics in Medicine **2003**;22:463-79.

FOR REVIEW ONLY

Protocol history

The Protocol Team may modify the original version of the protocol. Modifications are made to HVTN protocols via clarification memos, letters of amendment, or full protocol amendments. HVTN protocols are modified and distributed according to the standard HVTN procedures as described in the HVTN MOP (Organization and Policy>Vaccine Selection and Protocol Development).

The table below describes the version history of, and modifications to, Protocol HVTN 204.

Protocol history and modifications

Date	Protocol version	Protocol modification	Comment
24-May-05	Version 1	Original protocol	

Date	Protocol version	Protocol modification	Summary of modifications
2-Apr-08	Version 1.2 for RSA sites	Version 1.2 for RSA sites incorporates changes in LOA #3	Version 1.2 for RSA sites incorporates changes in LOA #3
2-Apr-08	Version 1 (1.1 for RSA sites)	Letter of Amendment 3	Item 1 Modified: Study duration expanded from 12 to 36 months for HIV testing and risk reduction counseling
			Item 2 Added: PBMC collection in a subset of participants from South Africa (RSA) and the United States (US)
			Item 3 Per items 1 and 2, added sample addendums #1 and #2 to Informed Consent Form
			Item 4 Clarified: EAE reporting period (section 14.4.4) to correctly define what AEs require expedited reporting
			Item 5 Split risk reduction/pregnancy prevention counseling procedure line in Appendix B (Procedures at HVTU)
			Item 6 Clarified: Appendix B (Procedures at HVTU) post study unblinding visit
			Item 7 Changed: Tube type for HIV diagnostic tests in Appendix A (Laboratory procedures)
5-Mar-07	Version 1 (1.1 for RSA sites)	Clarification Memo 6	Item 1 Clarified: Clinical safety parameters in Section 14.7.2, Weekly review (of cumulative safety data)
			Item 2 Added as footnote to Appendix A, Laboratory procedures: Clarification of blood volume required for end-of-study HIV testing at visit 13
			Item 3 Deleted: HVTN Director from SMB membership list in Section 14.7.4, Safety Monitoring Board review
8-Dec-06	Version 1 (1.1 for RSA sites)	Clarification Memo 5	Item 1 Clarified: Storage temperature range, manufacturer, and fill volume for DNA placebo (phosphate buffered saline, PBS) specified in section 13.2.2
29-Mar-	Version 1.1 for RSA sites	Version 1.1 for RSA sites clarifications pertaining	Version 1.1 for RSA sites clarifications pertaining specifically to RSA

FOR REVIEW ONLY

06		specifically to RSA	
8-Feb-06	Version 1	Clarification Memo 4	Item 1 Discontinued: Specimen collection of blood plasma at baseline for RNA HIV-1 PCR<br>Item 2 Replaced: Viral and Rickettsial Disease Laboratory (Richmond, California) by University of Washington Virology Specialty Laboratory (Seattle, Washington)<br>Item 3 Changed: Tube type for HIV RNA PCR specimens from PPT to EDTA
22-Dec-05	Version 1	Clarification Memo 3	Item 1 Clarification: Small post-vaccination papules do not require photographic documentation
27-Oct-05	Version 1	Clarification Memo 2	Item 1 Clarification of storage and preparation for VRC-DILUENT013-DIL-VP (final formulation buffer, FFB, placebo)<br>Item 2 Discontinued: Specimen collection for HIV DNA PCR testing<br>Item 3 Corrected: Filing instructions for Clarification Memo #1
25-Oct-05	Version 1	Letter of Amendment 2 (South Africa only)	Item 1 Added: Inclusion criterion (South Africa only): completion of at least 12 years of education
12-Sept-05	Version 1	Clarification Memo 1	Item 1 Corrections to Appendix A: Laboratory Procedures<br>Item 2 Hypertension exclusion criterion is clarified
2-Sept-05	Version 1	Letter of Amendment 1	Item 1 Post-vaccination skin lesions to be photographed by participating sites with participant consent
24-May-05	Version 1	Original protocol	NA

FOR REVIEW ONLY

APPENDICES

FOR REVIEW ONLY

Appendix A: Laboratory procedures

					Tube volume (mL)													
				Visit:	**1**	**2**	**3**	**4**	**5**	**6**	**7**	**8**	**9**	**10**	**11**	**12**	**13**	
				Day:	Screening	D0	D14	D28	D42	D56	D70	D168	D182	D196	D210	D288	D364	
				Month:		M0	M0.5	M1	M1.5	M2		M6		M7		M9.5	M12	
Procedure	Ship to	Assay location	Tube			**VAC1**		**VAC2**		**VAC3**		**VAC4**					**Final**	**Total**
Blood Collection																		
Screening or diagnostic assays																		
HIV screening test	Local Lab	Local Lab	SST		5	—	—	—	—	—	—	—	—	—	—	—	—	**5**
HIV diagnostic ELISA	Richmond/Local lab	Richmond/Local lab	SST		—	—	—	—	—	5	—	5	—	—	—	5	5	**20**
HIV RNA PCR	Richmond/Local lab	Richmond/Local lab	PPT		—	5	—	—	—	5	—	5	—	—	—	5	5	**25**
HIV DNA PCR	Richmond/Local lab	Richmond/Local lab	ACD		—	10	—	—	—	10	—	10	—	—	—	10	10	**50**
Syphilis test	Local Lab	Local Lab	SST		5	—	—	—	—	—	—	—	—	—	—	—	—	**5**
Safety labs																		
CBC/ Diff/ platelets	Local lab	Local lab	EDTA		5	—	5	—	5	—	5	—	5	—	—	5	5	**35**
ALT/Creatinine	Local lab	Local lab	SST		5	—	5	—	5	—	5	—	5	—	—	5	5	**35**
Immunogenicity assays																		
HLA typing	CSR	Duke			—	20	—	—	—	—	—	—	—	—	—	—	—	**20**
Humoral assays																		
HIV Neut. Antibody	CSR	Duke	SST		—	5	—	—	—	—	—	—	—	*5*	—	—	5	**15**
HIV binding ELISA	CSR	Duke	SST		—	5	—	—	5	—	5	5	5	*5*	—	5	5	**40**
HIV Binding /Neut	CSR	VRC	SST		—	10	—	—	—	—	10	10	—	*10*	—	—	10	**50**
Ad5 Neut. Antibody	CSR	VRC	SST		—	5	—	—	—	—	—	5	—	*5*	—	—	5	**20**
Cellular assays																		
ELISpot/ICS	CSR	FHCRC	Na Hep		—	60	—	—	60	—	—	—	—	—	*60*	60	60	**300**
ELISpot/ICS	CSR	VRC	Na Hep		—	40	—	—	—	—	40	40	—	*40*	—	—	40	**200**
Specimen storage																		
PBMC	CSR		Na Hep		—	60	—	—	60	—	120	70	—	110	40	100	150	**710**
Serum	CSR		SST		—	20	—	—	10	—	10	10	5	10	—	10	20	**95**
**Total**					**20**	**240**	**10**	**0**	**145**	**20**	**195**	**160**	**20**	**185**	**100**	**205**	**325**	**1625**
**56-Day total**					**20**	**260**	**270**	**270**	**415**	**435**	**370**	**160**	**180**	**365**	**465**	**205**	**325**	
Urine Collection																		
Urinalysis					X	—	—	—	X	—	—	—	X	—	—	—	—	
Pregnancy test					X	X	—	X	—	X	—	X	—	—	X	—	—	

CSR = Central Specimen Repository

HVTN Laboratory Program includes endpoint laboratories at Richmond, Duke, FHCRC, and SAIL-NICD. Richmond = Viral and Rickettsial Disease Laboratory, California Department of Health Services (Richmond, California, USA); Duke = Duke University Medical Center (Durham, North Carolina, USA); FHCRC/UW = Fred Hutchinson Cancer Research Center/University of Washington (Seattle, Washington, USA); SAIL-NICD = South African Immunology Laboratory–National Institute for Communicable Diseases (Johannesburg, South Africa)

Non-HVTN laboratories: VRC = Vaccine Research Center, National Institutes of Health (Bethesda, Maryland, USA)

Screening may occur over the course of several contacts/visits up to and including Day 0 prior to vaccination. Additional test performed at screening include syphilis test using serum samples.

Local labs may perform HIV diagnostic algorithms (following HVTN SOP) with pre-approval from the HVTN Laboratory Operations Division.

For viral assays, samples are sent to CL-Richmond; test to be performed if clinically indicated. Non-US sites may use local labs with pre-approval from the HVTN Laboratory Operations Division.

Local labs may assign appropriate alternative tube types for locally performed test.

Based on the number of responders at the primary humoral immunogenicity endpoint, Visit 10 (italics), lab assays may be performed on all participants for responses at Visits 5, 7, 8, 9, 12, and 13 for binding ELISA or neutralizing antibody.

Based on the number of responders at the primary cellular immunogenicity endpoints, Visits 10 and 11 (italics), lab assays may be performed on all participants for responses at Visits 5, 7, 8, 12, and 13 for ELISpot and ICS.

Molecular HLA typing may be performed on enrolled participants using cryopreserved PBMC collected at baseline, initially in participants who demonstrate vaccine-induced T-cell responses at post-vaccination time points.

FOR REVIEW ONLY

Appendix B: Procedures at HVTU

Visit:	**01**[a]	**02**	**03**	**04**	**05**	**06**	**07**	**08**	**09**	**10**	**11**	**12**	**13**	**Post**
Day:		D0	D14	D28	D42	D56	D70	D168	D182	D196	D210	D288	D364	
Month:		M0		M1		M2		M6		M7		M9.5	M12	
Procedure	Scr.	**VAC1**		**VAC2**		**VAC3**		**VAC4**						
**Study procedures**														
Signed screening consent (if used)	X	—	—	—	—	—	—	—	—	—	—	—	—	—
Assessment of understanding	X	—	—	—	—	—	—	—	—	—	—	—	—	—
Signed protocol consent	X	—	—	—	—	—	—	—	—	—	—	—	—	—
Medical history	X	—	—	—	—	—	—	—	—	—	—	—	—	—
Complete physical exam	X	—	—	—	—	—	—	—	—	—	—	—	X	—
Abbreviated physical exam	—	X	X	X	X	X	X	X	X	X	X	X	—	—
Risk reduction/pregnancy prevention counseling	X	X	X	X	X	X	X	X	X	X	X	X	X	—
Behavioral risk assessment	X	—	—	—	—	—	—	—	—	—	—	—	—	—
Confirm eligibility, obtain demographics, randomize participant	X	—	—	—	—	—	—	—	—	—	—	—	—	—
Social impact assessment	—	—	—	—	—	—	X	X	—	—	—	—	X	—
Outside testing and belief questionnaire	—	—	—	—	—	—	—	X	—	—	—	—	X	—
Concomitant medications	X	X	X	X	X	X	X	X	X	X	X	X	X	—
Intercurrent illness / adverse experience	—	X	X	X	X	X	X	X	X	X	X	X	X	—
HIV infection assessment/results[b]	X	—	—	—	—	X	—	X	—	—	—	X	X	—
**Local lab assessment**											—			
Urine dipstick	X	—	—	—	X	—	—	—	X	—	—	—	—	—
Pregnancy (urine or serum HCG)[c]	X	X	—	X	—	X	—	X	—	—	X	—	—	—
CBC, differential, platelet	X	—	X	—	X	—	X	—	X	—	—	X	X	—
ALT + creatinine	X	—	X	—	X	—	X	—	X	—	—	X	X	—
Syphilis test	X	—	—	—	—	—	—	—	—	—	—	—	—	—
**Vaccination procedures**											—			
Vaccination	—	X	—	X	—	X	—	X	—	—	—	—	—	—
Reactogenicity assessments[d]	—	X	—	X	—	X	—	X	—	—	—	—	—	—
**Post-study**														
Unblind participant	—	—	—	—	—	—	—	—	—	—	—	—	—	X

[a] Screening may occur over the course of several contacts/visits up to and including Day 0 prior to vaccination.

[b] Includes pre- and post-test counseling and follow-up contact to report results to participant.

[c] For female participants, pregnancy test must be performed on the day of vaccination prior to vaccination. Pregnancy test to determine eligibility may be performed at screening but must be performed on Day 0 prior to first vaccination. Serum pregnancy tests may be used to confirm the results of, or substitute for, a urine pregnancy test.

[d] Reactogenicity assessments performed daily for up to 3 days post-vaccination (see Section 14.1).

FOR REVIEW ONLY
